# Phosphorus and Potassium Mobilization from Rock-Derived Mineral Sources Using Bacteria and Microalgae: A Review

**DOI:** 10.3390/microorganisms14081761

**Published:** 2026-08-10

**Authors:** Viviane Simon, Júlia Lorenzato, Jéssica Mulinari, Edson Campanhola Bortoluzzi, Alan Rempel, Mateus Torres Nazari, Luciane Maria Colla

**Affiliations:** 1 Graduate Program in Civil and Environmental Engineering, Institute of Technology, University of Passo Fundo (UPF), Passo Fundo 99052-900, RS, Brazil; 105305@upf.br (V.S.); 170930@upf.br (J.L.); 2 Graduate Program in Agronomy, Faculty of Agricultural Sciences, Innovation and Business, University of Passo Fundo (UPF), Passo Fundo 99052-900, RS, Brazil; jessicamulinari15@gmail.com (J.M.); edsonb@upf.br (E.C.B.); 3 Chemical Engineering Program, Institute of Technology, University of Passo Fundo (UPF), Passo Fundo 99052-900, RS, Brazil; rempel@upf.br

**Keywords:** mining by-product, microbial coculture, microbial interactions, nutrient bioavailability, bioinoculants, beneficial microorganisms, plant growth promotion

## Abstract

Basalt rock powder (BRP) has been evaluated as an alternative to soluble fertilizers owing to its phosphorus (P) and potassium (K) content, although their association with low-solubility minerals may restrict availability to plants. This review synthesized evidence on microbial P and K mobilization from rock-derived minerals and examined its relevance to BRP. Scopus and Web of Science were searched for English-language publications through 2025. After merging and duplicate removal, 98 records were sequentially screened, and 33 experimental studies were included. Bacterial evidence predominated, mainly from phosphate rock, hydroxyapatite, tricalcium phosphate, feldspar, and other non-basalt substrates. Studies involving photosynthetic microorganisms covered fewer strains and mineral sources. Direct basalt evidence was limited to K-release measurements from crystalline basalt by cyanobacteria and did not represent mobilization from BRP under soil conditions. Differences in mineral composition and loading, cultivation conditions, incubation periods, analytical fractions, and calculation bases limited direct comparisons. Mineral-P mobilization was associated with acidification, organic acids, and ligand-cation interactions, whereas phosphatase activity represented organic-P mineralization. K-related evidence included acidification, ion exchange, surface alteration, and feldspar-binding proteins. Mixed phototrophic-bacterial systems were evaluated mainly through metabolic interactions or soil and plant responses. Direct comparisons with monocultures were uncommon, and no included study quantified P or K release from BRP by a defined microalgae-bacteria consortium. Further research could clarify the applicability of microbial mobilization to BRP by linking its mineralogical characterization and controlled nutrient-release measurements with formulation stability and responses under soil and field conditions.

## 1. Introduction

Agricultural systems are expected to meet the food demand of a global population approaching 10 billion by 2050 while reducing the environmental impacts associated with agricultural production [1]. Conventional fertilizers contribute to crop productivity, although applications exceeding crop demand or poorly synchronized with plant uptake may increase nutrient losses. Hina [2] discusses fertilizer management, soil properties, precipitation, irrigation, and crop characteristics as factors relevant to nutrient leaching. In this context, basalt rock powder (BRP), a by-product of basalt mining and crushing operations [3], represents a mineral amendment under investigation [4] and a potential complementary source of P and K [5]. These nutrients are essential for plant growth, but their occurrence mainly in apatite, feldspars, and micas may restrict their immediate availability [6]. The agronomic relevance of basalt-derived materials may also depend on mineral composition, particle properties, application rate, soil characteristics, crop, and duration of application [7,8,9].

Microbial interactions with mineral surfaces may contribute to mineral alteration and nutrient mobilization through acidification, ligand-mediated reactions, surface attachment, and the production of extracellular compounds [10,11]. Bacteria associated with P and K mobilization can produce organic acids, siderophores, extracellular polymeric substances, and enzymes involved in nutrient acquisition [12,13,14,15,16,17]. Microalgae and cyanobacteria may also participate in nutrient-acquisition processes through phosphatase activity, extracellular compounds, and other physiological responses [18,19]. The expression and relative contribution of these processes may depend on the microorganism, mineral substrate, and cultivation conditions [4,20,21,22].

The combined use of bacteria and photosynthetic microorganisms has been proposed as an approach for exploring metabolite exchange, biofilm development, and localized physicochemical changes at mineral interfaces [23,24,25,26,27,28]. The contribution of these interactions to P and K mobilization is not yet clearly established, particularly in BRP-containing systems and in direct comparisons between mixed cultures and their corresponding monocultures [29]. Differences in mineral sources, microbial groups, experimental conditions, analytical approaches, and response variables require careful consideration when evaluating the available evidence and its relevance to BRP-based systems.

Studies differ in the microorganisms, mineral sources, cultivation conditions, analytical approaches, and response variables evaluated, which limits direct comparisons and the interpretation of their relevance to BRP-based systems. This review aimed to critically synthesize the available evidence on microbial P and K mobilization from rock-derived mineral sources and identify its relevance and limitations for the investigation of BRP. The review addressed the following research questions: (i) Which mineralogical and experimental factors are associated with P and K release from rock-derived mineral sources? (ii) What direct and indirect evidence is available for P and K mobilization by bacteria, photosynthetic microorganisms, and mixed microbial systems? (iii) To what extent can results obtained with non-basalt mineral substrates inform the investigation of BRP? (iv) Which methodological, agronomic, and implementation gaps should be addressed in the development of BRP-based microbial products?

## 2. Content Analysis

This review was designed to synthesize and critically interpret experimental findings obtained across different mineral substrates, microorganisms, and experimental systems. Bibliographic data were processed using RStudio (version 2025.05.1+513; Posit Software, PBC, Boston, MA, USA) with the Bibliometrix package. Scopus and Web of Science were selected as the source databases based on the availability of structured bibliographic metadata compatible with the data-import and integration procedures used in this analysis [30]. Their multidisciplinary coverage corresponded to a topic spanning microbiology, agriculture, mineralogy, and environmental sciences [31], while differences in journal indexing provided complementary bibliographic records [32].

**Figure 1:**
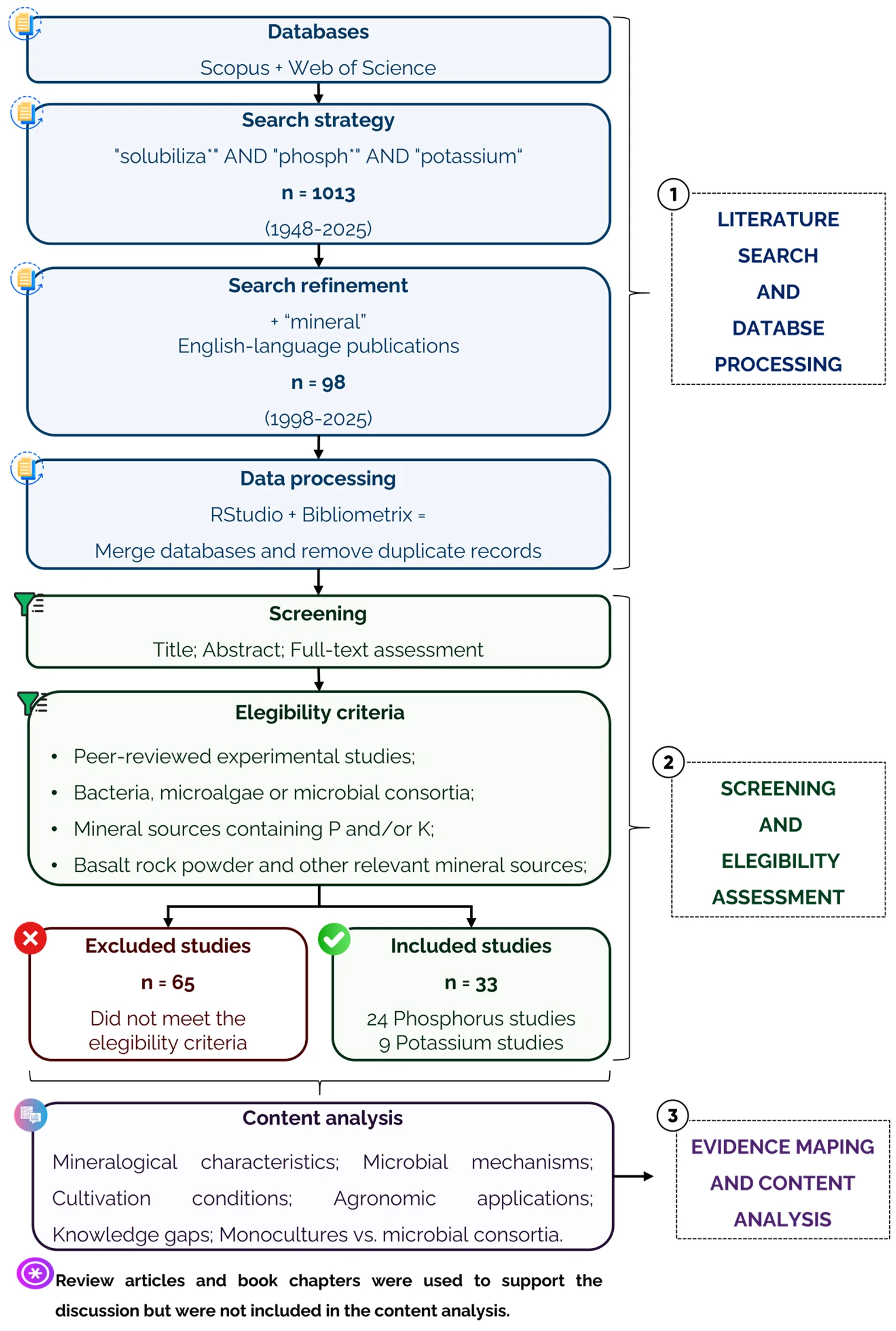
The methodological procedure used for the literature search, study screening, eligibility assessment, and content analysis.

The search strategy used the keywords “solubiliza*” AND “phosph*” AND “potassium”, retrieving 1013 publications indexed between 1948 and 2025. To focus on studies involving mineral nutrient sources, the search was refined by including the keyword “mineral” and limiting the search to English-language publications. Records retrieved from both databases were merged, and duplicate publications were removed, resulting in 98 unique records published between 1998 and 2025. The overall procedure is presented in Figure 1.

**Figure 2:**
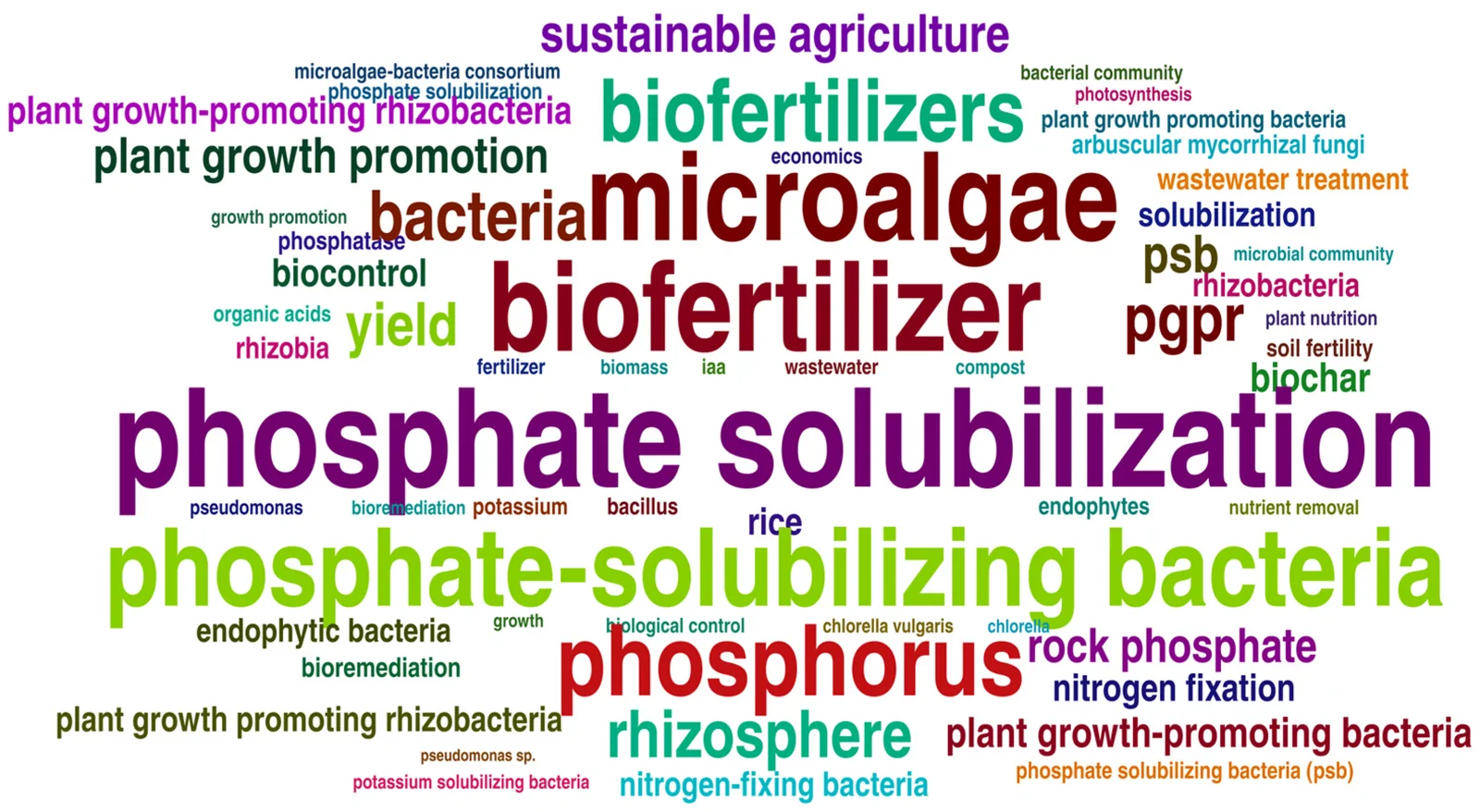
A word cloud generated from the search keywords.

Before study screening, the authors’ keywords and Keywords Plus from the 98 unique records obtained after database merging and duplicate removal were used to generate the word cloud presented in Figure 2. This complementary descriptive analysis was used to support the preliminary identification of thematic areas represented in the retrieved literature and the organization of the subsequent screening and content analysis. Eligibility decisions remained based on the predefined inclusion and exclusion criteria.

Within this structured narrative review, the 98 records were screened sequentially by title, abstract, and, when necessary, full-text assessment. Peer-reviewed experimental studies involving bacteria, microalgae, cyanobacteria, or mixed microbial systems associated with P and/or K mobilization from mineral sources were included. Studies examining BRP and other P- or K-bearing minerals were considered. Conference papers, short communications, and publications unrelated to microbial P or K mobilization were excluded. Review articles and book chapters were not incorporated into the analytical dataset but were consulted for background information and interpretation of the experimental literature.

Following screening, 65 publications did not meet one or more eligibility criteria, resulting in 33 experimental studies retained for analysis. Among these, 24 primarily addressed P mobilization and nine primarily addressed K mobilization.

Data extracted from the full texts covered microorganisms, mineral substrates, cultivation conditions, experimental approaches, nutrient-mobilization measurements, agronomic applications, and reported research gaps. This information supported the classification of the studies according to mineral-source relevance, type of evidence, and experimental setting. Mineral substrates were grouped as basalt or BRP, non-basalt P- or K-bearing minerals, or systems without a defined mineral source. Evidence categories comprised culture-based nutrient release, mechanistic or mineralogical responses, soil or plant responses, and contextual or indirect observations. Experimental settings comprised laboratory, controlled cultivation, greenhouse or pot, and field conditions. Comparisons between mixed cultures and monocultures were considered when direct experimental assessments were available.

A formal quality or risk-of-bias score was not applied. Methodological characteristics, including mineral characterization, experimental controls, cultivation conditions, sampling procedures, analytical methods, measurement units, and correspondence between the measured response and its interpretation, were considered qualitatively when interpreting the consistency, limitations, and applicability of the evidence. Study counts were used to describe the distribution of the literature and not as indicators of evidence strength.

## 3. Rock Powder as a Strategy for Partial Replacement of Chemical Fertilizers

Rock powder (RP) has been evaluated as a mineral input for the partial replacement of soluble fertilizers. Its chemical and mineralogical composition varies with geological origin and may include feldspars, micas, pyroxenes, olivine, apatite, and amorphous phases containing K, Ca, Mg, Fe, P, and Si. Unlike soluble fertilizers, RP contains nutrients within solid phases that undergo weathering or dissolution before these elements are released into the soil solution and subsequently redistributed among soil fractions [5,33].

Although P and K participate in essential plant physiological processes, their retention within mineral structures may restrict immediate availability. Phosphorus participates in energy transfer through ATP and ADP, nucleic acid synthesis, phosphorylation reactions, and phospholipid membrane formation [34]. Potassium occurs mainly as a cellular ion and contributes to osmotic adjustment, stomatal movement, charge balance, enzyme activation, protein synthesis, and photoassimilate transport [35]. The potential agronomic contribution of an RP cannot be inferred solely from its total P and K contents [5,33] and may depend on the amounts released under the evaluated soil [36] and cultivation conditions [6].

Phosphorus and potassium occur in mineral phases with different structures and weathering behavior [37,38,39,40]. Their release varies with mineralogical and environmental conditions and may not coincide with crop nutrient demand during a growing cycle [5,6,33,36].

### 3.1. Mineralogy and Geochemical Reactivity of P and K Mineral Sources

Phosphorus is commonly associated with apatite, whereas K occurs in feldspars and micas. Differences in mineral composition and structure may result in distinct processes and periods of P and K release [38,39,40]. In agricultural soils from the Araguaia River valley, Volf et al. [41] associated K reserves with the occurrence and distribution of feldspars, micas, and their weathering products. Structural K may therefore represent a potential nutrient reserve, but its presence does not determine the amount released within a crop cycle, particularly considering the slower contribution of non-exchangeable forms to plant K supply [42]. This distinction corresponds to the separation of soil K into solution, exchangeable, non-exchangeable, and structural or mineral pools [43].

Apatite is commonly represented by the formula Ca_5_(PO_4_)_3_X, where X may correspond to F^−^, OH^−^, or Cl^−^. Its structure contains phosphate tetrahedra coordinated with Ca^2+^, and substitutions within the crystal structure can affect its chemical properties [38]. In experiments with igneous and sedimentary apatites, the release of Ca, P, and F increased as pH decreased from 6 to 2. The dissolution rates also varied with apatite composition, temperature, and solution saturation [44]. Thus, P release from apatite depends on the mineral composition and the chemical conditions evaluated [38,39,40,44].

Orthoclase and microcline have the general composition KAlSi_3_O_8_ and consist of three-dimensional structures formed by interconnected SiO_4_ and AlO_4_ tetrahedra. Within these structures, K^+^ compensates for the negative charge associated with the substitution of Al^3+^ for Si^4+^ [40,45]. Experimental and mineralogical studies associate K release from feldspars with surface protonation, hydrolysis, ion exchange, and structural alteration, which may also contribute to the formation of secondary mineral phases [40,46,47].

Micas, including biotite and muscovite, consist of tetrahedral–octahedral–tetrahedral layers containing interlayer K^+^. Potassium release occurs through exchange reactions at exposed surfaces and edges and through structural alteration during weathering [39,40,47]. The loss of interlayer K has been associated with the formation of vermiculitic or interstratified phases, with the resulting products varying according to mica composition and environmental conditions [47]. Because K occupies structural positions in feldspars and interlayer positions in micas, its release may remain limited during short-term experiments [40,48].

In addition to nutrient-bearing minerals, basalt may contain volcanic glass and different crystalline phases [45,46]. Basaltic glass lacks long-range structural order [49], whereas individual crystalline phases present specific structures and compositions [45,46]. Experimental studies indicate that differences in pH, temperature, and solution composition may affect the dissolution of basaltic glass and crystalline basalt [49,50]. Rock powders with similar elemental compositions may therefore present different dissolution behavior according to their relative proportions of glass and crystalline minerals [50].

The effect of pH also varies among silicate phases. Experimental and kinetic studies reported increased dissolution under acidic conditions and, for some silicates, under alkaline conditions, with lower rates near neutral pH [45,46,49,50,51,52]. Under acidic conditions, protonation of oxygen bonds at mineral surfaces has been associated with the release of K^+^, Ca^2+^, Mg^2+^, and Na^+^ [45,46]. Under alkaline conditions, reactions involving OH^−^ may contribute to Si-O bond cleavage [46,47]. These studies indicate that the dissolution pattern reported for one silicate phase may not represent that of other phases.

Besides pH, the ionic composition of the surrounding solution can affect mineral dissolution. Mesfin et al. [53] reported that CaCl_2_ and MgCl_2_ increased the dissolution rate of basaltic glass but decreased the dissolution rate of labradorite under the same experimental conditions. The kinetic data compiled by Palandri and Kharaka [52] also showed differences among apatite, feldspars, micas, and other silicates according to mineral composition, pH, and temperature. These findings indicate that comparisons of nutrient-release data require information on both the mineral phases and the experimental conditions [52,53].

### 3.2. Mineralogical and Physicochemical Characterization of Rock Powders

Total elemental composition does not identify the mineral phases in which P and K occur. Mineralogical and physical characterization can therefore complement chemical analysis by providing information on nutrient-bearing phases, particle properties, and the distribution of elements within RP [3,54].

X-ray diffraction (XRD) can identify crystalline phases such as apatite, feldspars, micas, and pyroxenes and can also detect secondary minerals formed during weathering. This information distinguishes P associated with apatite from K associated with feldspars or micas, although the identification of a mineral phase does not indicate the amount of nutrient released under soil conditions. Quantitative interpretation may also be affected by overlapping diffraction peaks, mineral substitutions, and the presence of amorphous material [55,56].

Scanning electron microscopy coupled with energy-dispersive spectroscopy (SEM-EDS) provides information on particle morphology, fractures, surface features, and localized elemental composition [3]. These observations can indicate whether P or K is distributed across different particles or concentrated in specific mineral domains. However, EDS provides localized and semi-quantitative information and does not replace bulk elemental analysis. Techniques such as ICP-OES or ICP-MS can be used to determine the total elemental composition of the material after sample digestion [57].

Particle-size distribution and specific surface area may provide complementary information on the fraction of the solid exposed to the surrounding solution. Smaller particle sizes can be associated with a greater surface area per unit mass and more frequent contact between mineral surfaces and the soil solution [58]. The resulting nutrient release may also depend on the composition and reactivity of the exposed phases [33,36]. Particles of similar size may therefore present different P or K release when these elements occur in minerals with different weathering behavior.

Mineralogical, chemical, and physical analyses provide complementary information on RP properties. XRD identifies crystalline phases, whereas SEM-EDS examines morphology and localized elemental distribution [46]. ICP-based methods determine bulk elemental composition, while particle-size and surface-area analyses describe the solid fraction potentially available for reaction. These measurements can be interpreted together with dissolution assays, soil nutrient fractions, plant nutrient concentrations, biomass, and yield data to examine possible relationships between material properties and agronomic responses [54]. Nevertheless, no individual characterization technique establishes P or K availability under soil conditions.

### 3.3. Agronomic Performance as Influenced by Mineralogy and Application Conditions

Agronomic studies were separated according to the relationship of the evaluated material with basalt. Table 1 presents studies that directly applied basalt, BRP, or basalt-derived materials, whereas Table 2 presents agronomic evidence obtained with other rock-derived mineral amendments. The studies differed in application rate, particle-size distribution, soil, crop, experimental setting, and duration.

A common feature of the studies in Table 1 was the simultaneous assessment of several soil or plant responses following basalt application, rather than the direct quantification of P or K released from the material. Changes in soil P, leaf P, plant nutrient concentrations, or dry matter were observed in greenhouse and field experiments [8,9,59], while the mesocosm studies addressed crop yield, N leaching, and shoot Si [6,7]. These endpoints indicate that basalt application may be associated with agronomic or soil responses, but they represent different stages of the soil–mineral–plant system. Changes in soil nutrient fractions do not necessarily correspond to plant acquisition, and changes in biomass or yield do not identify the nutrient or process responsible for the plant response.

The relationship with P was more apparent in studies reporting soil P, leaf P, or P concentrations in plant dry matter [8,9,59]. These measurements provide indirect evidence of changes in P availability but do not distinguish P released from apatite or other basaltic phases from P already present in the soil. The evidence for K mobilization was more restricted. Although plant K tended to increase after application of hydrothermally altered basalt [59], the amount of K released from feldspars or micas was not quantified. None of the studies connected mineral-phase alteration, changes in defined soil P or K pools, plant nutrient acquisition, and crop performance within the same experimental system. Consequently, the available agronomic responses cannot be interpreted as direct measurements of P or K mobilization from basalt.

The studies also do not indicate a consistent relationship between particle size, application rate, and agronomic response. Responses were reported for relatively coarse crushed basalt [7], hydrothermally altered material [59], and BRP containing different proportions of fine and coarse particles [6,8,9]. These materials differed simultaneously in mineralogy, granulometry, applied quantity, soil, crop, and duration, preventing the isolation of particle size as the factor responsible for the observed response. The studies also do not indicate a consistent relationship between particle size, application rate, and agronomic response. Responses were reported for relatively coarse crushed basalt [7], hydrothermally altered material [59], and BRP containing different proportions of fine and coarse particles [6,8,9]. These materials differed simultaneously in mineralogy, granulometry, applied quantity, soil, crop, and duration, preventing the isolation of particle size as the factor responsible for the observed response. Application regimes ranged from repeated treatments of 1–8 t ha^−1^, reaching cumulative amounts of up to 16 t ha^−1^ [59], to single applications of 33–99 t ha^−1^ [8,9], 50 t ha^−1^ [6], or 10 kg m^−2^, equivalent to 100 t ha^−1^ [7]. Responses obtained under these different application regimes cannot be interpreted as a common dose–response relationship or transferred directly among basalt products.

**Table 1:** Direct agronomic evidence from studies applying basalt, basalt rock powder, or basalt-derived mineral amendments.

Basalt Material	Application Rate	Particle Size	Crop	Experimental Setting	Reported Agronomic Response	Interpretation and Limitation	Reference
Basalt rock powder	50 t ha^−1^	2.38% <32 µm; 14.73% at 32–63 µm; 59.82% at 63–250 µm; 22.30% at 250–500 µm; 0.52% at 500–2000 µm; 0.25% >2000 µm	*Solanum tuberosum*	Mesocosm; alkaline soil	No statistically significant reduction in potato growth or yield was detected; yield tended to increase, and N leaching decreased by approximately 45%.	The study provides direct agronomic evidence for BRP under mesocosm conditions. The response does not identify whether P or K release contributed to plant performance, and the results do not represent field-scale application.	[6]
Hydrothermalized basalt powder (metabasalt)	0, 1, 2, 4, and 8 t ha^−1^; treatments were reapplied, reaching cumulative rates of up to 16 t ha^−1^	100% <0.84 mm; 50% <0.30 mm	Native grassland	Field; Acrisol, pH 4.9	Nutrient transfer from soil to plants increased; P, K, Ca, and Mg tended to increase in plant dry matter, and a dry-matter response was observed at 8 t ha^−1^.	The study provides field evidence for a basalt-derived amendment. The hydrothermally altered material may differ mineralogically from unaltered BRP, and the contribution of individual mineral phases was not determined.	[59]
Crushed basalt	10 kg m^−2^	p80 = 1250 µm	*Sorghum bicolor*	Controlled mesocosm; mildly acidic clay-loam agricultural soil, pH 6.6	Seed yield increased by 21 ± 9.4%, and shoot Si increased by 26 ± 5.4%, without additional P or K fertilizer.	The study provides direct evidence for crushed basalt under controlled soil conditions. The plant response cannot be attributed specifically to P or K release, and the applied material was coarser than some BRP products.	[7]
Basalt rock powder (RM Fortaleza)	0, 33, 66, and 99 t ha^−1^	38.60% <0.075 mm; 31.97% retained at 0.075 mm; 21.15% at 0.30 mm; 7.93% at 1 mm; 0.35% at 2 mm	Soybean and maize	Greenhouse; clay soil, pH 4.5, and sandy-clay-loam soil, pH 5.2	Changes in plant dry matter, soil pH, soil Ca and P concentrations, and leaf P content were reported.	The study provides direct evidence for BRP application in two soils. Changes in P availability and plant growth may have occurred together with changes in soil pH and Ca availability, preventing attribution of the response exclusively to P release from BRP.	[8]
Basalt rock powder	0, 33, 66, and 99 Mg ha^−1^	8.0% retained at 0.85 mm; 28.5% at 0.30 mm; 63.5% <0.30 mm	Soybean and maize	Greenhouse; sandy-textured and clay soils	Changes in soybean and maize dry matter and in soil Ca, Mg, P, and pH were reported.	The responses differed with soil and application rate. Changes in soil P and plant dry matter do not independently identify the mineral phase responsible for nutrient release or separate nutrient supply from soil-acidity effects.	[9]

P, phosphorus; K, potassium; Ca, calcium; Mg, magnesium; N, nitrogen; Si, silicon; BRP, basalt rock powder. Mg ha^−1^ denotes megagrams per hectare. The notation p80 indicates the particle size below which 80% of the material occurs. The table includes studies in which basalt, BRP, or a basalt-derived material was applied directly. The reported responses provide agronomic evidence for basalt-based amendments but do not necessarily identify the mineral phase responsible for nutrient release or distinguish nutrient-release effects from changes in soil pH and other soil properties. Differences in material composition, application rate, particle-size distribution, soil, crop, experimental setting, and duration limit direct comparisons among studies.

Recent field experiments provide additional context but do not resolve this attribution. Beerling et al. [60] combined repeated basalt application with measurements of carbon removal, soil properties, and maize-soybean responses, whereas Skov et al. [61] examined initial agronomic responses in spring oat. Holden et al. [62] detected basalt weathering in a tropical sugarcane system, although measured carbon removal remained limited under the evaluated conditions. Together, these studies indicate that mineral weathering, changes in soil chemistry, nutrient availability, crop response, and carbon removal are related but not equivalent outcomes. Their relationships vary with basalt composition, soil properties, climate, application history, and experimental duration.

The direct agronomic evidence suggests that basalt-based amendments may coincide with changes in soil properties, plant nutrient composition, or crop performance, but it does not establish a consistent causal sequence from basalt dissolution to P or K release, plant acquisition, and yield. This limitation is particularly relevant to BRP-based microbial products. Evidence of microbial contribution would depend on experimental designs capable of distinguishing the responses to BRP, the microbial component, and their combined application, while relating mineral alteration to defined P and K fractions and plant acquisition.

Studies using non-basalt mineral amendments provide complementary evidence on how mineral composition, application rate, particle size, soil, crop, and experimental duration may affect agronomic responses. These studies are presented separately in Table 2, as their results do not constitute direct evidence for basalt or BRP.

Studies applying non-basalt mineral amendments were retained as contextual evidence for interpreting how nutrient-bearing phases and soil conditions may influence agronomic responses. Their results were considered separately from direct evidence involving basalt or BRP, as summarized in Table 2. Together, these studies contribute information on material-specific responses, residual nutrient supply, soil-amendment interactions, and combined mineral–microorganism treatments.

**Table 2:** Agronomic evidence obtained with non-basalt rock-derived mineral amendments under different application conditions.

Mineral Material	Application Rate	Particle Size	Crop	Experimental Setting	Reported Agronomic Response	Interpretation and Limitation	Reference
Granite and diorite powders	Granite: 0.625, 1.25, 2.5, 7.5, 15, and 30 g pot^−1^; diorite: 0.3125, 0.625, 1.25, 2.5, 7.5, and 15 g pot^−1^	<1180 µm	Wheat (*Triticum aestivum*)	Glasshouse; responsive, highly weathered Western Australian soil	Granite applied at rates ≥7.5 g pot^−1^ increased wheat biomass and total shoot K; tissue K concentration increased at rates >15 g pot^−1^. Diorite did not produce a statistically significant response.	The contrasting responses to granite and diorite indicate that application rate alone does not account for agronomic performance. Mineralogical composition and K-bearing phases should be considered, and the pot-scale results cannot be transferred directly to basalt-based amendments.	[48]
Sedimentary phosphate rock	118 and 250 kg P ha^−1^	Mainly sand-sized; 25% <50 µm and 4% <2 µm	Pampa grassland and forage	Field; Typic Paleudult, initial pH 4.5; 20-year experiment	Cumulative dry matter was 4% greater than the no-P control for the phosphate-rock legacy treatment. Soluble P combined with limestone resulted in 22% greater dry matter and fivefold greater P-use efficiency than phosphate rock.	The study provides long-term field evidence for residual P contribution from sedimentary phosphate rock. Its composition and dissolution behavior differ from those of apatite dispersed within basalt, limiting direct extrapolation to BRP.	[63]
Agate powder residue	0, 1, 2, 4, and 8 t ha^−1^; application repeated after approximately 47–48 weeks	90% <21.8 µm	Native grassland	Field; acidic, low-fertility Acrisol	Lower application rates increased soil pH and reduced potential acidity and exchangeable Al, whereas higher rates decreased soil-available K and K in plant dry matter.	The direction of the response differed with application rate. The reduction in soil and plant K shows that the presence of a finely divided rock-derived material does not necessarily correspond to greater K availability.	[64]
Siltstone rock dust	0, 4, 8, 16, and 32 t ha^−1^	100% <2.0 mm; 97.84% <0.84 mm; 93.31% <0.30 mm	Maize and bean	Greenhouse; Quartzipsamment and Oxisol	At 4 t ha^−1^, maize shoot dry matter increased by 40% and 200% in the Quartzipsamment and Oxisol, respectively; bean dry matter increased by 126% and 283%.	The magnitude of the response differed between crops and soils. Biomass measurements do not independently identify P or K release, and the mineralogical composition of siltstone differs from that of basalt.	[65]
Natural Moroccan rock phosphate combined with phosphate-solubilizing bacteria	1 g rock phosphate kg^−1^ soil-peat mixture	377 µm	Tomato (*Solanum lycopersicum*)	Greenhouse; soil-peat mixture (2:1), with the soil fraction containing 87.32% sand and presenting pH 8.57	Bacterial inoculation combined with rock phosphate increased shoot length by up to 45.51%, root length by up to 54.52%, shoot and root fresh weight by up to 63.68% and 72.96%, and shoot and root dry weight by up to 56.26% and 78.13%, respectively.	The study provides plant-response evidence for bacterial inoculation combined with phosphate rock. It does not represent direct evidence for BRP, and the plant response cannot be attributed exclusively to microbial P release when other plant-growth-promoting traits were also present.	[66]

P, phosphorus; K, potassium; Al, aluminum. Agronomic responses refer to comparisons with the corresponding controls under the conditions reported in each study. These materials provide contextual evidence concerning the effects of mineral composition, application rate, particle size, soil, crop, and experimental duration on agronomic responses. Granite, diorite, sedimentary phosphate rock, agate residue, siltstone, and natural phosphate rock differ mineralogically from basalt and should not be treated as direct evidence for BRP. Values obtained with different materials, experimental settings, analytical endpoints, and cultivation periods are not directly comparable.

The comparison between granite and diorite provides evidence that mineral identity can influence K-related responses under the same soil, crop, and cultivation conditions. Granite increased wheat biomass and shoot K at selected application rates, whereas diorite produced no statistically significant plant response [48]. This contrast supports the inclusion of mineralogical characterization when interpreting the nutrient contribution of silicate amendments. Agate residue altered soil acidity but was associated with lower soil-available and plant K [64], showing that changes in soil chemical conditions and K acquisition may follow different patterns. Responses to siltstone varied between crops and soils [65], indicating that crop characteristics and soil properties should be considered together with the amendment composition.

The K-related studies also illustrate the value of combining soil and plant measurements. Soil-extractable K describes changes in a chemically defined soil fraction, while shoot K provides information on nutrient acquisition. Their joint assessment can help distinguish changes in the soil from those reflected in plant nutrition. The contrasting responses to granite, diorite, and agate residue [48,64] further indicate that mineral composition should accompany total K content and particle size when evaluating K-bearing materials. For BRP studies, these findings support the characterization of K-bearing phases together with measurements of soil K fractions and plant K acquisition.

The phosphate-based studies contribute information on the temporal and biological aspects of mineral P use. The residual dry-matter response observed after 20 years of phosphate-rock application [63] indicates that slowly available mineral sources may contribute over periods extending beyond an individual crop cycle. Such residual contributions may be agronomically relevant across successive cultivation cycles while remaining insufficient to meet crop nutrient demand within a shorter experimental period [33,48,63]. The simultaneous use of soluble P and limestone in the comparison treatment prevents attribution of the response to P solubility alone, but it also demonstrates the relevance of soil-acidity management to the agronomic evaluation of mineral P sources. Reactions involving Ca-containing phases may further influence the P fractions present in soil [67,68].

The greenhouse study combining Moroccan phosphate rock with bacterial inoculation extends this evidence to a mineral–microorganism system [66]. The reported tomato responses support the evaluation of microbial inoculation together with a defined mineral nutrient source. Other plant-growth-promoting traits of the bacterial strains may have contributed to the response, so P mobilization represents one possible component of the observed effect. This study is relevant to BRP-based research by illustrating the need to assess nutrient mobilization together with microbial traits, soil responses, and plant performance.

Table 1 and Table 2 provide complementary forms of evidence. The basalt studies establish direct material relevance, while the non-basalt studies contribute information on mineral-specific behavior, soil and crop mediation, residual nutrient supply, and microbial co-application. These findings can guide the selection of mineralogical analyses, soil nutrient fractions, plant measurements, treatment controls, and experimental duration in BRP-based studies. Quantitative responses should remain associated with the material and conditions in which they were obtained, but the experimental relationships identified across these studies provide a basis for evaluating microbial P and K mobilization in the following sections.

## 4. Bacterial Roles in Phosphorus and Potassium Mobilization

The bacterial literature included studies developed for different experimental purposes, from initial screening to the investigation of mineral interactions and agronomic responses. To distinguish these forms of evidence, the studies were organized according to mineral source, relevance to BRP, experimental setting, and measured response. Solid-medium assays were used to identify solubilization phenotypes [12,69,70], whereas liquid cultivation allowed soluble P or K to be measured during interaction with mineral substrates [14,16,17,71,72]. Mineral-surface analyses provided information on attachment and structural alteration [15,16], while molecular approaches examined gene-expression responses and mineral-binding proteins [14,15,17]. Soil and plant experiments addressed nutrient availability, plant acquisition, biomass, or yield [13,16,69,71,73,74]. These approaches represent distinct analytical endpoints. Halo formation indicates a solubilization phenotype, soluble-nutrient concentrations describe accumulation in the liquid phase, and mineralogical analyses identify changes associated with mineral surfaces. Table 3 presents the bacterial studies according to mineral source, relationship to BRP, experimental setting, measured response, and type of evidence.

### 4.1. Experimental Evidence for Bacterial P and K Mobilization

The studies summarized in Table 3 show an uneven distribution of evidence between P and K. Bacterial P mobilization was examined using phosphate rock [71,73,75], hydroxyapatite [14], tricalcium and aluminum phosphates [12,72], and Fe phosphate [69], allowing bacterial responses to be evaluated across chemically distinct P sources. Studies concerning K were concentrated mainly on feldspar and other K-bearing silicates [12,13,15,16,17,76]. The P literature therefore covers a broader range of mineral substrates, whereas the K literature provides more focused evidence for bacterial interactions with silicate minerals.

The comparison of Ca_3_(PO_4_)_2_ and AlPO_4_ by Damo et al. [72] provides an internally consistent assessment of substrate-dependent P mobilization. *Enterobacter* sp. LG7 was cultivated with both compounds under the same principal experimental conditions, and different soluble-P concentrations were reported. This contrast associates the bacterial response with the chemical characteristics of the phosphate source more directly than comparisons among independent studies. It also supports the use of conventional screening substrates for identifying P-solubilization phenotypes, followed by evaluation with the mineral source relevant to the intended application.

The P studies provided complementary evidence at different experimental levels. Soluble-P accumulation was measured during cultivation of a *Pseudomonas-Serratia* coculture with phosphate rock [71] and *Paenibacillus sonchi* SBR5 with hydroxyapatite [14]. Gene-expression measurements were also included in the latter study, relating nutrient release to bacterial responses during mineral interaction. *Bacillus megaterium* Ag87 and *Lysinibacillus* sp. Ag94 were evaluated through plate assays and subsequent maize experiments with FePO_4_·2H_2_O [69], connecting an initial solubilization phenotype with soil and plant responses. These studies describe different stages of bacterial–mineral interaction, although the complete sequence from mineral alteration to nutrient release and plant acquisition was rarely examined within a single experimental system.

The K studies provided a comparatively detailed assessment of selected bacterial-feldspar systems. Investigations with *Bacillus aryabhattai* SK1-7 combined soluble-K measurements with extracellular and mineral-surface responses [15,16]. *Priestia megaterium* NK851 was evaluated through K-release measurements and the identification of proteins associated with feldspar binding [17], while *Pseudomonas aeruginosa* A10 was examined through culture-based mobilization followed by cotton cultivation in saline-sodic soil [13]. The integration of chemical, mineralogical, molecular, and plant measurements across these studies indicates several analytical approaches through which bacterial interactions with K-bearing silicates can be examined. Quantitative results remain specific to each bacterial-mineral system and its experimental conditions.

*Bacillus* and related genera were represented across more mineral sources and experimental settings than *Pseudomonas*, *Enterobacter*, and *Paenibacillus* [15,16,17,69,70,73,75,76]. This distribution indicates broader experimental coverage rather than greater mobilization capacity at the genus level. The most informative comparisons were those performed within the same study, including comparisons between mineral substrates [72], incubation periods [17], individual and combined inocula [69], or culture-based and plant responses [13,69]. Such designs allow particular sources of variation to be examined while maintaining other experimental components.

For the investigation of BRP, the bacterial literature provides evidence that the identity of the nutrient-bearing phase should be considered together with the measured microbial and mineral responses. The phosphate studies support substrate-specific evaluation of P release, whereas the feldspar studies provide approaches for connecting soluble K with microbial attachment and mineral-surface responses. Applying these complementary measurements to characterized BRP may help determine how bacterial interactions with apatite, feldspars, and other constituent phases contribute to P and K mobilization within a polymineralic material.

### 4.2. Metabolic, Genetic, and Mineral-Surface Processes

Organic acids were among the extracellular compounds most frequently examined in bacterial-mineral systems. Gluconic acid was detected during P mobilization by the *Pseudomonas-Serratia* coculture [71] and *Paenibacillus sonchi* SBR5 [14], whereas citric, oxalic, malic, acetic, and other acids were reported in systems containing phosphate rock or feldspar [13,15,71]. Guo et al. [77] similarly related rock-phosphate solubilization by *Serratia* sp. to medium acidification and mineral-surface alteration. These findings indicate that organic-acid production can be examined as part of a sequence involving changes in solution chemistry, interactions with mineral-associated cations, and nutrient accumulation in the liquid phase. The relative contribution of individual acids can be investigated by relating their concentration and dissociation characteristics to temporal changes in pH, soluble P or K, and mineral-surface composition.

The metabolic origin of these compounds provides additional information for interpreting bacterial responses. Gluconate can be formed through membrane-associated glucose dehydrogenase using pyrroloquinoline quinone as a cofactor, and genes associated with PQQ biosynthesis have been examined in phosphate-solubilizing bacteria [78]. Wang et al. [79] used pqqC as a marker related to inorganic-P solubilization and distinguished it from phoD, which was associated with organic-P mineralization. Other extracellular acids originate from different reactions within carbon metabolism [14,15,71]. The carbon source can consequently influence biomass formation and the profile of compounds produced during mineral interaction. Future comparisons among carbon sources may help relate a particular metabolic profile to nutrient release rather than treating total acidification as a uniform bacterial response.

The feldspar studies provide evidence that reactions at the bacterial–mineral interface complement changes measured in the bulk solution. Feldspar-binding proteins were identified during the interaction of *Priestia megaterium* NK851 with feldspar [17], while extracellular polysaccharides and mineral-surface alteration were examined in *Bacillus aryabhattai* SK1-7 [15]. Using in situ microscopy, Han et al. [80] related the attachment and biofilm development of *Pseudomonas aeruginosa* PAO1 on K-feldspar to localized dissolution and K release. Biofilm matrices can retain cells, water, protons, and ligands near mineral surfaces [11,81], providing a spatial context for reactions that may not be represented by the final pH of the surrounding medium. Measurements combining microscopy, biofilm development, extracellular compounds, solution chemistry, and mineral-surface alteration can therefore relate bacterial establishment at the interface to the progression of nutrient release.

Enzymatic responses represent a distinct component of microbial P acquisition. Extracellular phosphatases, phytases, and C-P lyases are associated mainly with the transformation of organic P compounds [22]. Zhou et al. [82] demonstrated substrate-dependent mineralization of dissolved organic P by the bacterial alkaline phosphatase PhoA, while Wang et al. [79] associated phoD-harboring communities with alkaline-phosphatase activity and soil organic-P transformations. Acid-phosphatase production was also measured during cultivation of *Bacillus haynesii* ACP1 with phosphate rock [75]. In this system, phosphatase activity occurred together with organic-acid production and other changes in the culture medium. The enzyme measurement can therefore be interpreted as evidence of a bacterial P-acquisition response, while soluble-P and mineral-surface measurements provide information on reactions involving the inorganic phosphate source. In soils, assessing enzyme activity together with organic- and inorganic-P fractions may help determine the contribution of these parallel processes to P availability.

Siderophore production provides information on ligand-mediated interactions with Fe-containing phases. This trait was reported for phosphate-solubilizing *Bacillus* isolates [70] and for bacterial strains presenting P- and K-solubilization phenotypes [12]. Van den Berghe et al. [83] showed that siderophore synthesis and uptake promoted Fe acquisition from olivine and affected microbial interaction with the silicate mineral. In phosphate-bearing systems, siderophores may contribute to reactions involving Fe associated with mineral surfaces or Fe-P phases. Measurements of siderophore concentration, Fe speciation or complexation, soluble P, and surface composition can connect this bacterial trait to the transformation of Fe-bearing mineral phases. This approach may be particularly informative for polymineralic materials containing Fe-bearing minerals adjacent to nutrient-bearing phases.

K mobilization can involve different reactions according to the structural position of K in the mineral. K located at exposed or exchangeable sites may participate in ion exchange, while K retained within mica interlayers or the tetrahedral structure of feldspars may become accessible during progressive surface alteration [40,84]. Proton-associated reactions, organic ligands, cell attachment, extracellular compounds, and mineral-surface changes have been examined in bacterial systems containing K-bearing silicates [10,15,17,76]. The microscale observations reported by Han et al. [80] further indicate that bacterial colonization can connect localized feldspar dissolution with K availability to attached and neighboring cells. These studies provide a basis for examining K mobilization as an interface process involving mineral structure, surface accessibility, and bacterial establishment rather than only a change in final medium pH.

The composition of the cultivation medium can participate in the reactions associated with K release. Assimilation of NH_4_^+^ may be accompanied by proton release, while NH_4_^+^ can interact with accessible exchange sites in K-bearing minerals [76,84]. In media containing ammonium, changes in soluble K may consequently reflect the combined effects of bacterial metabolism and mineral-solution reactions. Comparison with an uninoculated treatment containing the same mineral and nitrogen source allows the abiotic contribution to be estimated, while a mineral-free inoculated treatment provides information on metabolic changes in the medium. These controls support attribution of the additional K detected in the bacterial treatment to processes associated with bacterial-mineral interaction.

Cellular acquisition and molecular responses provide complementary evidence of how bacteria respond to nutrients released or remaining limited during mineral interaction. The pho system includes genes associated with phosphate transport and the utilization of alternative P sources [85], while kdpFABC participates in cellular K acquisition under K limitation [15,17]. Expression of these systems can indicate the physiological state of the cells during mineral exposure. When evaluated together with bacterial biomass, cellular nutrient content, dissolved P or K, and mineral alteration, molecular measurements can help distinguish nutrient release into the medium from subsequent microbial acquisition. This integrated approach also provides a means of examining whether gene-expression responses coincide temporally with mineral dissolution and nutrient accumulation.

**Figure 3:**
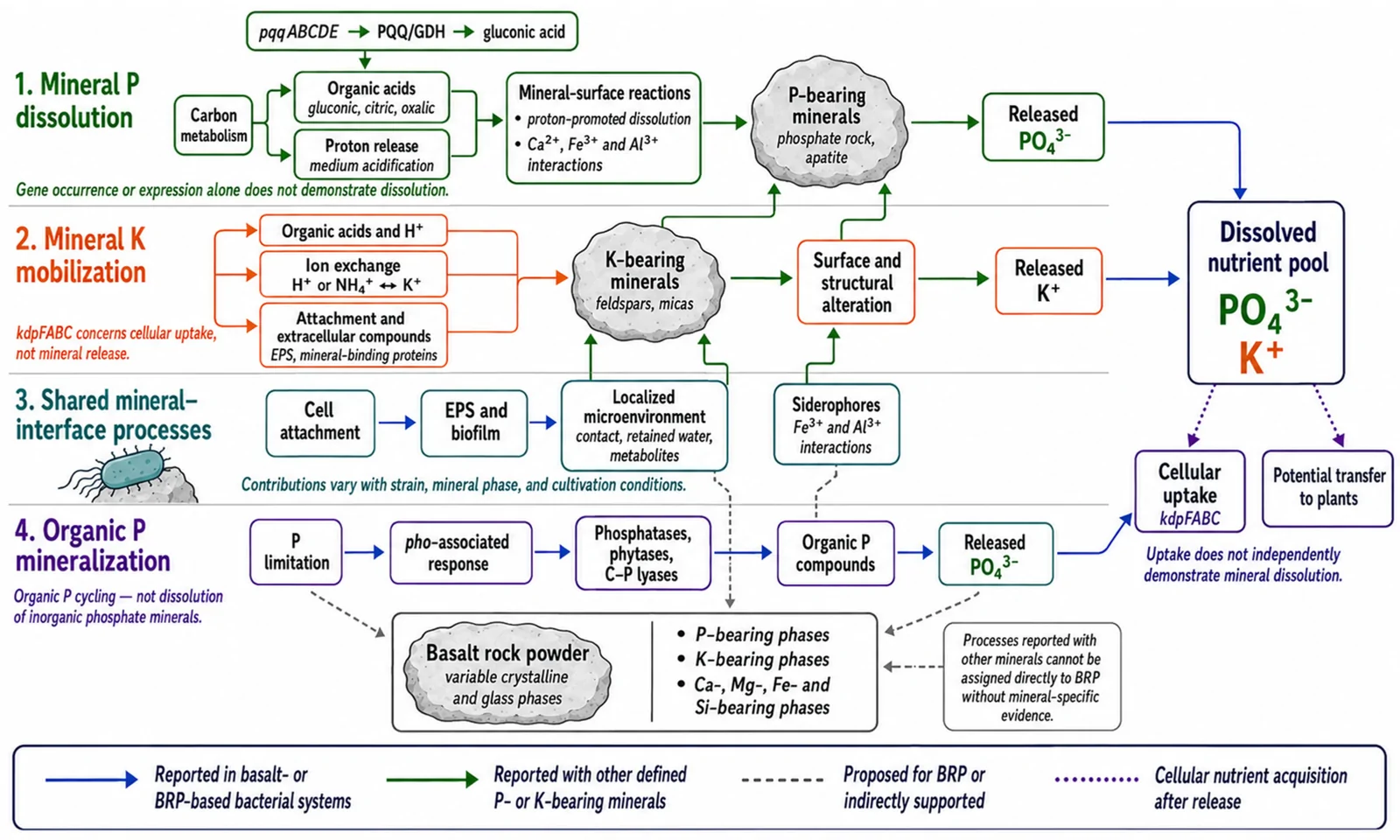
A conceptual representation of the bacterial processes associated with P and K mobilization from mineral sources, classified according to the available experimental evidence.

Evidence involving basalt extends these processes from isolated mineral phases to a polymineralic material. Byloos et al. [86] examined bacterial growth and elemental release from basalts of different compositions using *Cupriavidus metallidurans* CH34. More recently, Zhang et al. [87] combined elemental-release measurements with SEM-EDS, XRF, XRD, FTIR, organic-acid, siderophore, and biofilm analyses during basalt weathering by *Peribacillus simplex*. Although Fe and Si were the principal elements examined, this study illustrates how metabolic, solution, and mineralogical measurements can be integrated when the substrate contains several reactive phases. For BRP, similar analytical combinations may help relate microbial responses to the abundance, accessibility, and alteration of apatite, feldspars, micas, and associated minerals. Measurements of P and K release alongside changes in other elements may also assist in identifying which phases participate in the observed response. These metabolic, molecular, and mineral-surface processes, together with the experimental evidence used to examine them, are summarized in Figure 3.

### 4.3. Cultivation Conditions and Selection of Bacterial Strains

Cultivation media used for bacterial selection differ in nutrient composition, mineral substrate, and analytical purpose. NBRIP- and Pikovskaya-type media were used mainly to identify P-solubilization phenotypes [12,14,69,72,75,88], whereas Aleksandrov and modified mineral media were applied to evaluate K release from feldspar [12,13,15,16,17]. The measured response may therefore reflect characteristics of the bacterial strain and the chemical reactivity of the mineral included in the medium. Ateş [89] evaluated 118 bacterial isolates using the same NBRIP formulation and incubation period and reported mean soluble-P concentrations of 57.87 mg L^−1^ with phosphate rock and 421 mg L^−1^ with tricalcium phosphate. This internally controlled comparison indicates that the mineral selected for screening can affect the magnitude of P release and the apparent performance of the evaluated isolates. Conventional substrates may be used for initial phenotype detection, followed by evaluation of the selected strains with phosphate rock, feldspar, BRP, or another mineral considered for subsequent application.

The amount and form of P supplied during cultivation may also influence bacterial growth and the responses associated with P acquisition. An insoluble phosphate used as the principal P source allows soluble-P accumulation to be examined during bacterial interaction with the mineral [14,71,72]. Under these conditions, limited biomass formation may reflect restricted P acquisition during the evaluated period rather than the absence of interaction with the substrate. Liu et al. [85] cultivated *Burkholderia multivorans* WS-FJ9 with 0, 0.5, 1, 5, 10, and 20 mM soluble phosphate and performed transcriptomic analyses at 0, 5, and 20 mM. Increasing soluble-P concentrations were associated with greater bacterial growth but lower mineral-phosphate-solubilization activity and pyruvic acid production. Genes encoding glycerate kinase, 2-oxoglutarate dehydrogenase, and a sugar ABC-type transporter were progressively downregulated, relating P availability to changes in glucose metabolism [85]. *Paenibacillus sonchi* SBR5 also presented physiological and gene-expression responses during cultivation with hydroxyapatite [14]. These results indicate that bacterial growth and mineral-P release may respond differently to P availability. Their simultaneous measurement may help distinguish limited establishment under P restriction from a nutrient-mobilization response expressed during interaction with the mineral.

Treatments containing an insoluble-P source, a soluble-P growth source, and an uninoculated mineral control provide different reference conditions. The insoluble-P treatment describes bacterial growth and soluble-P accumulation during mineral exposure, while the soluble-P treatment indicates growth when acquisition from the mineral is not required [14,85]. The uninoculated treatment estimates P transferred to the liquid phase through reactions involving the mineral and culture medium [14,71,72]. A mineral-free inoculated treatment may additionally characterize changes in biomass, pH, and extracellular compounds occurring without the solid substrate. Comparison among these treatments can distinguish bacterial growth under P restriction, abiotic release, metabolic responses occurring without mineral exposure, and responses associated with bacterial–mineral interaction.

K-bearing systems may be examined using feldspar or other silicate minerals as the principal added K source [12,13,15,16,17]. A soluble-K treatment can provide a reference for bacterial growth when acquisition from the mineral is not required. Soluble-K measurements represent the fraction remaining in the liquid phase, whereas biomass and cellular-K measurements may provide complementary information on microbial acquisition [76,84]. Xue et al. [90] included an uninoculated feldspar treatment, allowing the soluble K measured after cultivation of strain JX-20 to be considered in relation to mineral-medium reactions occurring during the same period. Mineral-free inoculated treatments may additionally characterize biomass formation, medium acidification, and extracellular responses in the absence of the mineral. Selection of these controls can therefore be guided by whether the experiment is intended to examine bacterial establishment, abiotic K release, cellular acquisition, mineral alteration, or a combination of these responses.

Carbon-source composition is particularly relevant to assays in which nutrient mobilization is associated with acidification and extracellular metabolites. Glucose was included in several P- and K-mobilization media [14,15,71,72] and can provide carbon for biomass formation and gluconate production through the GDH-PQQ pathway [78]. Sánchez-González et al. [91] reported that *Bacillus pumilus* produced 246 mg P L^−1^ after four days when cultivated with sucrose and Ca_3_(PO_4_)_2_, whereas cultivation with glucose and FePO_4_ resulted in 282 mg P L^−1^ after six days. These responses were obtained with different phosphate compounds and sampling times, preventing attribution of the difference exclusively to the carbon source. They nevertheless indicate that the carbon source associated with soluble-P accumulation may depend on the chemical form of the supplied phosphate and the cultivation period. In mineral microcosms, Brucker et al. [92] associated organic-carbon availability with microbial P and Si release from apatite and weathered rock. Picard et al. [93] reported that carbon source and medium-buffering capacity influenced bacterial mineral weathering, while Blanco Nouche et al. [94] related biotite particle size to dissolution and bacterial siderophore responses. Evaluation of carbon sources should therefore consider their interaction with the mineral substrate, nutrient availability, and cultivation period, particularly when selecting conditions intended to represent the variable carbon supply encountered in the rhizosphere.

Temperature, agitation, inoculum concentration, mineral loading, particle size, and incubation time varied among the bacterial studies. Several mesophilic strains were cultivated at approximately 28–30 °C and 140–200 rpm [12,13,14,15,16,17,71], whereas *Bacillus haynesii* ACP1 was evaluated at 50 °C and 200 rpm following optimization for that strain [75]. Xue et al. [90] examined K release from feldspar by strain JX-20 under different cultivation conditions. The greatest soluble-K concentrations and reported feldspar-corrosion efficiencies were obtained at 28–30 °C, an initial pH of 7.4–8.0, 170 rpm, an inoculum volume of 20%, a feldspar concentration of 2 g L^−1^, a particle-size range of 0.02–0.03 mm, and 0.4 g L^−1^ ammonium sulfate. Values above or below these conditions were generally associated with lower soluble-K concentrations or corrosion efficiencies. Agitation at 170 rpm produced a greater response than static cultivation and the other shaking speeds evaluated, while increasing the feldspar concentration above 2 g L^−1^ did not result in a corresponding increase in corrosion efficiency. Mineral loading and inoculum concentration were therefore associated with non-proportional changes in the measured response. The variables were evaluated through single-factor experiments, preventing assessment of their interactions within the cultivation system.

Multivariable designs provide an alternative for examining such interactions. Barin et al. [95] applied response-surface methodology to evaluate vermicompost, phosphate rock, and sulfur during P solubilization by *Pseudomonas fluorescens* Ur21. The resulting model identified linear effects of vermicompost and sulfur, a quadratic effect of phosphate-rock concentration, and an interaction between vermicompost and phosphate rock. Although this formulation-based system differs from liquid bacterial-mineral cultivation, it illustrates how a response obtained with a mineral source may depend on interactions among experimental components rather than on their individual concentrations alone. Factorial or response-surface designs may therefore assist in examining interactions among mineral loading, inoculum concentration, carbon supply, pH, and other cultivation variables in BRP-based systems.

Particle size may influence both mineral dissolution and the bacterial responses expressed during cultivation. Blanco Nouche et al. [94] evaluated biotite fractions of <20, 20–50, 50–100, 100–200, and 200–500 µm using wild-type *Caballeronia mineralivorans* PML1(12) and a mutant unable to produce the siderophore rhizobactin. Biotite dissolution was assessed through released Fe and Al, while bacterial growth, pH, and siderophore production were examined as physiological responses. After seven days, the wild-type strain was associated with greater Fe release from particles smaller than 100 µm than from the larger fractions. Siderophore production was detected early during interaction with the smaller particles and subsequently decreased as dissolved Fe accumulated. The absence of the same particle-size response in the rhizobactin-deficient mutant related the effect of particle size to bacterial Fe-acquisition activity rather than only to differences in exposed mineral surface. Particle size can therefore be examined as both a physical property of the mineral and an experimental factor associated with microbial responses during mineral interaction.

Incubation time determines the period of bacterial–mineral interaction represented by the analytical result. In the *Pseudomonas-Serratia* coculture, soluble P increased from 58.1 mg L^−1^ after 8 h to 88.1 mg L^−1^ after 12 h [71]. During cultivation of *Priestia megaterium* NK851 with feldspar, soluble K increased from 157 mg L^−1^ after three days to 222 mg L^−1^ after five days [17]. Xue et al. [90] observed a rapid increase in soluble K during the first eight days of cultivation with strain JX-20, followed by a smaller change between days eight and ten and little additional accumulation after ten days. These temporal responses indicate that a single sampling time may represent an early accumulation period, a period of slower release, or a condition approaching a stable soluble concentration. In the time-series experiment of Xue et al. [90], samples were centrifuged, filtered, and analyzed by atomic absorption spectroscopy using the same procedure throughout cultivation. Consistent phase separation and analytical procedures are particularly relevant when interpreting temporal changes, as differences in the analyzed fraction could otherwise be interpreted as changes in nutrient mobilization. Measurements of bacterial growth, pH, extracellular compounds, and soluble nutrients at corresponding sampling times may relate nutrient accumulation to the biological and chemical responses occurring during cultivation [14,17,71].

P- and K-specific media provide information on separate bacterial traits. Bouizgarne et al. [12], for example, evaluated P solubilization using tricalcium phosphate in NBRIP medium and K solubilization using feldspar in Aleksandrov medium. The study identified both phenotypes, but each response was expressed under a different medium composition and mineral source. This experimental separation is useful for initial characterization, although it does not indicate whether the two responses occur concurrently under a common cultivation condition. For bacteria considered for BRP-based systems, subsequent cultivation with a mineralogically characterized material may examine whether soluble P and K change during the same period. Concurrent measurements of biomass, pH, extracellular compounds, and mineral-surface alteration may indicate whether responses involving both elements are associated with common cultivation conditions or with distinct periods of bacterial–mineral interaction. This proposed evaluation extends the separate screening approach used by Bouizgarne et al. [12] to a polymineralic material containing P- and K-bearing phases.

The studies included in the analysis indicate that conventional phenotype screening can be followed by evaluation with the mineral material considered for the intended application. A recent complementary study also examined the separation of biomass production and mineral exposure. Ahmad et al. [96] compared one- and two-stage cultivation during the biosolubilization of low-grade phosphate rock by acidophilic bacteria. In the two-stage process, bacterial growth and acid production preceded the addition of phosphate rock, and the resulting P release was greater than that obtained when the microorganisms and mineral were introduced simultaneously. The configuration illustrates how the time of mineral addition may influence the relationship among biomass formation, metabolite accumulation, and mineral dissolution. Its application to heterotrophic bacteria or BRP would require evaluation under the corresponding physiological and mineralogical conditions. Single- and two-stage cultivation may be compared through viable biomass, soluble P and K, extracellular compounds, and mineral alteration. Selection of either configuration would remain dependent on the bacterial strain, BRP composition and concentration, measured response, and intended use of the culture or formulation.

### 4.4. Translation to Soil and Agronomic Performance

Culture-based assays characterize bacterial–mineral interactions under controlled conditions of temperature, carbon supply, agitation, mineral concentration, and contact between cells and mineral particles [12,13,14,15,16,17,71,75]. In soil, the expression of these interactions may also depend on pH buffering, nutrient sorption and precipitation, water distribution, root activity, and interactions with resident microbial communities, as discussed by Pang et al. [97]. Wang et al. [98] examined the influence of soil chemistry through a 70-day incubation using five P sources in an acidic red soil and a slightly alkaline fluvo-aquic soil. Olsen-P was measured throughout incubation, and P was separated into labile, moderately labile, sparingly labile, and non-labile fractions after 70 days. Differences between soils and P sources indicated that the P detected after soil application depends on both the applied material and its subsequent distribution among soil fractions. Soil and plant experiments extend culture-based measurements by examining whether bacterial treatments are associated with changes in extractable nutrient pools, plant nutrient accumulation, biomass, or yield.

Phosphorus released from a mineral source may remain in solution or undergo adsorption, precipitation, and redistribution among soil fractions according to pH, mineral surfaces, and the presence of Ca-, Fe-, and Al-bearing phases [97,98]. Soil K occurs in solution, exchangeable, non-exchangeable, and mineral pools, whose relative contributions vary with soil mineralogy, exchange properties, and nutrient removal by crops [42,43]. Soil fractionation and plant nutrient analysis consequently represent distinct portions of nutrient distribution. Changes in labile soil P or exchangeable K describe operationally defined soil pools, whereas P or K accumulation in plant tissues indicates nutrient acquisition during cultivation [99,100]. Agreement between these measurements can associate the microbial treatment with nutrient availability and plant acquisition even when the mobilized nutrient does not accumulate in the soil-solution fraction.

The P-related studies in Table 3 differed in the connection established between bacterial screening, mineral application, and plant response. Afzal et al. [73] applied *Bacillus velezensis* to wheat alone, with rock phosphate, and with different proportions of single superphosphate and urea. The combination of *B. velezensis* and rock phosphate was associated with increases in soil phosphate and leaf P, accompanied by changes in wheat growth and yield. As rock phosphate remained present during cultivation, the correspondence between soil P, leaf P, and plant response is relevant to the agronomic interpretation of the mineral-bacterium treatment. However, the strain also produced IAA and organic acids and presented other plant-growth-related characteristics. The plant responses may consequently reflect mineral-P mobilization alongside additional bacterial effects.

*Bacillus megaterium* Ag87 and *Lysinibacillus* sp. Ag94 represent a different experimental sequence [69]. The strains were selected through phosphate-solubilization and plant-growth-related assays and were subsequently applied to maize seeds in greenhouse and field experiments. In the field, inoculated seeds received 30% of the recommended phosphate fertilizer rate, but the Fe phosphate used during laboratory screening was not applied. At four sites during the first harvest, Ag94 and the Ag87-Ag94 treatment produced mean yield increases of 16.3% and 21.45%, respectively, relative to the treatment receiving the same P rate without inoculation [69]. Responses varied among sites, and the combined treatment did not differ statistically from full P fertilization in that experimental series. The study indicates that the selected strains were associated with maize yield and P-use efficiency under reduced soluble-P fertilization. It does not establish that the phosphate-solubilization response measured with Fe phosphate was maintained with the same mineral under field conditions.

This distinction is important when interpreting bacterial application strategies. Seed inoculation, as used for Ag87 and Ag94 [69], places the bacteria near the developing root but does not ensure direct contact with an applied mineral particle. Application of bacteria with rock phosphate, as examined by Afzal et al. [73], creates a closer experimental relationship among the microorganism, mineral source, soil, and plant. These approaches address different questions. The first evaluates whether strains selected for P-related characteristics contribute to crop performance under a defined fertilization rate. The second examines plant and soil responses when the bacterial inoculant and low-solubility mineral source are applied within the same treatment. Direct assessment of microbial-mineral mobilization requires the mineral-containing inoculated treatment to be compared with the corresponding uninoculated mineral treatment.

The K-related studies also separated culture-based selection from agronomic application. *Pseudomonas aeruginosa* A10 presented K mobilization in culture and was subsequently applied to cotton cultivated in pots containing saline-sodic soil [13]. Inoculation was associated with increases in plant height, root development, and shoot and root biomass. The soil experiment did not include the characterized K-bearing mineral used during screening, and the contribution of K released from native soil minerals was not separated from other bacterial effects. The results indicate that A10 remained associated with plant growth under alkaline and salt-affected conditions, but they represent soil and plant evidence rather than direct measurement of mineral-K mobilization.

*Paenibacillus mucilaginosus* JGK was applied to two-month-old apple seedlings by irrigation with 50 mL of bacterial suspension containing 1 × 10^9^ CFU mL^−1^ [74]. The treatment was associated with seedling growth and plant K accumulation, while the population of JGK decreased from 4.0 × 10^9^ to 1.5 × 10^9^ CFU g^−1^ of soil over 28 days. Rhizosphere-available K did not increase significantly. The absence of K accumulation in the extractable soil fraction, despite greater plant K accumulation, may reflect plant acquisition during the cultivation period, changes in K distribution not represented by the selected extraction, or contributions from improved root development. The detection of organic acids and phytohormones in JGK metabolites indicates that K-related and plant-growth-related responses may have occurred concurrently [74]. Repeated measurements of soil K fractions and plant K accumulation would distinguish transient changes in soil availability from nutrient acquisition over time.

The application methods used in these K studies also affect their interpretation. A10 was introduced into saline–sodic soil after culture-based selection [13], whereas JGK was applied as a concentrated bacterial suspension directly to the seedling soil [74]. Neither soil experiment included a defined K-bearing mineral amendment. These studies are relevant to bacterial establishment and plant K acquisition from existing soil pools, but they do not quantify K transferred from an externally applied feldspar or BRP. Experiments combining the inoculant with a characterized mineral source would allow changes in native soil K to be distinguished from the contribution of the amendment.

Closer connections among a low-solubility P source, soil transformations, and crop response were reported by Torres-Cuesta et al. [99] and Biswas et al. [100]. Torres-Cuesta et al. [99] conducted an 18-month field experiment in a Kikuyu-grass pasture using rock phosphate, soluble P fertilizer, and an unfertilized treatment, evaluated with and without a consortium comprising *Azospirillum brasilense*, *Rhizobium leguminosarum*, and *Herbaspirillum* sp. Rock phosphate combined with bacterial inoculation was associated with greater labile soil P, acid phosphomonoesterase activity, dry-matter accumulation, and pasture quality. The relationship between soil inorganic P and grass productivity connects changes in soil P availability with an agronomic response. Acid phosphomonoesterase activity concerns organic-P transformations, however, and should not be interpreted independently as evidence of rock-phosphate dissolution.

Biswas et al. [100] evaluated different quantities of phosphate-solubilizing bacteria inoculated onto low-grade rock phosphate through soil-incubation and wheat pot experiments in a subtropical Inceptisol. The treatments were compared with a commercial P fertilizer, and the analyses included P adsorption and fixation, soil P fractions, phosphatase activities, plant P acquisition, and wheat growth. Bacterial inoculation was associated with lower P fixation, changes in labile P fractions, and greater plant P acquisition. The inoculated rock phosphate was evaluated as a partial supplement to soluble P fertilizer. In this experimental structure, P adsorption and fractionation described soil transformations, phosphatase activities described biological responses, and plant P and biomass represented acquisition and agronomic expression. Correspondence among these endpoints strengthens the interpretation of the treatment response, although the phosphatase measurements do not specifically quantify dissolution of the inorganic mineral phase.

Soil chemistry contributed differently to the responses observed in these experiments. The Andisol evaluated by Torres-Cuesta et al. [99] was derived from volcanic materials and presented substantial P-retention capacity. Changes in labile P were therefore relevant to determining whether rock-phosphate application and inoculation altered the fraction of P represented by soil extractions. Biswas et al. [100] measured P adsorption, fixation, and fractionation directly in the Inceptisol, allowing the inoculated rock-phosphate treatment to be interpreted according to the reactions observed in that soil rather than from soil classification alone. The saline–sodic soil used for cotton presented an alkaline and ionically distinct environment in which K-Na relationships, bacterial activity, and root growth may all contribute to the plant response [13].

Wang et al. [98] further examined soil-dependent P transformations by applying equal quantities of P from different sources to an acidic red clay soil at pH 5.7 and a slightly alkaline fluvo-aquic silt-loam soil at pH 7.9. Superphosphate and manure-derived P produced smaller increases in Olsen-P in the acidic red soil, where a greater proportion was represented in less labile fractions. In contrast, bone meal, which contained apatite as its principal P-bearing phase, presented a greater available-P response in the acidic soil than in the alkaline soil. These contrasting responses indicate that acidic conditions may favor dissolution of some apatite-bearing materials while Fe- and Al-rich surfaces retain part of the released P. The effect of soil pH on a mineral amendment should consequently be examined in relation to both the nutrient-bearing phase and the sorption characteristics of the soil. For BRP, this interaction is particularly relevant as P occurs within a polymineralic material rather than as a concentrated phosphate source.

Plant responses may also reflect bacterial characteristics other than P or K mobilization. Several strains presented IAA production, siderophore production, pathogen antagonism, or other plant-growth-related traits [12,69,70]. As these traits occurred in the same strains evaluated for nutrient mobilization, changes in roots, nutrient accumulation, biomass, or yield cannot be assigned exclusively to P or K release. This distinction is illustrated by the apple-seedling experiment, in which plant K accumulation increased without a significant increase in rhizosphere-available K [74]. An uninoculated mineral treatment can describe the response to the amendment, an inoculated treatment without the mineral can describe the response associated with the bacterial strain, and an inoculated mineral treatment can examine their combined application. Soil nutrient fractions and plant P or K concentrations can then be interpreted alongside root traits, biomass, and yield.

The experimental scale also influenced the evidence obtained. The A10 and JGK studies were conducted in pots or controlled seedling systems [13,74], where moisture, inoculum placement, and exposure of roots to the applied bacteria could be maintained more consistently. Torres-Cuesta et al. [99] examined repeated pasture responses over 18 months, while Massucato et al. [69] evaluated inoculation across several field sites and reported variation among locations and growing periods. Field experiments incorporate differences in rainfall, temperature, soil properties, crop development, and resident microbial communities that are not represented in liquid culture or short-term pot assays. Responses obtained at one site or cultivation period consequently describe the evaluated combination of inoculant, soil, crop, fertilization, and environmental conditions rather than a fixed characteristic of the bacterial strain.

The reviewed agronomic studies present three complementary forms of evidence: changes in soil nutrient fractions, nutrient accumulation by plants, and crop growth or yield. Rock-phosphate studies connected these responses more directly when the same mineral source remained present during soil or plant evaluation [73,99,100]. The maize, cotton, and apple experiments primarily examined the agronomic performance of strains previously selected for P- or K-related characteristics [13,69,74]. For BRP, maintaining the same characterized material across culture, soil-incubation, and plant experiments may allow soluble nutrient measurements to be related to soil P and K fractions, plant acquisition, and agronomic response. Evaluation under soils differing in acidity, clay and oxide contents, cation-exchange capacity, initial nutrient pools, and nutrient-retention properties may indicate which bacterial-BRP responses remain represented after transfer from controlled cultivation to soil. Similar distinctions among nutrient release, soil availability, and plant acquisition are considered in the following sections for photosynthetic microorganisms and mixed microbial systems.

## 5. Microalgae and Cyanobacteria Roles in Phosphorus and Potassium Mobilization

Studies examining photosynthetic microorganisms cultivated with mineral nutrient sources comprise different experimental approaches. Within the analytical dataset, available P or dissolved K was measured mainly in cyanobacterial cultures containing phosphate rock, tricalcium phosphate, basalt, or rhyolite [101,102,103]. Studies with eukaryotic microalgae examined growth and biomass production when struvite replaced soluble P, N, and Mg salts [104,105]. These approaches describe different components of mineral utilization. Changes in the dissolved fraction indicate nutrient transfer from the solid material to the culture solution, whereas growth, N fixation, lipid production, protein production, and cellular nutrient accumulation describe physiological responses after the nutrient becomes available. Mineral release and nutrient acquisition are therefore related but analytically distinct processes [104,105,106,107].

Table 4 organizes the included studies according to microorganism, mineral source, cultivation conditions, measured response, and type of evidence. Comparisons conducted within the same experimental design are particularly informative for examining effects associated with microbial strain, mineral composition, mineral concentration, and incubation period. Comparisons among studies require consideration of the analytical fraction, sampling time, calculation basis, and presence of abiotic controls.

### 5.1. Experimental Patterns for Phosphorus Mobilization

Culture-based P mobilization was examined mainly with Mussorie rock phosphate and tricalcium phosphate in N-free BG-11 [102,103]. Soluble K_2_HPO_4_ was replaced by the mineral P source, while KCl was supplied separately to maintain K availability [102]. This medium formulation allowed the response to P source to be examined without introducing simultaneous K restriction. Cultures were maintained at 28 ± 2 °C under a 16:8 h light-dark cycle, using P-starved inocula and mineral concentrations corresponding to 10, 20, or 30 mg P L^−1^. These conditions permitted available P, growth, and N fixation to be evaluated during dependence on the supplied mineral P.

*Westiellopsis prolifica* and *Anabaena variabilis* presented their highest reported available-P concentrations on day 7, followed by changes during the remaining cultivation period [102]. At 30 mg P L^−1^, *W. prolifica* presented 0.32 µg P mL^−1^ with rock phosphate and 0.38 µg P mL^−1^ with tricalcium phosphate, while the corresponding values for *A. variabilis* were 0.35 and 0.37 µg P mL^−1^. The similar concentrations obtained with the two substrates at this sampling time indicate that both cyanobacteria interacted with natural rock phosphate and synthetic calcium phosphate under the evaluated conditions. Variation after day 7 also indicates that the dissolved fraction represented a balance among continued release, cellular acquisition, adsorption, and possible secondary reactions rather than cumulative dissolution alone.

The comparison among five diazotrophic cyanobacteria extended this analysis by examining available P together with growth and N fixation [103]. *A. variabilis* presented 0.224 µg P mL^−1^ when tricalcium phosphate was supplied at 20 mg P L^−1^, although biomass formation and N fixation differed among strains, mineral sources, concentrations, and sampling times. Available P described the fraction measured in the culture solution, whereas growth and N fixation indicated whether the nutrient supplied under the same conditions was associated with physiological activity. A strain presenting detectable P release but limited growth may differ functionally from one maintaining biomass formation and N fixation at a similar dissolved-P concentration. Evaluation of these responses within the same assay may therefore assist strain selection according to mineral interaction and physiological establishment.

Complementary experiments with the same cyanobacterial models indicate that mineral composition also affected the physiological response. Yandigeri et al. [108] reported differences in growth and N fixation when *A. variabilis* and *W. prolifica* were cultivated with rock phosphate or tricalcium phosphate. A subsequent investigation related mineral-phosphate solubilization to N-fixation activity in diazotrophic cyanobacteria [109]. These findings indicate that P mobilization and N fixation may respond concurrently to the supplied mineral, although the two variables do not necessarily present proportional changes. Mineral-P assays involving diazotrophic strains can therefore examine whether P release is accompanied by maintenance of N-fixation activity rather than considering available P as the only selection criterion.

Evidence obtained with other phosphate phases extends the mineral range represented by Table 4. Cameron and Julian [110] reported cyanobacterial growth with hydroxyapatite as the sole source of P and Ca, indicating that phototrophic utilization of mineral P is not restricted to rock phosphate or tricalcium phosphate. He et al. [111] cultivated *Chlorella vulgaris* with Fe-phosphate sediment at an initial pH of 7 and calculated Fe-P release ranging from 0.055 to 0.45 mg d^−1^ in 200 mL. Released Fe was associated with cellular flocculation, while P removal remained below 30% during the release period. The latter experiment shows that mineral-P release may alter the culture through the simultaneous release of associated cations. This interaction is pertinent to BRP, in which P-bearing phases coexist with Fe-, Ca-, Mg-, and silicate minerals and changes in one element may influence aggregation, growth, or nutrient acquisition.

The response reported by Yandigeri et al. [102] differed from the acidification pattern frequently described for bacterial phosphate solubilization. A decrease in bulk-medium pH was not detected during cultivation with rock phosphate or tricalcium phosphate, while phthalic acid was identified among the extracellular compounds. Its occurrence with available P suggests a possible contribution of ligand-mediated reactions under the evaluated BG-11 conditions, although the contribution of this compound was not quantified separately. The result indicates that cyanobacterial mineral-P mobilization may occur without generalized acidification of the culture. Evaluation of phototrophic strains may therefore include extracellular ligands and time-resolved pH measurements rather than relying exclusively on final bulk pH.

Organic-P utilization represents another component of phototrophic P acquisition. Afkairin et al. [112] examined an *Anabaena*-based cyanobacterial treatment with bone meal and another organic P source and reported P solubilization together with phosphatase activity. This response concerns enzymatic transformation of organic P and differs from dissolution of apatite or other inorganic phosphate phases. Reviews of cyanobacterial P physiology describe high-affinity transporters, phosphatases, phosphonate-utilization pathways, and intracellular reallocation as responses to limited phosphate availability [113]. Phosphatase activity measured during cultivation with BRP would consequently describe cellular P status or transformation of organic P present in the system unless accompanied by direct evidence of inorganic mineral dissolution.

A similar distinction applies to eukaryotic microalgae. In *C. vulgaris*, phosphatase expression varied with intracellular P status and external pH [106]. Bossa et al. [18] describe phosphatases, high-affinity phosphate transport, and polyphosphate accumulation as components of P acquisition and storage in microalgae, while Solovchenko et al. [114] discuss the transfer of dissolved P into intracellular pools. These responses occur after P enters an accessible fraction and do not independently identify its mineral source. Their combined evaluation with dissolved inorganic P and residual-mineral characterization may indicate whether physiological P acquisition accompanies nutrient transfer from a solid substrate.

The struvite studies examined nutrient utilization under a different mineralogical context. Moed et al. [104] replaced soluble P, N, and Mg salts in BBM with synthetic struvite while supplying K separately. *C. vulgaris* was cultivated mainly for seven days under continuous illumination with air or 10% CO_2_, and growth and lipid production were evaluated. The maintenance of growth indicated that struvite-derived nutrients became available during cultivation. Gas composition formed part of the chemical context in which mineral nutrients were utilized, as CO_2_ supply can affect culture pH and struvite solubility.

Muys et al. [105] cultivated *C. vulgaris* and *Limnospira* sp. PCC 8005 using recovered struvite as the P source. Growth and protein production were comparable to or greater than those obtained with conventional phosphate media. Physicochemical experiments conducted separately from the cultures produced struvite dissolution rates of 0.32–4.7 g P L^−1^ d^−1^, depending on pH, temperature, mineral surface area, and ion concentrations. Separation of the dissolution and cultivation experiments allowed mineral solubility and microbial nutrient utilization to be interpreted independently. Although struvite and BRP differ in composition, this experimental structure is applicable to BRP investigation: abiotic assays can describe P and K release under defined chemical conditions, while subsequent cultivation can examine the distribution of the available fraction between the solution and phototrophic biomass.

The P evidence therefore identifies three responses relevant to BRP investigation. Cyanobacteria can interact with calcium-associated phosphates under phototrophic cultivation [102,103], eukaryotic microalgae may influence P release from Fe-associated material [111], and both groups present physiological mechanisms for acquiring and storing P after its release [18,106,107,113]. These responses indicate that evaluation of BRP may combine mineral-P release, associated-cation behavior, physiological P status, and cellular P accumulation within the same cultivation period.

### 5.2. Evidence for Potassium Mobilization

The K evidence included in Table 4 derives from one study in which six cyanobacterial strains were cultivated with crystalline basalt and rhyolite [101]. The strain-specific entries therefore represent comparisons within a common experimental design. Maintaining the same mineral loading, temperature, light regime, initial cell concentration, and incubation period allowed differences associated with the cyanobacterium and rock substrate to be examined with lower variation from cultivation conditions.

Basalt and rhyolite were supplied at 20 g per culture as particles smaller than 1 mm in sterile double-distilled water containing 10 mM NaNO_2_. Cultures were maintained statically at 25 °C under a 16:8 h light-dark cycle, with an initial concentration of approximately (10^9^) cells mL^−1^, for 45 days [101]. *Anabaena cylindrica* PCC 6309 presented the highest final dissolved-K concentration with basalt, reaching 135.32 µmol L^−1^. The other strains presented values ranging from 41.70 to 65.89 µmol L^−1^. *Gloeocapsa* sp. OU_20 grew with basalt but not with rhyolite, indicating that rock composition was associated with both microbial establishment and the dissolved-element response.

Comparison with abiotic treatments showed that the microbial contribution depended on rock type [101]. Cyanobacterial growth increased the linear release rates of Ca, Mg, Si, and K from basalt by 5.21–12.48-fold in the *A. cylindrica* treatment, while the corresponding increases with rhyolite ranged from 2.04- to 2.97-fold. Release rates obtained with basalt were at least ninefold greater than those obtained with rhyolite for this strain. The pH increased by 1.9 units with basalt and 0.83 units with rhyolite. Thus, faster elemental release from basalt occurred together with increasing bulk pH, indicating that the response was not associated with generalized acidification. The authors related the different responses to rock composition, including the greater abundance of readily weathered phases and bioessential elements in basalt.

The strain comparison also indicates that growth and elemental release were connected to the nutrient composition of the rock. Basalt supplied greater concentrations of Ca, Fe, Mg, and other elements than rhyolite, while rhyolite contained a greater proportion of quartz and presented lower biomass formation [101]. Mineral composition may therefore influence K mobilization indirectly through its effects on phototrophic establishment and directly through the reactivity of its K-bearing and associated phases. For BRP, strain selection may consequently consider biomass maintenance and multielement release rather than soluble K alone.

Complementary studies of phototrophic-silicate weathering describe processes relevant to interpretation of the basalt results, even when K was not the principal measured element. Bundeleva et al. [115] cultivated *Synechococcus* PCC 7942 with Mg-bearing silicates and related cyanobacterial activity to Mg release and subsequent carbonate precipitation. Lamérand et al. [116] similarly examined the temporal relationship among cyanobacterial growth, increasing pH, dissolved Mg and Si, and formation of secondary Mg-silicate phases. These studies indicate that biological enhancement of mineral dissolution can occur concurrently with removal of released elements through precipitation. A dissolved-element concentration measured at the end of cultivation may therefore be lower than the cumulative amount transferred from the original mineral.

Demirel-Floyd et al. [117] cultivated *Leptolyngbya glacialis* with mixed-lithology glaciofluvial sediments at 12 °C. Biotic Antarctic reactors presented approximately threefold greater Si-weathering rates than abiotic controls, whereas the difference was smaller for Icelandic sediments. Newly formed clay and Fe-(oxy)hydroxide phases occurred in association with biofilms on mafic minerals. The contrast indicates that cyanobacterial effects can depend on the initial weathering state and mineral assemblage. This finding is relevant to BRP materials that differ in glass content, proportions of crystalline phases, previous hydrothermal alteration, and occurrence of secondary minerals.

Evidence from freshwater mineral-colonizing communities also extends the range of phototrophic-silicate interactions. Mustoe [118] examined epilithic algae and cyanobacteria in contact with biotite and other Fe-bearing minerals and reported biological acquisition of mineral-derived Fe. Although the study did not quantify K mobilization, the use of biotite is pertinent to BRP, as Fe and K occur within the same mineral structure. Preferential acquisition of one element and alteration of the mineral surface may affect the subsequent distribution of the other without producing congruent release.

The additional studies do not expand the number of direct K measurements, but they clarify processes represented only partially by the final dissolved-K concentrations in Table 4. Phototrophic growth may alter pH, accelerate release of several elements, promote attachment to mafic surfaces, and contribute to secondary-phase formation. For BRP, simultaneous determination of K, Si, Al, Fe, Mg, and Ca in the liquid, biomass, and residual solid may indicate whether K accumulation is associated with alteration of feldspars, micas, glass, or secondary phases. This multielement interpretation is more informative than treating K concentration as an isolated measure of microbial efficiency.

Direct K evidence remains concentrated on cyanobacteria, while a comparable mineral-K experiment with a eukaryotic microalga was not identified in the analytical dataset. The available results consequently identify cyanobacterial strains and batch-cultivation designs suitable for examining basalt weathering. They do not establish a general difference between cyanobacteria and eukaryotic microalgae, as the latter group has not received equivalent experimental evaluation with K-bearing minerals.

### 5.3. Processes Associated with Phototrophic Mineral Interactions

Phototrophic interactions with mineral sources may involve changes in solution chemistry, extracellular ligands, EPS, attachment to mineral surfaces, ion binding, nutrient transport, and intracellular storage. These processes can occur concurrently and may produce different effects on the dissolved, cellular, and residual-solid fractions.

Bulk acidification does not represent a common pattern across the cyanobacterial mineral studies. Mineral-P mobilization by *W. prolifica* and *A. variabilis* occurred without a detected reduction in final bulk pH [102], while A. cylindrica increased pH and accelerated elemental release from basalt [101]. Photosynthetic CO_2_ assimilation may increase pH during illuminated periods, whereas respiration during dark periods, gas exchange, extracellular metabolites, and mineral reactions may produce different temporal or localized conditions. Measurements conducted throughout the light-dark cycle can therefore distinguish a stable final pH from transient changes associated with mineral release.

Complementary mineral studies reinforce this temporal interpretation. Bundeleva et al. [115] and Lamérand et al. [116] related cyanobacterial activity to increasing pH, silicate dissolution, and formation of Mg-bearing secondary phases. These results indicate that increasing pH may accompany dissolution of the original substrate while favoring subsequent precipitation of some released elements. In phototrophic BRP systems, dissolved P or K at one sampling time may consequently represent release from primary minerals after subtraction of cellular uptake, adsorption, and secondary mineral formation.

Extracellular ligands introduce reactions that may not be represented by bulk pH. Phthalic acid was detected during cultivation of *W. prolifica* and *A. variabilis* with mineral phosphate [102]. Carboxyl groups can interact with mineral-associated cations and may influence the chemical environment of phosphate-bearing surfaces. The occurrence of this compound with available P indicates a candidate ligand-mediated process, although its individual contribution was not separated experimentally. Targeted quantification of extracellular acids together with dissolved P, Ca, Fe, and Al could examine whether ligand production corresponds to release from specific phosphate associations in BRP.

EPS may influence phototrophic mineral interactions through particle attachment and ion binding. Babiak and Krzemińska [119] describe culture conditions associated with the quantity and composition of EPS produced by microalgae, including nutrient availability, irradiance, salinity, temperature, and growth phase. The resulting polymers contain functional groups capable of interacting with dissolved ions and solid surfaces. EPS production should therefore be treated as a strain- and cultivation-dependent response rather than an invariant characteristic of phototrophic cultures.

Experimental studies demonstrate that EPS can redistribute metal cations. Ciempiel et al. [120] characterized soluble EPS from *Parachlorella kessleri* and *C. vulgaris* and associated Pb and Cd sorption with carboxyl, hydroxyl, and carbonyl groups. Paper et al. [121] compared intact cyanobacterial biomass, EPS-depleted cells, and isolated EPS from *Komarekiella* sp., *Desmonostoc muscorum*, and *Nostoc* sp. Extracted EPS adsorbed 103.5–138.2 mg g^−1^ of the evaluated rare-earth elements and accounted for approximately 16–41% of adsorption by intact biomass. These metals differ from P and K, but the experiments demonstrate that extracellular material can retain mineral-derived cations outside the cell. In a BRP culture, EPS-associated Ca, Mg, Fe, or trace elements may consequently be absent from the filtered liquid fraction without being incorporated into cells or precipitated as a new mineral.

Attachment can also produce chemical conditions that differ from those of the bulk solution. Demirel-Floyd et al. [117] observed newly formed mineral phases associated with cyanobacterial biofilms on mafic particles. Stigliano et al. [122] used time-resolved interferometric imaging to examine *Chroococcidiopsis thermalis* PCC 7203 attached to calcite. Local surface responses depended on solution saturation and the residence time of attached cells, while the microscale pattern differed from the global dissolution response. Although calcite is not a P- or K-bearing phase, the study demonstrates that final pH and bulk elemental concentrations may not describe reactions occurring immediately beneath or around attached phototrophic cells.

Mineral release also needs to be distinguished from cellular acquisition. Caille et al. [113] describe high-affinity phosphate transporters, alkaline phosphatases, and changes in intracellular P allocation as responses to phosphate limitation in cyanobacteria. Bossa et al. [18] and Solovchenko et al. [114] discuss phosphate transport and polyphosphate accumulation in eukaryotic microalgae. These physiological responses determine how much released P remains in the solution and how much becomes stored in biomass. Increased cellular P can occur with limited dissolved-P accumulation when uptake proceeds rapidly after mineral release.

A similar analytical distinction applies to K. Once released into the liquid phase, K may remain dissolved or become incorporated into the cells. Unlike P, K is not stored as a polymer analogous to polyphosphate and remains associated primarily with ionic balance and cellular metabolism. Simultaneous measurement of dissolved K, total cellular K, and residual-solid K may therefore indicate whether a small change in the liquid fraction reflects limited mineral release or rapid biological acquisition.

**Figure 4:**
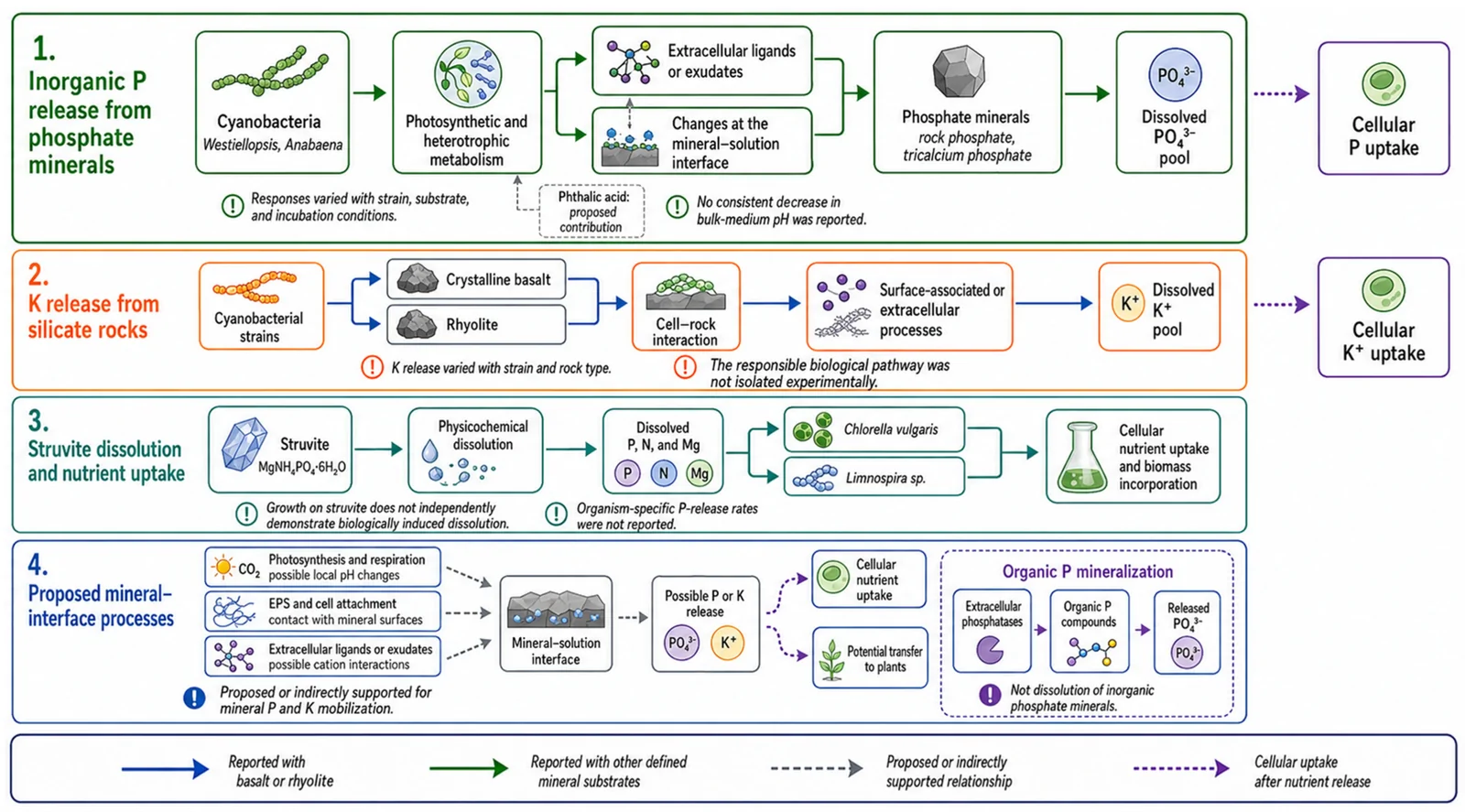
Conceptual model of P and K mobilization by microalgae.

Figure 4 summarizes these connected responses. Mineral alteration transfers elements from the solid into an operationally available fraction. Changes in pH, extracellular ligands, EPS, and cell attachment may influence the mineral–solution interface, while adsorption, cellular uptake, and secondary precipitation redistribute the released elements. The concentration measured in the liquid phase represents the net result of these processes rather than the total quantity removed from the original mineral.

### 5.4. Cultivation Conditions and Implications for BRP-Based Studies

Cultivation conditions define whether an experiment emphasizes biomass production, mineral interaction, nutrient release, or cellular acquisition. The phosphate experiments used N-free BG-11, P-starved inocula, 28 ± 2 °C, a 16:8 h light-dark cycle, and mineral concentrations corresponding to 10–30 mg P L^−1^ [102,103]. These conditions allowed strain and P-source effects to be examined while maintaining K through separate KCl addition. The occurrence of maximum available-P concentrations near day 7 indicates that early and intermediate sampling can identify responses that may no longer be represented at the end of a 35-day assay.

Previous P status is particularly relevant to this design. P-starved cells may express transporters, phosphatases, and other P-acquisition responses before exposure to the mineral [102,113]. Bossa et al. [18] and Solovchenko et al. [114] also describe changes in uptake and polyphosphate accumulation after transitions between P limitation and resupply. Inoculum preparation may therefore influence both the initial rate of nutrient acquisition and the dissolved concentration measured during cultivation. Reporting the duration of P starvation, initial biomass, cellular P content, and nutrients transferred with the inoculum would assist interpretation of mineral-release assays.

The basalt study used a different cultivation arrangement: washed cells at approximately (10^9^) cells mL^−1^, particles smaller than 1 mm, 20 g of rock per culture, 25 °C, static exposure, and a 45-day incubation [101]. This design examined whether established cyanobacterial biomass could maintain growth and alter rocks in a nutrient-poor aqueous system. The comparison with abiotic treatments linked the elemental response to cyanobacterial presence, while the common cultivation conditions permitted strain and rock effects to be examined separately.

The cyanobacterial silicate studies indicate that mineral composition, solution chemistry, and duration can alter both release and secondary reactions. Bundeleva et al. [115] and Lamérand et al. [116] monitored silicate dissolution and mineral precipitation over time, while Demirel-Floyd et al. [117] obtained different biotic responses with Antarctic and Icelandic sediments under the same temperature. These contrasts indicate that cultivation conditions cannot be selected independently of the mineral material. A condition that favors detectable release from one silicate may produce limited dissolution or greater secondary precipitation with another.

Light regime and gas supply also influence the chemical environment. The rock-phosphate and basalt experiments used 16:8 h light–dark cycles [101,102,103], whereas continuous illumination was used for C. vulgaris cultivation with struvite [104]. Moed et al. [104] additionally compared air with 10% CO_2_. Light and CO_2_ determine photosynthetic carbon assimilation and can alter pH during cultivation. Time-resolved pH and dissolved inorganic carbon measurements may therefore help relate mineral responses to the illuminated and dark portions of the culture cycle.

The studies involving Fe phosphate and struvite indicate that the associated elements also need consideration. He et al. [111] observed Fe-associated flocculation during Fe-P release, while struvite supplied P, N, and Mg simultaneously [104,105]. BRP contains multiple mineral phases and elements, and release of Ca, Mg, Fe, Al, Si, or trace elements may affect growth, aggregation, EPS production, or nutrient acquisition. Analysis restricted to P and K may omit chemical changes that explain differences among cultures.

EPS production introduces an additional dependence on cultivation conditions. Babiak and Krzemińska [119] describe effects associated with nutrient limitation, light, salinity, temperature, and growth phase. Paper et al. [121] reported differences in EPS composition and cation adsorption between N-limited and N-sufficient cyanobacterial cultures. When EPS is evaluated during BRP cultivation, its concentration and composition may therefore be interpreted in relation to the nutritional and physiological state of the culture rather than only to mineral presence.

A two-stage cultivation arrangement may be compared with single-stage cultivation when separation between biomass production and mineral exposure is relevant. In the first arrangement, cells can be produced in a complete medium, washed, and transferred to a BRP-containing medium. This approach resembles the concentrated washed inoculum used in the basalt experiment [101]. A single-stage system allows biomass formation and mineral interaction to occur simultaneously, as in the phosphate and struvite experiments [102,103,104,105]. Comparison of the two arrangements could consider biomass, dissolved P and K, cellular nutrient content, EPS, pH, and residual-mineral changes.

Mineral loading also requires reporting in relation to composition and physical properties. The included studies expressed loading as total rock mass, mineral concentration, or supplied P concentration [101,102,103,104,105]. These denominators describe different aspects of exposure. For BRP, reporting mass per culture volume together with total P and K, particle-size distribution, specific surface area, glass content, and abundance of nutrient-bearing phases would allow release to be expressed per liquid volume, mineral mass, initial nutrient content, or exposed surface area. These values may be presented as complementary descriptors rather than converted into a single efficiency measure.

Fraction separation can further clarify the distribution of the mobilized elements. The liquid fraction describes P or K remaining in solution at the sampling time, biomass analysis describes cellular acquisition, EPS extraction may identify extracellularly retained elements, and residual-solid characterization can examine mineral alteration. An uninoculated BRP treatment describes release arising from mineral-medium reactions under the same light, temperature, and gas conditions. Mineral-free inoculated cultures characterize physiological responses without BRP, while soluble-P and soluble-K treatments indicate growth when nutrient acquisition does not depend on the mineral.

The mineral-culture studies included in Table 4 did not extend the same characterized material to soil and plant experiments. The agricultural literature describes soil and plant responses after application of microalgae or cyanobacteria, but these responses may involve nutrient addition through biomass, N fixation, EPS, phytohormones, or changes in soil microbial activity. For a BRP-phototroph treatment, comparisons among BRP alone, the microorganism alone, and their combined application would allow soil P and K fractions, plant nutrient acquisition, biomass, and yield to be related to the individual and combined components.

The available evidence identifies distinct but connected contributions. Cyanobacteria mobilized P from calcium-associated mineral sources [102,103], eukaryotic microalgae were able to influence P release from Fe-associated material in complementary experiments [111], and cyanobacteria increased multielement release from crystalline basalt [101]. Struvite studies showed that phototrophic growth can be maintained when a sparingly soluble mineral replaces conventional soluble nutrients [104,105]. These findings indicate that BRP experiments may examine P and K release concurrently while distinguishing changes in the liquid phase, cellular acquisition, EPS-associated elements, and residual-mineral composition. This experimental integration would allow the relevance of phototrophic mineral interactions to be evaluated without assuming that growth, dissolved nutrients, and mineral dissolution represent equivalent responses.

## 6. Mixed Phototrophic-Bacterial Systems: Evidence and Proposed Interactions in P and K Mobilization

Associations between photosynthetic microorganisms and bacteria have been examined as defined cocultures, pre-established biofilms, mixed communities, and separately cultivated organisms subsequently applied in combination. These experimental arrangements represent different degrees of contact and interaction between the microbial components. Defined cocultures have been used to examine metabolite exchange and responses occurring during shared cultivation [23,123,124], whereas biofilm and coinoculation studies have primarily addressed maintenance of nutrient-related activity, soil nutrient responses, plant nutrient acquisition, and crop performance [125,126,127,128,129,130,131,132]. A mixed photoautotrophic-heterotrophic community has also been evaluated in contact with a defined silicate mineral, providing mineralogical evidence of community-associated weathering, although Mg and Si rather than P or K were measured [133].

Table 5 organizes the studies according to microbial arrangement, evaluated system, experimental setting, measured response, and relationship with P or K mobilization. The distinction among coculture, pre-established biofilm, mixed community, and coinoculation indicates whether the microorganisms shared a cultivation system, were associated before soil application, or were produced separately before combined application. Mechanistic or mineralogical responses, soil or plant responses, and contextual evidence are also distinguished according to the experimental endpoint.

### 6.1. Evidence from Mineral, Soil, and Plant Systems

The soil and plant studies represented in Table 5 were concentrated on cyanobacterium-based biofilms and combined microbial inoculants. Biofilms containing *Anabaena torulosa* and diazotrophic or phosphate-solubilizing bacteria were evaluated in wheat and leguminous crops [125,126,127,130]. In wheat, nutrient-related activity remained detectable for up to 14 weeks after inoculation [125]. This result indicates maintenance of a functional response after application, although it does not determine whether every component of the biofilm remained viable or metabolically active throughout the evaluated period.

In leguminous crops, a biofilm containing *A. torulosa* and *Azotobacter chroococcum* was associated with increases of 80% in available N and 24% in available P relative to the control [126]. Associations of *A. torulosa* with *Rhizobium* spp. or *Mesorhizobium ciceri* were examined through available soil nutrients, nitrogenase and dehydrogenase activities, soil carbon-related variables, plant nutrient concentrations, biomass, and yield [127,130]. Considered as a group, these studies relate biofilm application to simultaneous biological, chemical, and plant responses. The occurrence of changes in several endpoints also indicates that available P represented one component of a broader response involving N fixation, microbial activity, root–microorganism interactions, and plant development.

Experiments in wheat and okra further showed that the magnitude of the response depended on microbial composition. Manjunath et al. [132] compared cyanobacteria applied with *Providencia* and *Alcaligenes* and reported differences among microbial combinations in plant growth, microbial activity, and soil nutrient availability. In okra, individual and combined cyanobacterial–eubacterial treatments were associated with different soil-nutrient, plant-nutrient, and yield responses [128]. Comparisons among individual and combined treatments are particularly informative in these experiments, as they indicate whether a response was retained, reduced, or increased after the organisms were applied together. The measured endpoints nevertheless integrate nutrient transformation, microbial activity, root development, and nutrient acquisition rather than a single P-mobilization process.

Rana et al. [129] extended this evidence to a two-year rice–wheat field sequence. Combined inoculation with *Anabaena oscillarioides* CR3, *Brevundimonas diminuta* PR7, and *Ochrobactrum anthropi* PR10 was associated with greater rice-grain N, P, and K concentrations and an approximately 21.2% increase in rice yield relative to the recommended-fertilizer treatment. The microbial combinations associated with the responses differed between rice and wheat [129], indicating that crop identity and cultivation phase influenced the agronomic expression of the inoculants. As mineral fertilizers were included, the nutrient concentrations measured in the grains represent acquisition from the combined fertilizer–soil–microorganism system. The study consequently contributes field evidence for nutrient acquisition and crop performance rather than direct mineral-release evidence.

The studies based on *A. torulosa* biofilms and other cyanobacterium-bacterium combinations show a recurring association between mixed inoculation, microbial activity, extractable soil nutrients, plant nutrient concentrations, and crop performance [125,126,127,128,129,130,132]. The responses were not uniform across microbial combinations, crops, or measured variables. This variation indicates that the presence of two functional groups alone does not define the agronomic response; compatibility between the organisms, crop-associated conditions, nutrient management, and duration of the experiment also contribute to the observed outcome.

Duan et al. [131] provide the most informative internal comparison concerning K-related responses. The authors separately cultivated the K-solubilizing bacterium *Paenibacillus sabinae* and the salt-tolerant cyanobacterium *Leptolyngbya* sp. RBD05 in brewery wastewater. After biomass recovery and washing, the organisms were applied to saline-alkali soil individually or in combination. The treatment represents the coinoculation of separately produced biomasses rather than the establishment of a consortium during a shared cultivation phase.

The combined treatment contained 50 mL of a *P. sabinae* suspension at 7 × 10^7^ CFU mL^−1^ and 50 mL of a *Leptolyngbya* sp. RBD05 suspension at 0.01 g dry matter mL^−1^ [131]. At 60 days, this treatment presented 169.24 mg available K kg^−1^ soil, corresponding to an increase of 26% relative to the control. The difference among treatments was no longer significant at 90 days, indicating that the treatment effect on the extractable soil K fraction depended on the sampling period.

Coinoculation was also associated with increases of 85% in wheat dry weight, 70% in root length, and 41% in the plant K:Na ratio relative to the control [131]. These responses connect the temporary change in soil-available K with plant growth and ionic balance under saline-alkali conditions. The plant response may have remained detectable after differences in the extractable K pool decreased, as K acquisition, exchange reactions, microbial immobilization, and changes in plant stress responses occurred throughout cultivation. Repeated measurements of soil K fractions and plant K accumulation would clarify the temporal relationship between the soil and plant responses.

The same experiment showed that the advantage of the combined treatment was nutrient-dependent. At 30 days, *P. sabinae* applied alone increased soil-available P by 71% relative to the control, whereas coinoculation produced a smaller response [131]. Differences among treatments decreased at subsequent evaluations. Thus, the phototrophic component coincided with a greater K-related response at 60 days but did not increase the early P response beyond that obtained with the bacterial treatment. The result indicates that a combined application can produce distinct interaction patterns for P and K within the same soil–plant experiment.

The comparison between individual and combined treatments in Duan et al. [131] represents evidence of a coinoculation effect, but not a formal demonstration of synergy. The experiment did not quantify K release from a characterized feldspar or BRP amendment, although the bacterial strain had been selected for a K-solubilization phenotype. Its principal contribution to BRP-oriented research is the demonstration that nutrient-specific and time-dependent responses can be examined by combining individual-organism controls, repeated soil measurements, and plant nutrient or stress indicators.

Lamérand et al. [133] examined a different portion of the interaction by exposing forsteritic olivine to a mixed photoautotrophic–heterotrophic community. The study measured Mg and Si release, surface alteration, etch-pit formation, and secondary Mg-bearing phases. Heterotrophic bacteria and their exometabolites were associated with changes in mineral dissolution, while phototrophic activity contributed to the chemical and biological conditions surrounding the particles [133]. Secondary-mineral formation also indicates that dissolved-element concentrations represented the balance between release from olivine and subsequent incorporation into newly formed phases.

Although olivine is not a relevant source of P or K, the experiment demonstrates how phototrophic activity, heterotrophic metabolism, surface alteration, dissolved elements, and secondary reactions can be evaluated within the same mineral-containing system. These analytical connections are applicable to studies with characterized BRP, in which measurements of dissolved P and K can be interpreted alongside microbial composition, residual-mineral phases, and particle-surface changes.

### 6.2. Metabolic Interactions and the Interpretation of Combined Responses

Defined cocultures provide evidence that interactions between photosynthetic microorganisms and bacteria can alter the growth and metabolism of both partners. Vitamin-B_12_-dependent microalgae may receive cobalamin from heterotrophic bacteria, while the bacterial partner uses photosynthetically derived organic carbon [23]. More recent defined systems have shown that the direction and magnitude of bacterial effects vary among phototrophic hosts. Deng et al. [134], using synthetic diatom–bacteria communities, found that individual bacterial strains could produce beneficial, neutral, or inhibitory effects depending on the microalgal species. This context dependence indicates that metabolic compatibility should be evaluated for each proposed combination rather than inferred from the functional description of the partners.

Metabolic exchange is not restricted to vitamins and bulk organic carbon. Chegukrishnamurthi et al. [135] compared axenic *Chlorella*, a bacterial mixture containing Rhizobium and Agrobacterium; direct coculture; and cultures connected only through the headspace. The volatile-organic-compound profiles differed among these arrangements, indicating that direct contact and diffusible metabolites can produce distinct interaction conditions. Astafyeva et al. [136] also reported changes in microalgal growth and transcriptional responses in a defined *Micrasterias radians-Dyadobacter* association. These studies do not concern mineral mobilization, but they demonstrate that microbial identity and the physical arrangement of the partners influence the responses observed during shared cultivation.

In a mineral-containing system, photosynthetically derived carbon may contribute to bacterial growth and extracellular metabolism, while bacterial respiration may alter inorganic-carbon availability to the phototrophic component [124,137]. The resulting relationship is expected to vary with carbon source, light regime, pH, aeration, nutrient availability, and relative abundance of the partners. Zhang et al. [138], for example, showed that inoculation ratio, light intensity, temperature, and pH affected the performance of a defined *Dunaliella salina*-*Halomonas mongoliensis* coculture. Although the evaluated response was phenol degradation rather than mineral dissolution, the experiment illustrates that an apparent coculture advantage can depend on the specific cultivation conditions used to establish and maintain the association.

EPS production and biofilm formation add a spatial component to these metabolic interactions. EPS can contribute to cell aggregation, surface attachment, and retention of cells or dissolved compounds within the biofilm environment [137]. Cheah and Chan [139] described polysaccharides, proteins, nucleic acids, and lipids as relevant components of microalgal biofilm matrices and emphasized that the measured composition depends on the extraction and analytical procedure. Experimental work with microalgal biofilms has also associated EPS coatings with increased initial attachment and cell retention on solid surfaces [140]. These observations concern artificial carrier surfaces rather than mineral particles, but they indicate measurable properties (attachment, bound EPS, biofilm coverage, and resistance to detachment) that can be examined during exposure to BRP.

For BRP-containing systems, biofilm formation could be investigated through microscopy, cell distribution on mineral particles, bound and soluble EPS fractions, and concentrations of metabolites near the mineral-solution interface. These measurements would determine whether the combined culture produces a distinct spatial organization compared with each component alone. Demonstration of attachment would not itself establish nutrient mobilization, but correspondence among biofilm development, mineral-surface changes, and P or K release would connect microbial organization with the mineral response.

The term synergy requires a comparison extending beyond the observation that a combined treatment exceeds either individual treatment. A formal evaluation should consider the expected additive response, equivalent total inoculum or biomass, comparable cultivation conditions, and the same analytical endpoint and sampling time. Under this definition, Duan et al. [131] identified a greater response to coinoculation for soil-available K at 60 days, but the study did not test whether this value exceeded an additive expectation. The smaller early-P response of the combined treatment relative to *P. sabinae* alone further indicates that the interaction was not consistently advantageous across nutrients. The terms “combined-treatment response” or “coinoculation effect” are therefore more appropriate for the available evidence.

**Figure 5:**
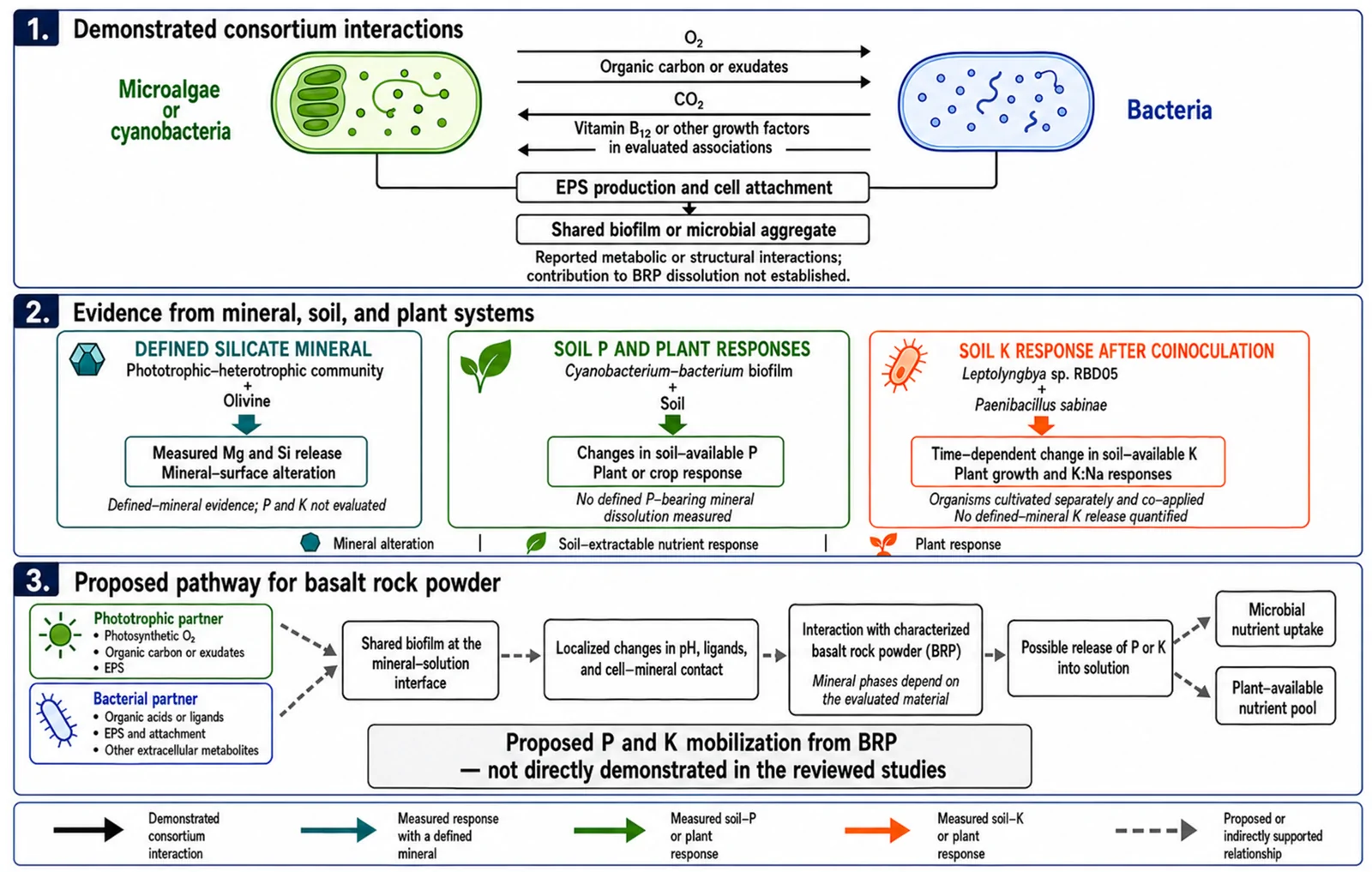
Experimental evidence and proposed interactions associated with phosphorus and potassium mobilization in microalgae-bacteria systems.

Figure 5 summarizes the experimentally observed interactions and the proposed relationships relevant to BRP. Demonstrated metabolic exchange, soil and plant responses, and mineral alteration should be represented separately from the pathways proposed for P and K release.

### 6.3. Environmental Conditions, Stability, and Assessment of Mixed Systems

The interpretation of mixed phototrophic-bacterial systems depends on how the microbial association is established. Defined coculture exposes both partners to the same light regime, pH, carbon sources, aeration, mineral loading, and incubation period. Separate cultivation followed by coinoculation allows each organism to be produced under conditions suited to its growth, but it does not determine whether the partners remain compatible or maintain their initial proportions after application. Biofilmed inoculants represent another arrangement in which spatial association is established before contact with soil [125,126,127,130]. These cultivation modes should be reported separately, as they describe different opportunities for metabolite exchange and population adjustment.

Initial inoculation ratio is particularly relevant when the phototrophic and bacterial partners present different growth rates. In the absence of repeated abundance measurements, the initial ratio does not describe consortium composition during later cultivation. Deng et al. [134] observed host-specific changes in synthetic diatom–bacteria communities, while Zhang et al. [138] identified inoculation ratio as one of the conditions affecting coculture performance. Measurements based on cell counts, chlorophyll, CFU, flow cytometry, or organism-specific molecular markers may indicate whether each component remains represented during contact with a mineral source.

Light and carbon availability also define the balance between phototrophic and heterotrophic metabolism. Light intensity and photoperiod affect photosynthetic growth and O_2_ production, whereas organic-carbon composition influences bacterial respiration and may permit mixotrophic growth of the phototrophic partner [141,142,143,144]. Medium pH affects carbon speciation, microbial physiology, and mineral reactions; Lacroux et al. [141], for example, associated the use of volatile fatty acids by microalgae with the fraction occurring in the undissociated form. Consequently, carbon assimilation and mineral dissolution should be measured as separate responses when organic acids or other carbon sources are included in mixed-system experiments.

The temporal behavior reported in soil experiments further indicates that microbial activity and nutrient responses may not remain synchronized. Nutrient-related activity of cyanobacterial biofilms was detectable for up to 14 weeks after wheat inoculation [125], but this measurement did not quantify the abundance of each microbial partner. In the experiment of Duan et al. [131], the coinoculation-associated K response was detected at 60 days but was not significant at 90 days. These results distinguish maintenance of biological activity from persistence of a difference in the extractable nutrient pool. Repeated measurements of microbial abundance, soil nutrient fractions, plant nutrient accumulation, and biomass can indicate whether changes in the consortium coincide with the beginning, maintenance, or decline of the nutrient response.

Evaluation of combined systems should also account for the possibility that one component benefits while the other is inhibited. Defined coculture experiments have reported beneficial, neutral, and inhibitory bacterial effects on different microalgal hosts [134]. Competition for P, K, trace elements, vitamins, or organic carbon may occur simultaneously with reciprocal metabolite exchange. Comparison of each monoculture with the combined culture can therefore include growth rates, viable biomass, nutrient incorporation into cells, extracellular metabolites, and mineral-associated responses. This design allows improved nutrient release to be distinguished from a change arising mainly from reduced cellular uptake or altered growth of one partner.

Community profiling and functional analyses may complement these measurements when consortium stability or attribution of microbial activity is an experimental objective. Amplicon sequencing can describe changes in community composition, whereas transcriptomic, proteomic, or metabolomic approaches may identify responses associated with nutrient transport, extracellular compounds, or mineral exposure [145,146,147]. These analyses are most informative when interpreted alongside dissolved P and K, microbial biomass, pH, metabolites, and mineral-surface characterization. Taxonomic or gene-expression data alone do not determine whether a measured nutrient originated from mineral dissolution.

An assessment of mixed systems containing BRP may therefore compare an abiotic mineral control, each microbial component separately, and the combined treatment under the same mineral loading and sampling schedule. Equal total inoculum across the individual and combined treatments would permit the interaction effect to be separated from an increase in the amount of applied biomass. Measurements of P and K in the liquid phase and microbial biomass, combined with pH, extracellular compounds, microbial abundance, residual-mineral phases, and surface alteration, would connect consortium composition with nutrient distribution and mineral response. Subsequent soil experiments could examine whether the responses observed during cultivation correspond to changes in soil nutrient fractions, plant acquisition, and biomass or yield.

The studies reviewed in this section indicate that mixed phototrophic–bacterial systems can maintain nutrient-related activity after soil application, alter extractable soil nutrient pools, and contribute to plant nutrient acquisition and crop responses [125,126,127,128,129,130,131,132]. Direct comparisons also show that combined application does not produce a uniform advantage across nutrients or sampling periods [131]. Mineral-weathering evidence obtained with olivine further shows that phototrophic and heterotrophic activities can occur within a shared mineral system [133]. These findings identify microbial arrangement, partner compatibility, cultivation conditions, temporal stability, and correspondence among mineral alteration, soil nutrient fractions, and plant acquisition as central variables for examining mixed systems containing BRP.

## 7. Formulation, Application, and Implementation of Microbial Bioinputs Associated with Mineral P and K Sources

### 7.1. Evidence Heterogeneity and Transfer to Field Application

The evidence summarized in Table 3, Table 4 and Table 5 was obtained using different microorganisms, mineral substrates, culture media, incubation periods, analytical methods, and experimental settings. The reported endpoints included solubilization indices, dissolved P or K concentrations, metabolite production, molecular responses, mineral-surface alterations, extractable soil nutrients, plant nutrient acquisition, biomass, and yield. These measurements represent different stages of the interactions among microorganisms, minerals, soil, and plants and cannot be combined into a common estimate of nutrient-mobilization efficiency. Quantitative results should therefore be interpreted within their respective mineral-microorganism systems rather than used to rank strains, microbial groups, or formulations.

Variation in the reported P and K values can arise from differences in the mineral substrate, cultivation system, sample processing, and calculation procedure. Mineralogical composition influences which P- or K-bearing phases are exposed to microbial interaction, while particle-size distribution and specific surface area affect the extent of mineral surface available for reaction [40,46,52,53]. Mineral concentration additionally defines the amount of solid material and the total quantity of P or K introduced into the experimental system. Culture-medium composition and carbon-source availability can affect microbial growth, medium acidification, and metabolite production, whereas pH, temperature, agitation, inoculum concentration, and incubation period influence microbial activity, gas transfer, cell-particle contact, and the duration of mineral exposure [12,13,14,15,16,17,71,75]. Sample-processing procedures introduce a further source of variation, since centrifugation speed, filtration pore size, digestion, and extraction conditions determine which dissolved, colloidal, particle-associated, or extractable fractions are included in the analytical measurement. Reported values may also be expressed as nutrient concentration in the liquid phase, proportion of the nutrient initially present, amount released per unit mass of mineral, solubilization index, soil-extractable nutrient, or nutrient incorporated into microbial or plant biomass. These expressions use different analytical fractions and denominators and should not be interpreted as equivalent measures of mobilization. Abiotic mineral controls, inoculated treatments without the mineral source, time-resolved measurements, and nutrient mass balances can help distinguish microbial-associated release from abiotic dissolution, cellular uptake, adsorption, precipitation, and analytical variation. Direct numerical comparisons are therefore appropriate only when mineral characteristics, mineral loading, cultivation conditions, sampling time, phase-separation procedure, analytical method, calculation basis, and experimental controls are sufficiently comparable.

Transfer from controlled cultivation to soil and field conditions introduces factors that are absent or restricted in culture-based assays. Soil buffering, nutrient sorption and precipitation, variable water and carbon availability, root activity, resident microbial communities, predation, and temporal changes in temperature and moisture may affect microbial establishment and nutrient availability [10,148,149,150]. O’Callaghan et al. [151] describe competition with resident microorganisms, limited establishment, environmental variability, and compatibility with agricultural practices as factors associated with inconsistent responses to soil microbial inoculants. P or K released during liquid cultivation may consequently be retained in soil phases, incorporated into microbial biomass, or absorbed by roots and may not remain in the soil-solution or extractable fractions measured after application.

Greenhouse and field responses may integrate mineral weathering, microbial persistence, fertilizer effects, root development, plant biostimulation, and interactions with the resident microbiota [13,69,73,74,125,126,127,128,129,131]. These responses provide evidence closer to agricultural application but do not necessarily identify the contribution of microbial-mineral mobilization. Experiments connecting inoculant persistence, mineral alteration, soil nutrient fractions, plant nutrient acquisition, and crop performance within the same system remain limited. Attribution of the nutrient source may require treatment structures that separate the microorganism, mineral amendment, and combined application, accompanied by mineralogical, chemical, or isotopic approaches when appropriate.

### 7.2. Product Composition, Formulation, and Storage Stability

Microbial bioinput development requires the intended product to be defined before formulation and stability studies are conducted. The active biological component may comprise viable vegetative cells, spores, photosynthetic cells, concentrated biomass, extracellular metabolites, or combinations of these components. According to Khan et al. [152], carrier selection, additives, microbial physiology, application method, and storage conditions are interconnected attributes of microbial bioformulation. Product characterization may therefore include microorganism identity, viable-cell or spore concentration, relative abundance of consortium members, metabolite or biomass markers, pH, moisture or water activity, carrier composition, and contaminant limits, as applicable. Díaz-Rodríguez et al. [153] also identify batch consistency and functional quality control as relevant stages in the transition from microbial cultivation to product development.

Products associated with BRP require additional specification of the mineral component. Mineralogical and chemical composition, particle-size distribution, mineral loading, moisture content, and the proportion of BRP incorporated into or applied with the microbial formulation may influence product homogeneity and the availability of P and K. A preparation containing cells cultivated with BRP but separated from the mineral before formulation differs from a product containing cells, extracellular metabolites, and residual-mineral particles. Cell-free culture fractions, dried microbial biomass, and microorganisms applied separately from BRP also represent distinct product compositions and should not be treated as interchangeable alternatives.

Liquid formulations can retain cells and extracellular compounds present in the culture medium and may require fewer processing stages than concentrated or dried products. Their composition may nevertheless change during storage through nutrient depletion, continued microbial metabolism, oxygen limitation, pH variation, sedimentation, metabolite transformation, and contamination [149,150]. Solid carriers and concentrated formulations can reduce storage volume and facilitate transport, although processing may expose cells to dehydration, thermal stress, osmotic changes, or contact with carrier components that affect survival [152].

Rock powder can be retained in the formulation when direct contact between microorganisms and mineral particles is intended. This configuration may preserve microbial aggregates or extracellular compounds associated with mineral surfaces and can allow microbe–mineral interactions initiated during cultivation to continue after application. Fine mineral particles may also promote sedimentation, formulation heterogeneity, equipment abrasion, nozzle obstruction, or changes in pH and ionic composition. These effects depend on mineral concentration, particle size, carrier characteristics, storage conditions, and the application system. Products in which cells are separated from BRP may be easier to homogenize and dose but do not preserve the same spatial association between cells, extracellular products, and mineral particles.

Shelf-life should be defined using predetermined acceptance criteria rather than storage duration alone. Stability studies may examine viable-cell or spore concentration, contamination, physicochemical properties, and retention of the intended functional response under specified combinations of time, temperature, light exposure, and packaging conditions. Fadiji et al. [154] identify inadequate formulation stability and loss of microbial functionality during storage as factors that can restrict product consistency. Viable-cell counts describe microbial survival but do not demonstrate that the stored microorganism retains its capacity to interact with the intended mineral source. Functional assessment after storage may include P or K release from a defined mineral, phosphatase activity, medium acidification, mineral attachment, or production of metabolites associated with the proposed product function.

Stability assessment of mixed microbial products requires additional consideration of consortium composition. Total viable counts may remain within an acceptance range even when individual populations decline at different rates. Differences in growth rate, oxygen demand, nutrient use, storage metabolism, and tolerance to dehydration may progressively alter the relative abundance of consortium members. Preservation of the initial microbial ratio and of the response associated with the combined culture should therefore be evaluated independently. Evidence that individual strains remain viable after storage does not demonstrate preservation of their interaction within the formulated consortium.

Bioencapsulation includes methods that differ in processing conditions, particle structure, encapsulation efficiency, and release behavior. Rojas-Sánchez et al. [155] describe ionic gelation and extrusion as approaches frequently used to produce hydrogel beads under comparatively mild conditions. Emulsification can produce smaller particles but introduces additional formulation and separation requirements. Spray drying enables rapid conversion into dry powders and may facilitate storage and distribution, although thermal and dehydration stresses can reduce cell recovery. Freeze drying is conducted at lower temperatures but generally requires longer processing times and greater energy input. Luft and Mazutti [156] indicate that the performance of these drying methods depends on microorganism physiology, process conditions, and the protective compounds included in the formulation.

Protective compounds may be incorporated into encapsulation or drying matrices to reduce cellular damage during processing and storage. Hu et al. [157] reported that encapsulation and osmotic protectants jointly affected microbial survival during storage, indicating that carrier performance cannot necessarily be interpreted independently of the protective additives. Evaluation of encapsulated products may therefore include viable-cell recovery after processing, encapsulation efficiency, particle size, moisture or water activity, mechanical stability, release kinetics, and retention of nutrient-mobilization activity. When BRP is incorporated into the carrier, mineral particles may modify bead structure, water transfer, density, settling behavior, and the release of cells or metabolites.

Experimental studies illustrate the dependence of encapsulation performance on the microorganism and carrier. Alginate formulations containing nanoclay or natural-char-derived particles preserved the viability and phosphate-solubilizing activity of *Pseudomonas putida* and *Pseudomonas kilonensis* for six months, although the results differed according to carrier and storage temperature [158]. Microbeads composed of rice bran, gum Arabic, and alginate presented an encapsulation efficiency of 94.11%, maintained bacterial survival for up to six months at 4 °C, and supported nutrient acquisition by wheat [159]. Isabel et al. [160] examined chitosan-encapsulated microbial biofertilizer and related storage stability to subsequent tomato responses. These results provide formulation-specific evidence and should not be generalized to other microorganisms, carrier compositions, storage temperatures, or application systems.

Assessment after storage should also consider microbial performance following exposure to the application environment. Graciano et al. [161] examined encapsulated Bacillus subtilis under salinity and temperature stresses, illustrating that survival during storage and persistence after soil application represent related but distinct stages of product evaluation. A formulation can retain viable cells during storage but provide limited release, establishment, or functional activity under soil conditions. Conversely, rapid carrier disintegration may support initial cell release without providing protection during subsequent environmental variation.

Formulation requirements may differ between heterotrophic bacteria and photosynthetic microorganisms. Microalgal and cyanobacterial products can contain living cultures, concentrated wet biomass, dried biomass, extracts, or cell-derived compounds, and these formats do not provide equivalent functions. Miranda et al. [162] identify preservation, standardization, and delivery as relevant considerations in the development of microalgal agricultural products. Drying can facilitate storage and transportation, but Hernández et al. [163] describe method-dependent changes in cell integrity and biochemical composition during microalgal processing. Products intended to deliver living photosynthetic cells require assessment of post-processing viability and recovery of photosynthetic activity. Products containing non-viable biomass or extracts should instead be characterized according to their composition and the biological response supporting the proposed function.

The formulation of viable microalgae-bacteria consortia presents additional constraints. Photosynthetic microorganisms require light during active growth, whereas prolonged dark storage may alter viability and metabolic activity. Bacterial respiration, nutrient consumption, and production of extracellular compounds can change pH, gas composition, and the physicochemical properties of the formulation. Encapsulation methods established for bacterial spores or vegetative bacterial cells cannot therefore be assumed to preserve photosynthetic microorganisms or mixed cultures. Shelf-life studies should reproduce the intended storage format and evaluate each microbial component separately together with the function attributed to the consortium.

### 7.3. Application Methods and Agronomic Evidence

Application methods should be selected in relation to formulation characteristics and the intended contact among the biological component, mineral source, soil, and plant root. Seed coating can position microorganisms near the emerging root but limits the amount of microbial biomass or mineral particles that can be delivered and can expose cells to desiccation or incompatible seed-treatment products. Soil drenching, furrow application, and fertigation can accommodate larger application volumes and position cells or metabolites in the rhizosphere. Granular or encapsulated formulations may protect microorganisms during storage and application, but their agronomic response depends on hydration, carrier disintegration, cell release, and movement through the soil [155,157,160].

Formulation-application compatibility should be evaluated before field use. Liquid products intended for spraying or fertigation require control of viscosity, sedimentation, aggregation, and particle size to reduce obstruction and uneven distribution. Products containing BRP may require agitation during application and equipment compatible with suspended mineral particles. Seed coatings require assessment of adhesion, drying, seed germination, and microbial survival after coating. Encapsulated products require specification of particle size and release behavior in relation to soil moisture. Reporting microbial dose, formulation mass or volume, BRP application rate, timing, frequency, and co-applied inputs would improve comparisons among experiments and support the interpretation of field responses [152,153].

Application rate cannot be represented by a universal concentration range. Microbial dose may be expressed as CFU per seed, CFU per gram or milliliter of product, biomass concentration, number of particles, or product volume per plant or cultivated area. These units describe different aspects of application and may not allow direct conversion when viability, carrier concentration, or release efficiency is not reported. BRP-associated products additionally require the microbial dose to be related to the mineral loading and field application rate. This information is necessary to determine whether the microorganism and mineral remain spatially associated after dilution and field distribution.

The agronomic studies summarized in Table 6 differ in experimental scale, formulation, application route, mineral source, crop, and measured response. The table distinguishes greenhouse, pot, controlled-cultivation, and field evidence and indicates whether the evaluated mineral was applied to the plant system or used only during microbial screening. This distinction limits the attribution of soil or plant responses to microbial release of P or K from a defined mineral source.

Bacterial inoculants evaluated in greenhouse and pot experiments were associated with changes in soil nutrient availability, plant nutrient acquisition, root development, biomass, or yield in tomato [12,66], wheat [73], tobacco [164], cotton [13], onion [71], maize [69], and apple [74]. Direct relevance to a BRP-based product depends on whether a defined mineral source was included in the plant experiment and whether the experimental design separated the effects of the microorganism, mineral, and combined treatment. Studies in which phosphate or K-bearing minerals were used only for preliminary screening demonstrate a microbial phenotype but do not provide direct evidence of nutrient release from that mineral under soil or plant conditions.

Microalgae-based treatments have also been associated with agronomic responses in rice, tomato, hawthorn, and strawberry [165,166,167,168]. In tomato cultivation, *Chlorella vulgaris* applied through foliar spraying or soil drenching was associated with changes in plant growth, fruit characteristics, and mineral composition, while combined application with cow manure produced the largest overall response among the evaluated treatments [166]. These effects cannot be attributed exclusively to mineral solubilization, since microalgal biomass and extracts may supply nutrients and biologically active compounds or influence root and rhizosphere processes [169,170,171]. Microalgal agronomic responses should therefore be interpreted according to product composition and application route rather than treated as direct evidence of microbial weathering.

Application route and experimental setting cannot be separated from product performance. Responses obtained after seed treatment, soil inoculation, foliar application, or application with a mineral amendment represent different exposure conditions. O’Callaghan et al. [151] indicate that microbial establishment, interactions with resident soil communities, environmental variation, and compatibility with agricultural practices contribute to variable field responses. Greenhouse and pot experiments can control soil, irrigation, temperature, and inoculation conditions but do not reproduce the temporal and spatial variability encountered in agricultural fields. Field validation should consequently include sites and seasons representative of the intended use and should document soil properties, background fertilization, climatic conditions, and agricultural management.

For BRP-associated products, field experiments should contain treatments that allow the effects of the biological and mineral components to be examined separately. Appropriate treatment structures may include an uninoculated control, BRP alone, the microbial formulation without BRP, and the combined formulation. A soluble-fertilizer treatment can provide an agronomic reference but does not replace the controls required to attribute responses to microbial-mineral interactions. Measurements of soil-available P and K, plant nutrient acquisition, biomass, and yield can then be combined with mineralogical or chemical analyses when identification of the nutrient source is required.

### 7.4. Regulatory, Commercial, and Technoeconomic Considerations

The legal classification of microbial products depends on their composition, proposed function, claims, and intended use. In Brazil, Normative Instruction No. 61/2020 establishes provisions applicable to organic fertilizers and biofertilizers, including specifications, guarantees, tolerances, registration, packaging, and labeling requirements [172]. Ordinance MAPA No. 52/2021 establishes technical requirements and authorized substances and practices for organic production systems [173]. Law No. 15,070/2024 subsequently established a national legal framework covering the production, registration, commercialization, use, research, packaging, labeling, transport, storage, inspection, and disposal of bioinputs [174].

Law No. 15.070/2024 assigns the federal agricultural-defense authority responsibility for defining classifications, specifications, minimum parameters, and complementary procedures. Detailed regulatory requirements should therefore be interpreted in conjunction with the implementing regulations and MAPA acts applicable when the product is developed or submitted for registration. The official regulatory sources should be reviewed again before manuscript resubmission, since the implementation of the legal framework remains subject to complementary provisions.

Products containing viable microorganisms, microbial metabolites, mineral particles, or combinations of these components may require different characterization and supporting evidence. Products intended to supply nutrients, alter nutrient availability, stimulate plant development, or act through viable microorganisms may not fall within the same technical category. Product development may consequently require microbial identification and traceability, compositional guarantees, contaminant control, stability assessment, functional testing, safety information, and labeling consistent with the demonstrated function [172,173,174,175]. Reis et al. [176] describe regulatory classification, quality assurance, biosafety documentation, and claim substantiation as factors influencing the progression of microbial inoculants from experimental preparation to commercial product.

Regulatory definitions and procedures also differ among jurisdictions. Santos et al. [177] describe the definitions, regulatory treatment, and commercialization prospects of biofertilizers in the United States, indicating that comparisons among markets require consideration of product composition, intended use, and claims. Such international evidence can illustrate common regulatory challenges but should not be used to infer the requirements applicable in Brazil. Products containing both viable microorganisms and BRP may require consideration of the identity and safety of the biological component together with mineral composition, contaminant concentrations, particle characteristics, and the agronomic function attributed to the combined formulation.

Commercial production involves stages that are not represented by small-volume cultivation experiments. These stages may include inoculum preparation, cultivation, harvesting or concentration, carrier incorporation, stabilization, packaging, storage, transport, and release testing. Díaz-Rodríguez et al. [153] associate scale-up with maintenance of microbial identity, viable concentration, functional activity, and batch consistency. Production specifications should define which variables are monitored during each stage and which acceptance criteria determine batch release. A product that meets a viable-cell specification but no longer presents the intended nutrient-mobilization response may not be functionally equivalent to the original experimental preparation.

Production requirements differ among bacterial spores, vegetative bacterial cells, and photosynthetic microorganisms. Bacterial inoculants can generally be produced in conventional fermentation systems, whereas microalgal cultivation may require illuminated reactors or open ponds, mixing, gas transfer, water management, harvesting, and biomass concentration. Harvesting, centrifugation, membrane separation, drying, extraction, and encapsulation may improve product concentration, handling, or storage but increase energy demand, equipment requirements, and processing costs [170,178,179,180]. Miranda et al. [162] indicate that production and formulation remain relevant limitations in the development of microalgae-based agricultural products.

Technoeconomic performance depends on the product configuration. Romero-García et al. [180] showed that cultivation, biomass concentration, hydrolysis, solid–liquid separation, and product recovery affected the simulated cost of a microalgal biofertilizer production process. Castro et al. [181] combined process scale-up, life-cycle assessment, and economic analysis for biofertilizer recovery from microalgal biomass. Although biomass-derived products differ from inoculants containing viable photosynthetic cells, these studies identify cultivation, downstream processing, energy demand, and equipment as relevant contributors to production feasibility. Results obtained for processed microalgal biomass should not be transferred directly to living microalgal or microalgae–bacteria formulations.

Incorporation of BRP introduces further production and logistical considerations. Mineral processing can include crushing, grinding, sieving, drying, transport, dust control, and homogenization with the biological formulation. Fine particle sizes may increase mineral surface area but can also increase grinding demand, dust formation, settling, and handling constraints. Regional availability of BRP may reduce raw-material transport and support the use of mining by-products, whereas long transport distances or additional micronization can offset part of this advantage [182]. The microbial and mineral production chains should therefore be assessed jointly rather than as independent components.

Commercial viability cannot be inferred from nutrient release under laboratory conditions alone. Production cost, shelf-life, storage temperature, packaging, distribution distance, application equipment, compatibility with existing agricultural operations, and application frequency contribute to the final cost of use. Fadiji et al. [154] associate limitations in formulation stability and field consistency with barriers to microbial-inoculant adoption. Reis et al. [176] further relate commercial development to production capacity, regulatory compliance, patent activity, and demonstration of agronomic value. Farmer adoption is likely to depend on ease of handling, compatibility with mineral-application practices, consistency of field responses, and evidence that the combined product provides an identifiable advantage relative to the separate application of the microorganism or BRP.

Economic assessment should include raw materials, inoculum preparation, cultivation, energy consumption, biomass recovery, formulation, packaging, quality control, storage, transport, field application, and disposal of residual materials. The magnitude and consistency of the agronomic response should be considered together with production cost. Use of low-cost nutrient streams, locally available carriers, concentrated wet biomass, or integrated systems involving wastewater treatment and nutrient recovery may reduce selected production costs [159,179,180]. These alternatives require evaluation of contamination risks, compositional variability, regulatory acceptance, and their effects on product stability.

Overall, implementation of BRP-associated microbial bioinputs requires the formulation to preserve the component responsible for the proposed function from production through field application. Co-formulation with mineral particles may support direct microbe–mineral contact but can introduce constraints related to homogeneity, storage, and application. Encapsulated, carrier-based, separated-cell, and cell-free products can offer different advantages in protection, handling, or standardization but represent functionally different configurations. Progress toward commercial application therefore depends on integrating product composition, functional shelf-life, application compatibility, regulatory classification, production scale, field validation, and technoeconomic assessment.

## 8. Conclusions and Future Perspectives

This review indicates that P and K release from rock powders depends on the mineral phases containing these nutrients and on particle properties, pH, solution composition, soil conditions, and experimental duration. Therefore, total elemental composition alone does not represent the amounts of P and K that may become available to plants.

The reviewed evidence is more developed for bacteria than for microalgae and cyanobacteria and is more extensive for P than for K. Culture-based nutrient-release and mechanistic evidence predominated, whereas soil and plant validation was less frequent and field evidence remained limited. Bacterial studies provide direct measurements of nutrient release from several mineral sources, whereas studies with photosynthetic microorganisms have examined fewer strains and substrates. Mixed phototrophic–bacterial systems have been associated with metabolic exchange, biofilm formation, soil nutrient responses, and plant growth. However, direct P or K release from BRP by a defined microalgae-bacteria or cyanobacteria-bacteria consortium has not been quantified.

The identified research gaps include comparisons of bacteria, photosynthetic microorganisms, and defined mixed cultures using the same characterized BRP and experimental conditions. Nutrient mass balances combined with mineralogical, surface, and molecular analyses could help distinguish mineral dissolution, microbial uptake, adsorption, and precipitation. Further evidence is also required to determine whether responses observed in culture are maintained across soils, crops, environmental conditions, and longer experimental periods.

Additional gaps concern the relationship between nutrient-mobilization activity, formulation stability, shelf-life, application strategy, and field performance. Regulatory requirements, biosafety, production costs, mineral processing, transport, and application costs also remain to be examined together with the magnitude and consistency of agronomic responses. Addressing these gaps could clarify whether BRP-based microbial products can progress from controlled experiments to stable and economically feasible agricultural inputs.

## Figures and Tables

**Table 3:** Experimental evidence of phosphorus and potassium mobilization from mineral sources by bacteria.

Microorganism	Element	Mineral Source and Category	Experimental Setting	Experimental Conditions	Reported Metabolite, Process, or Molecular Response	Quantitative Mobilization Result	Evidence Type	Reference
*Bacillus haynesii* ACP1	P	Phosphate rock (non-basalt P-bearing mineral)	Laboratory and bench-scale bioreactor	Optimized cultivation medium; 50 °C; 200 rpm; 24 h; subsequent cultivation in a 7 L bioreactor	Acid phosphatase; lactic, glutamic, and hydroxybenzoic acids	Not reported	Mechanistic response; culture-based assessment without quantitative nutrient-release measurement	[75]
*Bacillus velezensis*	P	Rock phosphate (non-basalt P-bearing mineral)	Laboratory followed by pot experiment	Culture-based rock-phosphate solubilization followed by plant evaluation	P-solubilization and plant-growth-promoting traits	Not reported	Culture-based solubilization phenotype and soil or plant response	[73]
*Bacillus* isolates	P	Tricalcium phosphate and Moroccan natural phosphate (non-basalt P-bearing minerals)	Laboratory followed by plant experiment	Twenty-four isolates screened using culture-based assays; selected strains evaluated with wheat	Organic acids, IAA, siderophores, and ACC-deaminase activity	Not reported	Culture-based solubilization phenotype, mechanistic response, and plant response	[70]
*Pseudomonas* sp. + *Serratia* sp.	P	Phosphate rock (non-basalt P-bearing mineral)	Laboratory followed by greenhouse pot experiment	SMRS1/MT11B culture system; 30 °C; 200 rpm; measurements up to 12 h	Gluconic, oxalic, citric, and malic acids	58.1 ± 0.28 mg P L^−1^ after 8 h; 88.1 ± 0.32 mg P L^−1^ after 12 h	Culture-based nutrient release, mechanistic response, and soil or plant response	[71]
*Serratia* sp. Bg22c and *Pseudomonas* sp. Bg32c	P and K	Tricalcium phosphate and feldspar (non-basalt P- and K-bearing minerals)	Laboratory followed by greenhouse experiment	NBRIP for P: 28 °C, 180 rpm, 48 h; Aleksandrov medium for K: 28 °C, 48 h	Gluconic acid for Bg22c; oxalic acid for Bg32c; IAA- and siderophore-related traits	P-solubilization indices: 3.83 and 2.85; K-solubilization indices: 2.50 and 4.70 for Bg22c and Bg32c, respectively; soluble P and K concentrations not reported	Culture-based solubilization phenotype, mechanistic response, and soil or plant response	[12]
*Paenibacillus sonchi* SBR5	P	Hydroxyapatite (non-basalt P-bearing mineral)	Laboratory	Pikovskaya medium; physiological and gene-expression measurements; metabolites assessed after approximately 20 h	Acetate and gluconate; expression of genes associated with P metabolism	Increase of approximately 1 mmol L^−1^ orthophosphate	Culture-based nutrient release and mechanistic or molecular response	[14]
*Bacillus aryabhattai* SK1-7	K	Potassium feldspar (non-basalt K-bearing mineral)	Laboratory followed by plant experiment	Feldspar concentration of 12 g L^−1^; 30 °C; 200 rpm; 7 days	Medium acidification, organic acids, extracellular compounds, and mineral-surface alteration	10.8 mg K L^−1^; reported solubilization proportion of 32.6%	Culture-based nutrient release, mechanistic or mineralogical response, and plant response	[16]
*Bacillus aryabhattai* SK1-7	K	Potassium feldspar (non-basalt K-bearing mineral)	Laboratory	Samples evaluated after 24, 96, and 168 h; genomic, transcriptomic, chemical, and mineral-surface analyses	Malic, formic, acetic, citric, and gluconic acids; polysaccharides; gene-expression responses	Not reported	Mechanistic, molecular, and mineralogical response without quantitative nutrient-release measurement	[15]
*Bacillus megaterium* Ag87 + *Lysinibacillus* sp. Ag94	P	FePO_4_·2H_2_O (non-basalt P-bearing mineral)	Laboratory, greenhouse, and field	NBRIP agar; 25 °C; 10 days; strains evaluated separately and in combination	IAA production and phosphate-solubilization phenotype	Not reported	Culture-based solubilization phenotype and soil or plant response	[69]
*Enterobacter* sp. LG7 (BIOTECH 10607)	P	Ca_3_(PO_4_)_2_ and AlPO_4_ (non-basalt P-bearing minerals)	Laboratory	NBRIP containing 5 g L^−1^ mineral substrate and 10 g L^−1^ glucose; 30 °C; 160 rpm; 7 days; inoculum of 10^7^ CFU mL^−1^	Gluconic acid	210.4 mg P L^−1^ from Ca_3_(PO_4_)_2_; 2.3 mg P L^−1^ from AlPO_4_ after 7 days	Culture-based nutrient release and mechanistic response	[72]
*Priestia megaterium* NK851	K	Potassium feldspar (non-basalt K-bearing mineral)	Laboratory	Liquid cultivation; 30 °C; 140 rpm; 5 days	Feldspar-binding proteins, including elongation factor Tu and enolase; mineral attachment; acidification was not identified as the sole explanation	157 mg K L^−1^ after 3 days; 222 mg K L^−1^ after 5 days	Culture-based nutrient release and mechanistic or molecular response	[17]
K-solubilizing rhizobacteria, including *Pseudomonas aeruginosa* A10	K	Insoluble K-bearing mineral used for screening (non-basalt mineral; composition not specified)	Laboratory followed by greenhouse experiment	Culture-based K-solubilization screening followed by greenhouse evaluation in saline-sodic soil	K-solubilization phenotype under moderately alkaline conditions; specific metabolites not reported	Not reported	Culture-based solubilization phenotype and soil or plant response	[13]

P, phosphorus; K, potassium; NBRIP, National Botanical Research Institute’s phosphate growth medium; IAA, indole-3-acetic acid; ACC, 1-aminocyclopropane-1-carboxylate; CFU, colony-forming units. “Not reported” indicates that the corresponding information was not presented in the article as a directly extractable value or experimental description. Culture-based nutrient release indicates measurement of soluble P or K during cultivation with a mineral source. A culture-based solubilization phenotype indicates halo formation or a solubilization index without a corresponding soluble-nutrient concentration. Mechanistic or mineralogical responses include metabolites, molecular responses, microbial attachment, and mineral-surface alterations. Soil or plant responses include changes in soil nutrient availability, plant nutrient acquisition, growth, biomass, or yield. Evidence categories are not mutually exclusive and do not constitute a hierarchy of methodological quality. Concentrations, percentage release, and solubilization indices represent different analytical endpoints and are not directly comparable.

**Table 4:** Experimental evidence of phosphorus and potassium release or utilization by microalgae and cyanobacteria cultivated with mineral sources.

Organism	Element	Mineral Source and Category	Experimental Setting	Culture Medium	Experimental Conditions	Quantitative Mobilization Result	Evidence Type	Interpretation and Limitation	Reference
*Westiellopsis prolifica*	P	Mussorie rock phosphate and TCP (non-basalt P-bearing minerals)	Laboratory	N-free BG-11; K_2_HPO_4_ replaced by the mineral source at 10, 20, or 30 mg P L^−1^; KCl supplied to maintain K	28 ± 2 °C; 16:8 h light/dark; 2500–3000 lux; 2% P-starved inoculum; 35 days	At 30 mg P L^−1^, available P on day 7 reached 0.32 µg mL^−1^ with rock phosphate and 0.38 µg mL^−1^ with TCP	Culture-based nutrient release	Available P was measured from two insoluble substrates under the same culture conditions; the result represents culture-based release rather than soil or plant evidence.	[102]
*Anabaena variabilis*	P	Mussorie rock phosphate and TCP (non-basalt P-bearing minerals)	Laboratory	N-free BG-11; K_2_HPO_4_ replaced by the mineral source at 10, 20, or 30 mg P L^−1^; KCl supplied to maintain K	28 ± 2 °C; 16:8 h light/dark; 2500–3000 lux; 2% P-starved inoculum; 35 days	At 30 mg P L^−1^, available P on day 7 reached 0.35 µg mL^−1^ with rock phosphate and 0.37 µg mL^−1^ with TCP	Culture-based nutrient release	Available P was measured from two insoluble substrates under the same culture conditions; the result represents culture-based release rather than soil or plant evidence.	[102]
*Anabaena variabilis*, *Nostoc muscorum*, *Tolypothrix tenuis*, *Aulosira fertilissima*, and *Westiellopsis prolifica*	P	Mussorie rock phosphate and TCP (non-basalt P-bearing minerals)	Laboratory	N-free BG-11; soluble phosphate replaced by mineral P at 10, 20, or 30 mg P L^−1^	Cultures evaluated for growth, N fixation, and available P during incubation	*A. variabilis* presented an available-P value of 0.224 µg mL^−1^ with TCP supplied at 20 mg P L^−1^	Culture-based nutrient release and physiological response	Growth, N fixation, and P release were evaluated as distinct endpoints. Responses varied with strain, mineral source, substrate concentration, and incubation period.	[103]
*Anabaena cylindrica* PCC 6309	K	Crystalline basalt and rhyolite (basalt-related and non-basalt silicate rocks)	Laboratory	Sterile double-distilled water containing 10 mM NaNO_2_ and 20 g mineral material; no additional nutrients or buffer	Rock particles < 1 mm; 25 °C; 16:8 h light/dark; 3000 lux; static culture; approximately 10^9^ cells mL^−1^; 45 days	Dissolved K after 45 days: 135.32 µmol L^−1^ from basalt and 16.94 µmol L^−1^ from rhyolite	Culture-based nutrient release from basalt and rhyolite	Dissolved K was measured directly in cultures containing crystalline rocks; the conditions do not represent soil or field exposure.	[101]
*Phormidium* sp. OU_10	K	Crystalline basalt and rhyolite (basalt-related and non-basalt silicate rocks)	Laboratory	Same mineral-based medium described above	Same conditions described above	Dissolved K after 45 days: 56.02 µmol L^−1^ from basalt and 23.15 µmol L^−1^ from rhyolite	Culture-based nutrient release from basalt and rhyolite	The dissolved-K response differed between the two rock substrates under the evaluated culture conditions.	[101]
*Leptolyngbya* sp. OU_13	K	Crystalline basalt and rhyolite (basalt-related and non-basalt silicate rocks)	Laboratory	Same mineral-based medium described above	Same conditions described above	Dissolved K after 45 days: 65.89 µmol L^−1^ from basalt and 28.53 µmol L^−1^ from rhyolite	Culture-based nutrient release from basalt and rhyolite	The dissolved-K response differed between the two rock substrates under the evaluated culture conditions.	[101]
*Synechococcus elongatus* PCC 6301	K	Crystalline basalt and rhyolite (basalt-related and non-basalt silicate rocks)	Laboratory	Same mineral-based medium described above	Same conditions described above	Dissolved K after 45 days: 43.26 µmol L^−1^ from basalt and 16.22 µmol L^−1^ from rhyolite	Culture-based nutrient release from basalt and rhyolite	The study provided culture-based K-release evidence for a unicellular cyanobacterium; soil and plant responses were not evaluated.	[101]
*Chroococcidiopsis* sp. 029	K	Crystalline basalt and rhyolite (basalt-related and non-basalt silicate rocks)	Laboratory	Same mineral-based medium described above	Same conditions described above	Dissolved K after 45 days: 45.61 µmol L^−1^ from basalt and 11.09 µmol L^−1^ from rhyolite	Culture-based nutrient release from basalt and rhyolite	The dissolved-K response varied between the cyanobacterial strain and rock substrates under the evaluated conditions.	[101]
*Gloeocapsa* sp. OU_20	K	Crystalline basalt and rhyolite (basalt-related and non-basalt silicate rocks)	Laboratory	Same mineral-based medium described above	Same conditions described above	Dissolved K after 45 days: 41.70 µmol L^−1^ from basalt; growth was not detected with rhyolite	Culture-based nutrient release from basalt	K release was measured for basalt, while the absence of growth with rhyolite prevented direct comparison between the two rocks.	[101]
*Chlorella vulgaris*	P	Synthetic struvite (non-basalt P-bearing mineral)	Laboratory	Modified BBM in which struvite replaced soluble P, N, and Mg salts; K salts and NaCl were supplemented	Struvite at 721, 1442, or 2163 mg L^−1^; continuous illumination at 25 µmol photons m^−2^ s^−1^; gas flow of 100 mL min^−1^ with air or 10% CO_2_; mainly 7 days	Direct soluble-P release was not reported	Contextual or indirect evidence based on nutrient utilization	Growth and lipid production indicated nutrient supply from struvite, but the experiment did not isolate an organism-specific dissolution rate.	[104]
*Chlorella vulgaris* 211-11b	P	Recovered struvite (non-basalt P-bearing mineral)	Laboratory	Adapted BBM; struvite supplied the total P requirement and part of the N requirement	1 L Erlenmeyer flasks; 300 rpm; initial OD of 0.1; triplicate cultures; daily pH adjustment; up to 20 days	Organism-specific P-release value was not reported	Contextual or indirect evidence based on nutrient utilization	Growth on struvite was evaluated separately from constant-pH physicochemical dissolution tests; the dissolution rate cannot be assigned directly to the microorganism.	[105]
*Limnospira* sp. PCC 8005	P	Recovered struvite (non-basalt P-bearing mineral)	Laboratory	Adapted Zarrouk medium; struvite supplied the total P requirement and part of the N requirement	1 L flasks; 300 rpm; initial OD of 0.1; pH 9; triplicate cultures; up to 14 days	Organism-specific P-release value was not reported	Contextual or indirect evidence based on nutrient utilization	Growth on struvite was demonstrated, but the dissolution rate was determined in separate physicochemical experiments.	[105]

P, phosphorus; K, potassium; TCP, tricalcium phosphate; BBM, Bold’s Basal Medium; OD, optical density. “Not reported” indicates that the corresponding variable was not presented as an organism-specific, directly extractable value. Culture-based nutrient release indicates measurement of dissolved or available P or K during cultivation with a mineral source. Contextual or indirect evidence based on nutrient utilization indicates microbial growth or biomass production in the presence of a mineral without an organism-specific nutrient-release measurement. Categories are not mutually exclusive and do not represent a formal hierarchy of methodological quality. Dissolved nutrient concentrations, growth responses, phosphatase activity, and physicochemical dissolution rates represent different experimental endpoints and are not directly comparable. Cyanobacteria are photosynthetic prokaryotes and are presented separately from eukaryotic microalgae.

**Table 5:** Evidence related to phosphorus and potassium mobilization by mixed phototrophic-bacterial systems.

Phototrophic Component or Community	Bacterial Component	Microbial Arrangement	Mineral Source or Evaluated System	Experimental Setting	Evaluated Response	Reported Result	Type of Evidence	Interpretation and Limitation	Reference
Photoautotrophic microbial community	Heterotrophic bacterial community	Mixed microbial community	Forsteritic olivine (defined non-basalt mineral; P and K were not evaluated)	Laboratory	Mg and Si release, mineral-surface alteration, and secondary-mineral formation	Heterotrophic bacteria and their exometabolites were associated with changes in Mg and Si release and with etch-pit formation on olivine.	Mechanistic or mineralogical response in a defined mineral system	The study connects microbial-community activity with dissolved-element concentrations and mineral-surface alteration in a defined silicate system. Its relevance to P and K mobilization is mechanistic and indirect, as the evaluated mineral did not contain these elements as target nutrients.	[133]
*Anabaena torulosa*	*Azotobacter chroococcum*, *Mesorhizobium ciceri*, *Serratia marcescens*, or *Pseudomonas striata*	Pre-established cyanobacterium–bacterium biofilms	Biofilmed inoculants applied to wheat soil without a defined mineral source	Controlled soil and plant experiment	N-fixation potential and maintenance of nutrient-related activity	N-fixation potential increased by up to 50%, and nutrient-related activity was detected for up to 14 weeks.	Contextual or indirect soil and plant evidence	Nutrient-related activity remained detectable after inoculation, indicating persistence of a functional response under the evaluated soil conditions. The abundance and viability of each microbial component and the release of P or K from a defined mineral were not quantified.	[125]
*Anabaena torulosa*	*Azotobacter chroococcum*	Pre-established cyanobacterium–bacterium biofilm	Biofilmed inoculant applied to leguminous crops without a defined mineral source	Soil and plant experiment	Soil-available N and P	Available N and P increased by 80% and 24%, respectively, relative to the control.	Soil and plant response	The treatment was associated with changes in extractable soil N and P, indicating a nutrient-related response after biofilm application. The experiment did not distinguish mineral dissolution from organic-matter transformations, microbial turnover, or root-associated processes.	[126]
*Anabaena torulosa*	*Rhizobium* spp.	Pre-established cyanobacterium–bacterium biofilm	Biofilmed inoculants applied to chickpea, pea, and lentil without a defined mineral source	Soil and plant experiment	Available P, soil carbon, polysaccharides, dehydrogenase and nitrogenase activities, plant nutrient concentrations, and crop responses	Changes in available P and microbial, enzymatic, soil, and plant variables were reported.	Soil and plant response	The study connects biofilm inoculation with soil P availability, microbial activity, and plant responses across leguminous crops. The P response represented one component of a broader biological and agronomic response, and its mineral source was not identified.	[130]
*Anabaena torulosa*	*Mesorhizobium ciceri*	Pre-established cyanobacterium–bacterium biofilm	Biofilmed inoculant applied in chickpea cultivation without a defined mineral source	Soil and plant experiment	Available soil P and N, N_2_ fixation, dehydrogenase activity, plant growth, nutrient acquisition, and yield	Changes in soil nutrient availability, microbial activity, chickpea growth, nutrient acquisition, and yield were reported.	Soil and plant response	The experiment was associated with the biofilmed treatment with soil nutrient, microbial, and agronomic responses in chickpea. These concurrent responses indicate a broader inoculant effect and do not assign the change in available P to dissolution of a specific mineral phase.	[127]
*Anabaena oscillarioides* WRC3 and *Anabaena torulosa* WRC4	*Providencia* sp. WRB4 and *Alcaligenes* sp. WRB10	Individual and combined inoculation	Wheat cultivated in unsterilized soil receiving the recommended mineral-fertilizer dose; no defined rock-derived mineral source	Pot experiment	Plant growth, crop biomass, fluorescein-diacetate hydrolysis, and soil dehydrogenase activity	*Providencia* sp. WRB4 applied alone presented the largest plant-growth responses, followed by *A. oscillarioides* WRC3. Responses to combined inoculation varied among microbial combinations, and treatments containing cyanobacteria were associated with greater soil microbial activity.	Contextual or indirect evidence	The study indicates that microbial identity and combination influenced plant and soil responses. The larger response obtained with an individual bacterial treatment also shows that combined inoculation did not consistently exceed the corresponding individual treatments. P or K release from a mineral source was not evaluated.	[132]
*Anabaena* spp. and *Calothrix* sp.	*Azotobacter* sp. and *Providencia* spp.	Individual inoculation, defined consortia, and a pre-established biofilm	Okra cultivated in sterilized silt-loam soil receiving NPK fertilizer; no defined rock-derived mineral source	Pot experiment under net-house conditions	Available soil nutrients, microbial biomass carbon, enzyme activities, root growth, crop biomass, yield, and soil bacterial-community profiles	The *Anabaena* sp.-*Providencia* sp. CR1 + PR3 treatment presented the highest microbial biomass carbon and available P among the evaluated treatments. *Azotobacter* sp. applied alone presented the highest root weight and yield.	Soil and plant response	The study associates specific microbial treatments with soil-P, microbial, root, and yield responses. The different treatment rankings across endpoints indicate that the treatment associated with greater available P was not the same treatment associated with the largest yield response. As NPK fertilizer was supplied, the origin of the available P was not isolated.	[128]
*Anabaena oscillarioides* CR3	*Brevundimonas diminuta* PR7 and *Ochrobactrum anthropi* PR10	Combined field inoculation	Rice–wheat cropping sequence receiving mineral fertilizer without a defined rock-derived mineral source	Field	Soil microbial activity, grain N, P, and K concentrations, and crop yield	The combined treatment was associated with greater rice-grain N, P, and K concentrations and an approximately 21.2% increase in rice yield relative to the recommended-fertilizer treatment. Responses differed between rice and wheat and among microbial combinations.	Field soil and plant response	The study connects combined inoculation with nutrient acquisition and crop performance under field conditions. The measured responses represent the fertilizer–soil–microorganism-plant system and do not identify mineral-derived P or K release.	[129]
*Lobomonas rostrata*	*Mesorhizobium loti*	Defined coculture	Semicontinuous coculture without vitamin B_12_, an added organic-carbon source, or a mineral substrate	Laboratory	Vitamin B_12_ supply, photosynthetically fixed carbon, growth, and population equilibrium	The bacterium supplied vitamin B_12_ to the microalga, while photosynthetically fixed carbon was transferred to the bacterial partner. A stable population relationship was maintained over multiple generations and was altered by vitamin B_12_ or organic-carbon supplementation.	Contextual or indirect evidence	The experiment demonstrates reciprocal metabolite exchange and population adjustment in a defined coculture. It informs the evaluation of metabolic compatibility and stability, although no P- or K-bearing mineral was included.	[23]
*Leptolyngbya* sp. RBD05	*Paenibacillus sabinae*	Separate cultivation followed by coinoculation	Saline-alkali soil cultivated with wheat without the addition of a defined mineral source	Pot experiment	Soil-available K and P, plant K:Na ratio, dry weight, root length, and stress-related responses	At 60 days, the combined treatment presented 169.24 mg available K kg^−1^ soil, 26% above the control. Wheat dry weight, root length, and K:Na ratio increased by 85%, 70%, and 41%, respectively. At 30 days, the highest available-P response was obtained with *P. sabinae* alone. Differences in available K were not significant at 90 days.	Comparative soil and plant response following coinoculation	Coinoculation was associated with greater soil-available K at 60 days and with wheat growth and K:Na responses under saline-alkali conditions. The absence of a significant difference in available K at 90 days indicates that the treatment comparison depended on the sampling period. The larger early-P response obtained with the bacterial treatment alone further indicates that the coinoculation effect differed between nutrients. The organisms were cultivated separately, and a defined K-bearing mineral was not added to the soil.	[131]

P, phosphorus; K, potassium; N, nitrogen; Mg, magnesium; Si, silicon. A defined mineral system indicates that a characterized mineral substrate was included and that dissolved elements or mineral-surface responses were measured. Soil and plant responses include changes in extractable nutrients, nutrient acquisition, biomass, growth, or yield. Contextual or indirect evidence includes metabolic exchange, maintenance of nutrient-related activity, or other microbial interactions evaluated without a defined P- or K-bearing mineral. Coculture refers to organisms maintained within the same cultivation system, whereas coinoculation refers to separately produced organisms subsequently applied in combination. A pre-established biofilm represents an association formed before its application to soil. Evidence categories describe different experimental endpoints and do not constitute a hierarchy of methodological quality. The quantitative results represent study-specific conditions and should not be interpreted as a common measure of mixed-system efficiency.

**Table 6:** Agronomic performance of phosphorus- and potassium-solubilizing bacteria and microalgae-based bioinputs in greenhouse, pot, controlled cultivation, and field experiments.

Experimental Condition	Microorganism or Product	Mineral or Nutrient Source	Crop	Formulation and Application	Main Agronomic Response	Reference
Greenhouse experiment under axenic soil conditions	*Pseudomonas* spp. and *Bacillus* sp.	Natural RP; tricalcium phosphate used for microbial screening	Tomato (*Solanum lycopersicum*)	Seeds coated with selected isolates at (10^6) CFU g^−1^; inoculated plants subsequently cultivated with natural RP	Improved seed germination and root colonization; increased shoot and root length and biomass; higher plant P, Ca, protein, sugar, and chlorophyll contents	[66]
Pot experiment	*Bacillus velezensis*	RP alone or combined with 50, 75, or 100% of the recommended SSP and urea rates	Wheat (*Triticum aestivum*)	Bacterial bioinoculation combined with the respective fertilizer treatments; application volume NR	Increased soil and leaf P and nitrate concentrations; treatments combining the bacterium with mineral fertilizers improved plant growth and yield-related variables	[73]
Greenhouse experiment	Phosphate- and K-solubilizing bacterial isolates, including *Pseudomonas* and *Bacillus* spp.	RP and insoluble K-bearing sources used for microbial screening	Tomato (*Solanum lycopersicum*)	Bacterial inoculation of seedlings; application rate NR	Increased plant growth and nutrient acquisition; selected isolates also produced IAA and showed antagonistic activity against the tomato bacterial canker agent	[12]
Greenhouse pot experiment	*Paenibacillus* sp. SKL51, *Klebsiella oxytoca* SKT41, and *Enterobacter hormaechei* PTG11	Native and poorly available soil K; insoluble K-bearing sources used for microbial screening	Tobacco (*Nicotiana tabacum*)	Bacterial suspensions applied to the root zone; application rate NR	Increased soil nutrient availability, plant nutrient acquisition, seedling height, and biomass; PTG11 produced the strongest overall growth-promoting response	[164]
Greenhouse pot experiment	*Pseudomonas aeruginosa* A10 and other K-solubilizing rhizobacteria	Insoluble K-bearing source used for microbial screening; saline–sodic soil used for plant cultivation	Cotton (*Gossypium* sp.)	Bacterial inoculation applied to the root zone; application rate NR	Increased plant height, root growth, and shoot and root dry biomass under saline–sodic conditions; strain A10 retained K-solubilizing activity under moderately alkaline conditions	[13]
Greenhouse pot experiment	Coculture of *Pseudomonas* sp. A18 and *Serratia* sp. C7	RP included in the bacterial cultivation medium	Onion (*Allium cepa*)	Coculture at approximately (10^8) CFU mL^−1^; 10 mL per experimental unit applied twice weekly, totaling 160 mL over two months	Seed germination exceeded 90%; total dry weight increased from (38 \pm 5) mg in the water control to (69 \pm 13) mg following coculture application	[71]
Greenhouse pot experiment	*Bacillus megaterium* Ag87 and *Lysinibacillus* sp. Ag94, applied individually or in combination	Soil P and phosphate fertilizer treatments	Maize (*Zea mays*)	Seed or soil inoculation with individual strains or their combination; application rate NR	Both strains exhibited phosphate-solubilizing and plant growth-promoting capacity; the effect of combined inoculation varied with experimental conditions and P availability	[69]
Pot experiment	*Paenibacillus mucilaginosus*	Poorly available soil K and K-bearing mineral fractions	Apple seedlings (*Malus domestica*)	Bacterial inoculation applied to the root zone; application rate NR	Increased seedling growth, plant K uptake, and soil K availability; changes in rhizosphere conditions were associated with enhanced K mobilization	[74]
Pot experiment	Encapsulated *Myroides gitamensis* BSH-3	Tricalcium phosphate incorporated into the microbeads; nutrients naturally present in the cultivation soil	Wheat (*Triticum aestivum*)	Air-dried microbeads composed of rice bran, gum Arabic, alginate, and bacterial cells applied to the soil	Increased germination, shoot and root length, fresh and dry biomass, and plant P and K uptake compared with the corresponding controls	[159]
Pot experiment	Microalgal biofertilizer containing *Chlorella* and/or cyanobacterial biomass, according to the treatment	Microalgal biomass used as a complementary nutrient source	Rice (*Oryza sativa*)	Microalgal biomass applied as a biofertilizer; application rate NR	Increased plant growth, yield-related variables, and seed productivity compared with the untreated control	[165]
Controlled cultivation experiment	Living *Chlorella vulgaris* biomass	Microalgal biomass applied alone or combined with cow manure	Tomato (*Solanum lycopersicum*)	Foliar spraying, soil drenching, or *C. vulgaris* combined with cow manure; different microalgal concentrations evaluated	Improved plant growth, fruit size, mineral composition, soluble solids, sugars, protein, vitamin C, and postharvest shelf-life; the combination with cow manure produced the strongest overall response	[166]
Field experiment	Live microalgae-based biofertilizer	Live microalgal culture combined with conventional fertilization	Hawthorn (*Crataegus* spp.)	Three doses applied through root application or foliar spraying	Increased fruit yield by 15.7–29.6%; the greatest increase occurred with the intermediate dose applied to the roots; fruit quality improved without increasing GHG emission intensity	[167]
Field experiment	Microalgae-based biostimulant formulations	Microalgal biomass or extracts used in combination with the crop fertilization program	Strawberry (*Fragaria × ananassa*)	Soil and/or foliar application according to the experimental treatment; application rate NR	Improved vegetative development, yield, fruit characteristics, and biochemical quality parameters; responses varied with formulation and application strategy	[168]

Greenhouse experiment: plants cultivated under greenhouse conditions without a clear indication that containers were used; greenhouse pot experiment: plants grown in containers under greenhouse conditions; pot experiment: plants grown in containers without a clearly specified greenhouse environment; controlled cultivation experiment: plants cultivated under controlled or semi-controlled conditions without a clear description of pot or greenhouse use; field experiment: plants cultivated directly under field or commercial production conditions. CFU, colony-forming units; GHG, greenhouse gas; IAA, indole-3-acetic acid; K, potassium; NR, not reported; P, phosphorus; RP, rock phosphate; SSP, single superphosphate. “Not reported” indicates that the corresponding information was not provided in the article or could not be extracted directly from its experimental description. Responses refer to effects reported relative to the corresponding uninoculated, untreated, or conventionally fertilized control in each study.

## Data Availability

No new data were created or analyzed in this study. Data sharing is not applicable to this article.

## References

[1] Calicioglu O., Flammini A., Bracco S., Bellù L., Sims R. (2019). The Future Challenges of Food and Agriculture: An Integrated Analysis of Trends and Solutions. Sustainability.

[2] Hina N.S. (2024). Global meta-analysis of nitrate leaching vulnerability in synthetic and organic fertilizers over the past four decades. Water.

[3] Ramos C.G., Querol X., Oliveira M.L.S., Pires K., Kautzmann R.M., Oliveira L.F.S. (2015). A Preliminary Evaluation of Volcanic Rock Powder for Application in Agriculture as Soil a Remineralizer. Sci. Total Environ..

[4] Swoboda P., Doering T.F., Hamer M. (2022). Remineralizing Soils? The Agricultural Usage of Silicate Rock Powders: A review. Sci. Total Environ..

[5] Ramos C.G., Hower J.C., Blanco E., Oliveira M.L.S., Theodoro S.H. (2022). Possibilities of Using Silicate Rock Powder: An Overview. Geosci. Front..

[6] Vienne A., Poblador S., Portillo-Estrada M., Hartmann J., Ijiehon S., Wade P., Vicca S. (2022). Enhanced Weathering Using Basalt Rock Powder: Carbon Sequestration, Co-Benefits and Risks in a Mesocosm Study with Solanum Tuberosum. Front. Clim..

[7] Kelland M.E., Wade P.W., Lewis A.L., Taylor L.L., Sarkar B., Andrews M.G., Lomas M.R., Cotton T.E.A., Kemp S.J., James R.H. (2020). Increased Yield and CO_2_ Sequestration Potential with the C4 Cereal Sorghum Bicolor Cultivated in Basaltic Rock-Amended Agricultural Soil. Glob. Chang. Biol..

[8] Luchese A.V., de Castro Leite I.J.G., da Silva Giaretta A.P., Alves M.L., Pivetta L.A., Missio R.F. (2023). Use of Quarry Waste Basalt Rock Powder as a Soil Remineralizer to Grow soybean and Maize. Heliyon.

[9] Luchese A.V., de Castro Leite I.J.G., Alves M.L., dos Santos Vieceli J.P., Pivetta L.A., Missio R.F. (2023). Can Basalt Rock Powder Be Used as an Alternative Nutrient Source for Soybeans and Corn?. J. Soil Sci. Plant Nutr..

[10] Uroz S., Picard L., Turpault M.-P. (2022). Recent Progress in Understanding the Ecology and Molecular Genetics of Soil Mineral Weathering Bacteria. Trends Microbiol..

[11] Wild B., Gerrits R., Bonneville S. (2022). The Contribution of Living Organisms to Rock Weathering in the Critical Zone. Npj Mater. Degrad..

[12] Bouizgarne B., Bakki M., Boutasknit A., Banane B., El Ouarrat H., Ait El Maalem S., Amenzou A., Ghousmi A., Meddich A. (2023). Phosphate and Potash Solubilizing Bacteria from Moroccan Phosphate Mine Showing Antagonism to Bacterial Canker Agent and Inducing Effective Tomato Growth Promotion. Front. Plant Sci..

[13] Zhao Y., Liang H., Zhang J., Chen Y., Dhital Y.P., Zhao T., Wang Z. (2024). Isolation and Characterization of Potassium-Solubilizing Rhizobacteria (KSR) Promoting Cotton Growth in Saline-Sodic Regions. Microorganisms.

[14] Brito L.F., López M.G., Straube L., Passaglia L.M.P., Wendisch V.F. (2020). Inorganic Phosphate Solubilization by Rhizosphere Bacterium *Paenibacillus sonchi*: Gene Expression and Physiological Functions. Front. Microbiol..

[15] Chen Y., Yang H., Shen Z., Ye J. (2022). Whole-Genome Sequencing and Potassium-Solubilizing Mechanism of Bacillus aryabhattai SK1-7. Front. Microbiol..

[16] Chen Y., Ye J., Kong Q. (2020). Potassium-Solubilizing Activity of *Bacillus aryabhattai* SK1-7 and Its Growth-Promoting Effect on *Populus alba* L.. Forests.

[17] Wu X., Zhao Z., Zhao Z., Zhang Y., Li M., Yu Q. (2023). Analysis of the Potassium-Solubilizing Priestia Megaterium Strain NK851 and Its Potassium Feldspar-Binding Proteins. Int. J. Mol. Sci..

[18] Bossa R., Di Colandrea M., Salbitani G., Carfagna S. (2024). Phosphorous utilization in microalgae: Physiological aspects and applied implications. Plants.

[19] Nawaz T., Saud S., Gu L., Khan I., Fahad S., Zhou R. (2024). Cyanobacteria: Harnessing the Power of Microorganisms for Plant Growth Promotion, Stress Alleviation, and Phytoremediation in the Era of Sustainable Agriculture. Plant Stress.

[20] Nahas E. (1996). Factors Determining Rock Phosphate Solubilization by Microorganisms Isolated from Soil. World J. Microbiol. Biotechnol..

[21] Rodríguez H., Fraga R. (1999). Phosphate Solubilizing Bacteria and Their Role in Plant Growth Promotion. Biotechnol. Adv..

[22] García-Berumen J.A., Flores de la Torre J.A., de los Santos-Villalobos S., Espinoza-Canales A., Echavarría-Cháirez F.G., Gutiérrez-Bañuelos H. (2025). Phosphorus Dynamics and Sustainable Agriculture: The Role of Microbial Solubilization and Innovations in Nutrient Management. Curr. Res. Microb. Sci..

[23] Kazamia E., Czesnick H., Van Nguyen T.T., Croft M.T., Sherwood E., Sasso S., Hodson S.J., Warren M.J., Smith A.G. (2012). Mutualistic Interactions between Vitamin B_12_-dependent Algae and Heterotrophic Bacteria Exhibit Regulation. Environ. Microbiol..

[24] Amin S.A., Parker M.S., Armbrust E.V. (2012). Interactions between Diatoms and Bacteria. Microbiol. Mol. Biol. Rev..

[25] Cerozi B.d.S., Fitzsimmons K. (2016). Use of *Bacillus* Spp. to Enhance Phosphorus Availability and Serve as a Plant Growth Promoter in Aquaponics Systems. Sci. Hortic..

[26] Dyhrman S.T. (2016). Nutrients and Their Acquisition: Phosphorus Physiology in Microalgae. The Physiology of Microalgae.

[27] Guo D.-J., Yang G.-R., Singh P., Wang J.-J., Lan X.-M., Singh R.K., Guo J., Dong Y.-D., Li D.-P., Yang B. (2025). Comprehensive Analysis of the Physiological and Molecular Responses of Phosphate-Solubilizing Bacterium *Burkholderia gladioli* DJB4-8 in Promoting Maize Growth. Front. Plant Sci..

[28] Guo J., Zhang Y., Dai Q., Luo X., Wang W., Xie Y., Chen Z., Peng W. (2025). Cultivating Microalgal Biofilms on Three Soils under Exogenous Phosphorus and Iron. Bioresour. Technol..

[29] Vassilev N., Vassileva M., Nikolaeva I. (2006). Simultaneous P-Solubilizing and Biocontrol Activity of Microorganisms: Potentials and Future Trends. Appl. Microbiol. Biotechnol..

[30] Mongeon P., Paul-Hus A. (2016). The Journal Coverage of Web of Science and Scopus: A Comparative Analysis. Scientometrics.

[31] Martín-Martín A., Orduna-Malea E., Thelwall M., López-Cózar E.D. (2018). Google Scholar, Web of Science, and Scopus: A Systematic Comparison of Citations in 252 Subject Categories. J. Informetr..

[32] Falagas M.E., Pitsouni E.I., Malietzis G.A., Pappas G. (2008). Comparison of PubMed, Scopus, Web of Science, and Google Scholar: Strengths and Weaknesses. FASEB J..

[33] Basak B.B., Sarkar B., Naidu R. (2021). Environmentally Safe Release of Plant Available Potassium and Micronutrients from Organically Amended Rock Mineral Powder. Environ. Geochem. Health.

[34] Khan F., Siddique A.B., Shabala S., Zhou M., Zhao C. (2023). Phosphorus Plays Key Roles in Regulating Plants’ Physiological Responses to Abiotic Stresses. Plants.

[35] Wang Y., Wu W.-H. (2013). Potassium Transport and Signaling in Higher Plants. Annu. Rev. Plant Biol..

[36] Anda M., Shamshuddin J., Fauziah C.I. (2015). Improving Chemical Properties of a Highly Weathered Soil Using Finely Ground Basalt Rocks. Catena.

[37] Filippelli G.M. (2008). The Global Phosphorus Cycle: Past, Present, and Future. Elements.

[38] Dorozhkin S.V. (2010). Calcium Orthophosphates as Bioceramics: State of the Art. J. Funct. Biomater..

[39] Sparks D.L., Stewart B.A. (1987). Potassium Dynamics in Soils. Advances in Soil Science.

[40] Manning D.A.C. (2010). Mineral Sources of Potassium for Plant Nutrition. A Review. Agron. Sustain. Dev..

[41] Volf M.R., Benites V.M., Azevedo A.C., Moraes M.F., Tiritan C.S., Rosolem C.A. (2023). Soil mineralogy and K reserves in soils from the Araguaia River valley, Brazil. Geoderma Reg..

[42] Bhat M.A., Tomar D., Grewal K.S., Sahoo J., Dar E.A. (2023). Non-exchangeable potassium release and supplying power of soils of Haryana, Northwest India. J. Plant Nutr..

[43] Das D., Sahoo J., Raza M.B., Barman M., Das R. (2022). Ongoing soil potassium depletion under intensive cropping in India and probable mitigation strategies. A review. Agron. Sustain. Dev..

[44] Guidry M.W., Mackenzie F.T. (2003). Experimental Study of Igneous and Sedimentary Apatite Dissolution: Control of pH, Distance from Equilibrium, and Temperature on Dissolution Rates. Geochim. Cosmochim. Acta.

[45] Oelkers E.H. (2001). General Kinetic Description of Multioxide Silicate Mineral and Glass Dissolution. Geochim. Cosmochim. Acta.

[46] White A.F., Brantley S.L. (2003). The Effect of Time on the Weathering of Silicate Minerals: Why Do Weathering Rates Differ in the Laboratory and Field?. Chem. Geol..

[47] Wilson M.J. (2004). Weathering of the Primary Rock-Forming Minerals: Processes, Products and Rates. Clay Miner..

[48] Hinsinger P., Bell M.J., Kovar J.L., White P.J. (2021). Rhizosphere Processes and Root Traits Determining the Acquisition of Soil Potassium. Improving Potassium Recommendations for Agricultural Crops.

[49] Gislason S.R., Oelkers E.H. (2003). Mechanism, Rates, and Consequences of Basaltic Glass Dissolution: II. An Experimental Study of the Dissolution Rates of Basaltic Glass as a Function of PH and Temperature. Geochim. Cosmochim. Acta.

[50] Gudbrandsson S., Wolff-Boenisch D., Gislason S.R., Oelkers E.H. (2011). An Experimental Study of Crystalline Basalt Dissolution from 2 ≤ PH ≤ 11 and Temperatures from 5 to 75 °C. Geochim. Cosmochim. Acta.

[51] Blum J.D., Erel Y. (1995). A Silicate Weathering Mechanism Linking Increases in Marine 87Sr/ 86Sr with Global Glaciation. Nature.

[52] Palandri J.L., Kharaka Y.K. (2004). A Compilation of Rate Parameters of Water-Mineral Interaction Kinetics for Application to Geochemical Modeling.

[53] Mesfin K.G., Wolff-Boenisch D., Gislason S.R., Oelkers E.H. (2023). Effect of Cation Chloride Concentration on the Dissolution Rates of Basaltic Glass and Labradorite: Application to Subsurface Carbon Storage. Minerals.

[54] Manning D.A.C. (2018). Innovation in Resourcing Geological Materials as Crop Nutrients. Nat. Resour. Res..

[55] Moore D.M., Reynolds R.C. (1989). X-Ray Diffraction and the Identification and Analysis of Clay Minerals.

[56] Eberl D.D. (2003). User Guide to RockJock—A Program for Determining Quantitative Mineralogy from X-Ray Diffraction Data.

[57] U.S. EPA (2014). Method 6020B Inductively Coupled Plasma-Mass Spectrometry.

[58] Vanderkloot E., Ryan P. (2023). Quantifying the effect of grain size on weathering of basaltic powders: Implications for negative emission technologies via soil carbon sequestration. Appl. Geochem..

[59] Korchagin J., Escosteguy P.A.V., Bortoluzzi E.C. (2022). Nutrient Transfer in Rangelands under Rock Powder Amendment. J. Plant Nutr. Soil Sci..

[60] Beerling D.J., Epihov D.Z., Kantola I.B., Masters M.D., Reershemius T., Planavsky N.J., Reinhard C.T., Jordan J.S., Thorne S.J., Weber J. (2024). Enhanced weathering in the US Corn Belt delivers carbon removal with agronomic benefits. Proc. Natl. Acad. Sci. USA.

[61] Skov K., Wardman J., Healey M., McBride A., Bierowiec T., Cooper J., Edeh I., George D., Kelland M.E., Mann J. (2024). Initial Agronomic Benefits of Enhanced Weathering Using Basalt: A Study of Spring Oat in a Temperate Climate. PLoS ONE.

[62] Holden F.J., Davies K., Bird M.I., Hume R., Green H., Beerling D.J., Nelson P.N. (2024). In-field carbon dioxide removal via weathering of crushed basalt applied to acidic tropical agricultural soil. Sci. Total Environ..

[63] Somavilla A., Caner L., Bortoluzzi E.C., Santanna M.A., Rheinheimer dos Santos D. (2021). P-Legacy Effect of Soluble Fertilizer Added with Limestone and Phosphate Rock on Grassland Soil in Subtropical Climate Region. Soil Tillage Res..

[64] Tonello M.S., Korchagin J., Bortoluzzi E.C. (2021). Environmental Agate Mining Impacts and Potential Use of Agate Residue in Rangeland. J. Clean. Prod..

[65] Oliveira C.d.F., Silva G.N., Marcante N.C., Vasconcelos V., Mandro M.A.E., Santos E.F. (2025). Assessing the Agronomic Efficiency of Rock Dust as a Nutrient Source in Agriculture. Rev. Ciênc. Agronômica.

[66] Bakki M., Banane B., Marhane O., Esmaeel Q., Hatimi A., Barka E.A., Azim K., Bouizgarne B. (2024). Phosphate Solubilizing *Pseudomonas* and *Bacillus* Combined with Rock Phosphates Promoting Tomato Growth and Reducing Bacterial Canker Disease. Front. Microbiol..

[67] Rfaki A., Zennouhi O., Nassiri L., Ibijbijen J. (2018). Soil Properties Related to the Occurrence of Rock Phosphate-Solubilizing Bacteria in the Rhizosphere Soil of Faba Bean (*Vicia faba* L.) in Morocco. Soil Syst..

[68] Rfaki A., Zennouhi O., Aliyat F.Z., Nassiri L., Ibijbijen J. (2020). Isolation, Selection and Characterization of Root-Associated Rock Phosphate Solubilizing Bacteria in Moroccan Wheat (*Triticum aestivum* L.). Geomicrobiol. J..

[69] Massucato L.R., Almeida S.R.d.A., Silva M.B., Mosela M., Zeffa D.M., Nogueira A.F., de Lima Filho R.B., Mian S., Higashi A.Y., Teixeira G.M. (2022). Efficiency of Combining Strains Ag87 (*Bacillus megaterium*) and Ag94 (*Lysinibacillus* sp.) as Phosphate Solubilizers and Growth Promoters in Maize. Microorganisms.

[70] Azaroual S.E., Hazzoumi Z., El Mernissi N., Aasfar A., Meftah Kadmiri I., Bouizgarne B. (2020). Role of Inorganic Phosphate Solubilizing Bacilli Isolated from Moroccan Phosphate Rock Mine and Rhizosphere Soils in Wheat (*Triticum aestivum* L.) Phosphorus Uptake. Curr. Microbiol..

[71] Blanco-Vargas A., Rodríguez-Gacha L.M., Sánchez-Castro N., Garzón-Jaramillo R., Pedroza-Camacho L.D., Poutou-Piñales R.A., Rivera-Hoyos C.M., Díaz-Ariza L.A., Pedroza-Rodríguez A.M. (2020). Phosphate-Solubilizing *Pseudomonas* sp., and *Serratia* sp., Co-Culture for *Allium cepa* L. Growth Promotion. Heliyon.

[72] Damo J.L.C., Pedro M., Sison M.L. (2024). Phosphate solubilization and plant growth promotion by *Enterobacter* sp. isolate. Appl. Microbiol..

[73] Afzal A., Bahader S., Ul Hassan T., Naz I., Din A. (2023). Rock Phosphate Solubilization by Plant Growth-Promoting *Bacillus velezensis* and Its Impact on Wheat Growth and Yield. Geomicrobiol. J..

[74] Chen Y., Yang X., Li Z., An X., Ma R., Li Y., Cheng C. (2020). Efficiency of Potassium-Solubilizing Paenibacillus Mucilaginosus for the Growth of Apple Seedling. J. Integr. Agric..

[75] Abdelgalil S.A., Kaddah M.M.Y., Duab M.E.A., Abo-Zaid G.A. (2022). A Sustainable and Effective Bioprocessing Approach for Improvement of Acid Phosphatase Production and Rock Phosphate Solubilization by Bacillus Haynesii Strain ACP1. Sci. Rep..

[76] Sheng X.F., He L.Y. (2006). Solubilization of Potassium-Bearing Minerals by a Wild-Type Strain of *Bacillus edaphicus* and Its Mutants and Increased Potassium Uptake by Wheat. Can. J. Microbiol..

[77] Guo S., Feng B., Xiao C., Wang Q., Zhou Y., Chi R. (2021). Effective Solubilization of Rock Phosphate by a Phosphate-Tolerant Bacterium *Serratia* sp.. Geomicrobiol. J..

[78] Shen Y.-Q., Bonnot F., Imsand E.M., RoseFigura J.M., Sjölander K., Klinman J.P. (2012). Distribution and Properties of the Genes Encoding the Biosynthesis of the Bacterial Cofactor, Pyrroloquinoline Quinone. Biochemistry.

[79] Wang L., Wang J., Yuan J., Tang Z., Wang J., Zhang Y. (2023). Long-Term Organic Fertilization Strengthens the Soil Phosphorus Cycle and Phosphorus Availability by Regulating the pqqC- and phoD-Harboring Bacterial Communities. Microb. Ecol..

[80] Han M., Zhu X., Ruan C., Wu H., Chen G., Zhu K., Liu Y., Wang G. (2024). Micro-Biophysical Interactions at Bacterium-Mineral Interfaces Determine Potassium Dissolution. Environ. Technol. Innov..

[81] Flemming H.-C., Wingender J. (2010). The Biofilm Matrix. Nat. Rev. Microbiol..

[82] Zhou Y., Zhang T., Jin S., Chen S., Zhang Y. (2021). Effects of *Escherichia coli* Alkaline Phosphatase PhoA on the Mineralization of Dissolved Organic Phosphorus. Water.

[83] Van den Berghe M., Merino N., Nealson K.H., West A.J. (2021). Silicate Minerals as a Direct Source of Limiting Nutrients: Siderophore Synthesis and Uptake Promote Ferric Iron Bioavailability from Olivine and Microbial Growth. Geobiology.

[84] Nath D., Maurya B.R., Meena V.S. (2017). Documentation of Five Potassium- and Phosphorus-Solubilizing Bacteria for Their K and P-Solubilization Ability from Various Minerals. Biocatal. Agric. Biotechnol..

[85] Liu Y.-Q., Wang Y.-H., Kong W.-L., Liu W.-H., Xie X.-L., Wu X.-Q. (2020). Identification, Cloning and Expression Patterns of the Genes Related to Phosphate Solubilization in *Burkholderia multivorans* WS-FJ9 under Different Soluble Phosphate Levels. AMB Express.

[86] Byloos B., Maan H., Van Houdt R., Boon N., Leys N. (2018). The Ability of Basalt to Leach Nutrients and Support Growth of *Cupriavidus metallidurans* CH34 Depends on Basalt Composition and Element Release. Geomicrobiol. J..

[87] Zhang S., Ying G., Liu T., Yang J., Zhu E., Sun X., Gu J.-D., Yan L. (2025). Basalt Rock Weathering by *Peribacillus simplex* from Wudalianchi Volcanos in NE China and Implications for Fe and Si Biogeochemical Cycling. Int. Biodeterior. Biodegrad..

[88] Nautiyal C.S. (1999). An Efficient Microbiological Growth Medium for Screening Phosphate Solubilizing Microorganisms. FEMS Microbiol. Lett..

[89] Ateş Ö. (2023). Phosphate Solubilizing Bacteria Isolation Medium: Rock Phosphate or Tricalcium Phosphate?. Geomicrobiol. J..

[90] Xue Y., Xiao C., Zhang Y., Chi R. (2021). Screening of High-Efficiency Potassium-Dissolving Strains and Optimization of the Potassium-Dissolving Process. Physicochem. Probl. Miner. Process..

[91] Sánchez-González M.E., Mora-Herrera M.E., Wong-Villarreal A., De La Portilla-López N., Sánchez-Paz L., Lugo J., Vaca-Paulín R., Del Aguila P., Yañez-Ocampo G. (2023). Effect of pH and Carbon Source on Phosphate Solubilization by Bacterial Strains in Pikovskaya Medium. Microorganisms.

[92] Brucker E., Kernchen S., Spohn M. (2020). Release of Phosphorus and Silicon from Minerals by Soil Microorganisms Depends on the Availability of Organic Carbon. Soil Biol. Biochem..

[93] Picard L., Blanco Nouche C., Cochet C., Turpault M.-P., Uroz S. (2023). Mineral Weathering by *Collimonas pratensis* PMB3(1) as a Function of Mineral Properties, Solution Chemistry and Carbon Substrate. Npj Mater. Degrad..

[94] Blanco Nouche C., Cochet C., Turpault M.-P., Uroz S. (2024). Feedback Effect of the Size of Mineral Particles on the Molecular Mechanisms Employed by *Caballeronia mineralivorans* PML1(12) to Weather Minerals. Npj Mater. Degrad..

[95] Barin M., Asadzadeh F., Hosseini M., Hammer E.C., Vetukuri R.R., Vahedi R. (2022). Optimization of Biofertilizer Formulation for Phosphorus Solubilizing by *Pseudomonas fluorescens* Ur21 via Response Surface Methodology. Processes.

[96] Ahmad A., Yasmin S., Yahya M., Akhtar K., Khaliq S., Akhtar N. (2024). Bio-Solubilization of Rock Phosphate Using Acidophilic Bacteria and Effect of Bio-Organic-Phosphate Composite on the Growth of Wheat. Geomicrobiol. J..

[97] Pang F., Li Q., Solanki M.K., Wang Z., Xing Y.-X., Dong D.-F. (2024). Soil Phosphorus Transformation and Plant Uptake Driven by Phosphate-Solubilizing Microorganisms. Front. Microbiol..

[98] Wang Y., Zhang W., Müller T., Lakshmanan P., Liu Y., Liang T., Wang L., Yang H., Chen X. (2023). Soil Phosphorus Availability and Fractionation in Response to Different Phosphorus Sources in Alkaline and Acid Soils: A Short-Term Incubation Study. Sci. Rep..

[99] Torres-Cuesta D., Mora-Motta D., Chavarro-Bermeo J.P., Olaya-Montes A., Vargas-Garcia C., Bonilla R., Estrada-Bonilla G. (2023). Phosphate-Solubilizing Bacteria with Low-Solubility Fertilizer Improve Soil P Availability and Yield of Kikuyu Grass. Microorganisms.

[100] Biswas S.S., Biswas D.R., Ghosh A., Sarkar A., Das A., Roy T. (2022). Phosphate-Solubilizing Bacteria Inoculated Low-Grade Rock Phosphate Can Supplement P Fertilizer to Grow Wheat in Sub-Tropical Inceptisol. Rhizosphere.

[101] Olsson-Francis K., Simpson A.E., Wolff-Boenisch D., Cockell C.S. (2012). The Effect of Rock Composition on Cyanobacterial Weathering of Crystalline Basalt and Rhyolite. Geobiology.

[102] Yandigeri M.S., Yadav A.K., Srinivasan R., Kashyap S., Pabbi S. (2011). Studies on mineral phosphate solubilization by cyanobacteria Westiellopsis and Anabaena. Microbiology.

[103] Jaiswal A., Mishra R., Koli D.K., Sharma V.K., Pabbi S. (2019). Evaluation of growth, nitrogen fixation and P-solubilizing ability of diazotrophic cyanobacteria under mineral phosphorus sources. Indian J. Agric. Sci..

[104] Moed N.M., Lee D.-J., Chang J.-S. (2015). Struvite as alternative nutrient source for cultivation of microalgae Chlorella vulgaris. J. Taiwan Inst. Chem. Eng..

[105] Muys M., González Cámara S.J., Derese S., Spiller M., Verliefde A., Vlaeminck S.E. (2023). Dissolution rate and growth performance reveal struvite as a sustainable nutrient source to produce a diverse set of microbial protein. Sci. Total Environ..

[106] Evens T.J., Koenig R. (2002). Phosphatase Expression by *Chlorella vulgaris* (Chlorophyceae) Is Mediated by Internal Phosphorus Levels and External PH. J. Phycol..

[107] Tazisong I.A., Senwo Z.N., He Z. (2015). Phosphatase Hydrolysis of Organic Phosphorus Compounds. Adv. Enzym. Res..

[108] Yandigeri M.S., Yadav A.K., Meena K.K., Pabbi S. (2010). Effect of mineral phosphates on growth and nitrogen fixation of diazotrophic cyanobacteria *Anabaena variabilis* and *Westiellopsis prolifica*. Antonie Van Leeuwenhoek.

[109] Yandigeri M.S., Meena K.K., Srinivasan R., Pabbi S. (2011). Effect of mineral phosphate solubilization on biological nitrogen fixation by diazotrophic cyanobacteria. Indian J. Microbiol..

[110] Cameron H.J., Julian G.R. (1988). Utilization of hydroxyapatite by cyanobacteria as their sole source of phosphate and calcium. Plant Soil.

[111] He L., Chen Y., Chen S., Wu X., Liu J. (2022). Effects of *Chlorella vulgaris* on phosphorus release from ferric phosphate sediment by consecutive cultivations. R. Soc. Open Sci..

[112] Afkairin A., Ippolito J.A., Stromberger M., Davis J.G. (2021). Solubilization of organic phosphorus sources by cyanobacteria and a commercially available bacterial consortium. Appl. Soil Ecol..

[113] Caille C., Duhamel S., Latifi A., Rabouille S. (2024). Adaptive Responses of Cyanobacteria to Phosphate Limitation: A Focus on Marine Diazotrophs. Environ. Microbiol..

[114] Solovchenko A., Plouviez M., Khozin-Goldberg I. (2024). Getting grip on phosphorus: Potential of microalgae as a vehicle for sustainable usage of this macronutrient. Plants.

[115] Bundeleva I.A., Ménez B., Augé T., Bodénan F., Recham N., Guyot F. (2014). Effect of Cyanobacteria *Synechococcus* PCC 7942 on Carbonation Kinetics of Olivine at 20 °C. Miner. Eng..

[116] Lamérand C., Shirokova L.S., Petit M., Bénézeth P., Rols J.L., Pokrovsky O.S. (2022). Kinetics and mechanisms of cyanobacterially induced precipitation of magnesium silicate. Geobiology.

[117] Demirel-Floyd C., Soreghan G.S., Elwood Madden M.E. (2022). Cyanobacterial weathering in warming periglacial sediments: Implications for nutrient cycling and potential biosignatures. Permafr. Periglac. Process..

[118] Mustoe G.E. (2018). Biogenic weathering: Solubilization of iron from minerals by epilithic freshwater algae and cyanobacteria. Microorganisms.

[119] Babiak W., Krzemińska I. (2021). Extracellular polymeric substances (EPS) as microalgal bioproducts: A review of factors affecting EPS synthesis and application in flocculation processes. Energies.

[120] Ciempiel W., Czemierska M., Szymańska-Chargot M., Zdunek A., Wiącek D., Jarosz-Wilkołazka A., Krzemińska I. (2022). Soluble extracellular polymeric substances produced by *Parachlorella kessleri* and *Chlorella vulgaris*: Biochemical characterization and assessment of their cadmium and lead sorption abilities. Molecules.

[121] Paper M., Jung P., Koch M., Lakatos M., Nilges T., Brück T.B. (2023). Stripped: Contribution of cyanobacterial extracellular polymeric substances to the adsorption of rare earth elements from aqueous solutions. Front. Bioeng. Biotechnol..

[122] Stigliano L., Wild B., Benzerara K., Ackerer P., Travert C., Knauss K.G., Daval D. (2025). In-Situ Laboratory Monitoring of Cyanobacterial Influence on Calcite Dissolution. Npj Mater. Degrad..

[123] Ramanan R., Kang Z., Kim B.-H., Cho D.-H., Jin L., Oh H.-M., Kim H.-S. (2015). Phycosphere Bacterial Diversity in Green Algae Reveals an Apparent Similarity across Habitats. Algal Res..

[124] Fuentes J., Garbayo I., Cuaresma M., Montero Z., González-del-Valle M., Vílchez C. (2016). Impact of Microalgae-Bacteria Interactions on the Production of Algal Biomass and Associated Compounds. Mar. Drugs.

[125] Swarnalakshmi K., Prasanna R., Kumar A., Pattnaik S., Chakravarty K., Shivay Y.S., Singh R., Saxena A.K. (2013). Evaluating the influence of novel cyanobacterial biofilmed biofertilizers on soil fertility and plant nutrition in wheat. Eur. J. Soil Biol..

[126] Prasanna R., Triveni S., Bidyarani N., Babu S., Yadav K., Adak A., Khetarpal S., Pal M., Shivay Y.S., Saxena A.K. (2014). Evaluating the efficacy of cyanobacterial formulations and biofilmed inoculants for leguminous crops. Arch. Agron. Soil Sci..

[127] Bidyarani N., Prasanna R., Babu S., Hossain F., Saxena A.K. (2016). Enhancement of plant growth and yields in Chickpea (*Cicer arietinum* L.) through novel cyanobacterial and biofilmed inoculants. Microbiol. Res..

[128] Manjunath M., Kanchan A., Ranjan K., Venkatachalam S., Prasanna R., Ramakrishnan B., Hossain F., Nain L., Shivay Y.S., Rai A.B. (2016). Beneficial cyanobacteria and eubacteria synergistically enhance bioavailability of soil nutrients and yield of okra. Heliyon.

[129] Rana A., Kabi S.R., Verma S., Adak A., Pal M., Shivay Y.S., Prasanna R., Nain L. (2015). Prospecting plant growth-promoting bacteria and cyanobacteria as options for enrichment of macro- and micronutrients in grains in rice-wheat cropping sequence. Cogent Food Agric..

[130] Babu S., Prasanna R., Bidyarani N., Nain L., Shivay Y.S. (2015). Synergistic action of PGP agents and Rhizobium spp. for improved plant growth, nutrient mobilization and yields in different leguminous crops. Biocatal. Agric. Biotechnol..

[131] Duan H., Liu W., Zhou L., Han B., Huo S., El-Sheekh M., Dong H., Li X., Xu T., Elshobary M. (2023). Improving saline-alkali soil and promoting wheat growth by co-applying potassium-solubilizing bacteria and cyanobacteria produced from brewery wastewater. Front. Environ. Sci..

[132] Manjunath M., Prasanna R., Sharma P., Nain L., Singh R. (2011). Developing PGPR consortia using novel genera Providencia and Alcaligenes along with cyanobacteria for wheat. Arch. Agron. Soil Sci..

[133] Lamérand C., Shirokova L.S., Bénézeth P., Rols J.-L., Pokrovsky O.S. (2020). Olivine Dissolution and Hydrous Mg Carbonate and Silicate Precipitation in the Presence of Microbial Consortium of Photo-Autotrophic and Heterotrophic Bacteria. Geochim. Cosmochim. Acta.

[134] Deng Y., Mauri M., Vallet M., Staudinger M., Allen R.J., Pohnert G. (2022). Dynamic diatom-bacteria consortia in synthetic plankton communities. Appl. Environ. Microbiol..

[135] Chegukrishnamurthi M., Shekh A., Ravi S., Mudliar S.N. (2022). Volatile organic compounds involved in the communication of microalgae-bacterial association extracted through headspace-solid phase microextraction and confirmed using gas chromatography-mass spectrophotometry. Bioresour. Technol..

[136] Astafyeva Y., Gurschke M., Streit W.R., Krohn I. (2022). Interplay between the microalgae *Micrasterias radians* and its symbiont *Dyadobacter* sp. HH091. Front. Microbiol..

[137] Liu J., Wu Y., Wu C., Muylaert K., Vyverman W., Yu H.-Q., Muñoz R., Rittmann B. (2017). Advanced Nutrient Removal from Surface Water by a Consortium of Attached Microalgae and Bacteria: A Review. Bioresour. Technol..

[138] Zhang J., Huang B., Tang T. (2022). Effect of coculture with *Halomonas mongoliensis* on *Dunaliella salina* growth and phenol degradation. Front. Bioeng. Biotechnol..

[139] Cheah Y.T., Chan D.J.C. (2022). A methodological review on the characterization of microalgal biofilm and its extracellular polymeric substances. J. Appl. Microbiol..

[140] Tong C.Y., Chan D.J.C. (2023). Novel extrapolymeric substances biocoating on polyvinylidene fluoride membrane for enhanced attached growth of *Navicula incerta*. Microb. Ecol..

[141] Lacroux J., Trably E., Bernet N., Steyer J.-P., van Lis R. (2020). Mixotrophic Growth of Microalgae on Volatile Fatty Acids Is Determined by Their Undissociated Form. Algal Res..

[142] Lacroux J., Jouannais P., Atteia A., Bonnafous A., Trably E., Steyer J.-P., van Lis R. (2022). Microalgae Screening for Heterotrophic and Mixotrophic Growth on Butyrate. Algal Res..

[143] Lacroux J., Mahieux M., Llamas M., Bonnafous A., Trably E., Steyer J.-P., van Lis R. (2024). Mixotrophic Cultivation of Microalgae-Bacteria Consortia Enhances Dark Fermentation Effluent Treatment. Bioresour. Technol..

[144] Proietti Tocca G., Agostino V., Menin B., Tommasi T., Fino D., Di Caprio F. (2024). Mixotrophic and Heterotrophic Growth of Microalgae Using Acetate from Different Production Processes. Rev. Environ. Sci. Biotechnol..

[145] Traver-Azuara J., Giner C.R., García-Comas C., Sánchez-Zurano A., Ciardi M., Acién G., Bondarenko S., Obiol A., Massana R., Sala M.M. (2025). The Complex Interplay between Microalgae and the Microbiome in Production Raceways. bioRxiv.

[146] Naseema Rasheed R., Pourbakhtiar A., Mehdizadeh Allaf M., Baharlooeian M., Rafiei N., Alishah Aratboni H., Morones-Ramirez J.R., Winck F.V. (2023). Microalgal Co-Cultivation -Recent Methods, Trends in Omic-Studies, Applications, and Future Challenges. Front. Bioeng. Biotechnol..

[147] Vo T.P., Danaee S., Chaiwong C., Pham B.T., Poddar N., Kim M., Kuzhiumparambil U., Songsomboon C., Pernice M., Ngo H.H. (2024). Microalgae-bacteria consortia for organic pollutants remediation from wastewater: A critical review. J. Environ. Chem. Eng..

[148] Glick B.R. (2012). Plant Growth-Promoting Bacteria: Mechanisms and Applications. Scientifica.

[149] Soumare A., Boubekri K., Lyamlouli K., Hafidi M., Ouhdouch Y., Kouisni L. (2020). From Isolation of Phosphate Solubilizing Microbes to Their Formulation and Use as Biofertilizers: Status and Needs. Front. Bioeng. Biotechnol..

[150] Sahu P.K., Brahmaprakash G.P. (2016). Formulations of Biofertilizers—Approaches and Advances. Microbial Inoculants in Sustainable Agricultural Productivity.

[151] O’Callaghan M., Ballard R.A., Wright D. (2022). Soil microbial inoculants for sustainable agriculture: Limitations and opportunities. Soil Use Manag..

[152] Khan A., Singh A.V., Gautam S.S., Agarwal A., Punetha A., Upadhayay V.K., Kukreti B., Bundela V., Jugran A.K., Goel R. (2023). Microbial bioformulation: A microbial-assisted biostimulating fertilization technique for sustainable agriculture. Front. Plant Sci..

[153] Díaz-Rodríguez A.M., Parra-Cota F.I., Cira-Chávez L.A., García-Ortega L.F., Estrada-Alvarado M.I., Santoyo G., de los Santos-Villalobos S. (2025). Microbial inoculants in sustainable agriculture: Advancements, challenges, and future directions. Plants.

[154] Fadiji A.E., Xiong C., Egidi E., Singh B.K. (2024). Formulation challenges associated with microbial biofertilizers in sustainable agriculture and paths forward. J. Sustain. Agric. Environ..

[155] Rojas-Sánchez B., Guzmán-Guzmán P., Morales-Cedeño L.R., Orozco-Mosqueda M.C., Saucedo-Martínez B.C., Sánchez-Yáñez J.M., Fadiji A.E., Babalola O.O., Glick B.R., Santoyo G. (2022). Bioencapsulation of microbial inoculants: Mechanisms, formulation types and application techniques. Appl. Biosci..

[156] Luft L., Mazutti M.A. (2025). Freeze and spray drying technologies to produce solid microbial formulations for sustainable agriculture. Processes.

[157] Hu M., Hei R.N., Guo D.J., Luo J., Lu C., Xu W.L., Zhang Z.Y., Xiao Q.B., Ma Y. (2023). Shelf-life enhancement of bio-inoculants through synergistic effects of encapsulation technology and osmotic protectants. J. Environ. Chem. Eng..

[158] Safari M., Motamedi E., Kari Dolatabad H., Modarres Sanavy S.A.M. (2020). Nano-Carriers Effects on the Viability and Efficiency of Pseudomonas Strains as Phosphate-Solubilizing Bacteria. Heliyon.

[159] Kaur R., Kaur S., Dwibedi V., Kaur C., Akhtar N., Alzahrani A. (2023). Development and Characterization of Rice Bran-Gum Arabic Based Encapsulated Biofertilizer for Enhanced Shelf Life and Controlled Bacterial Release. Front. Microbiol..

[160] Isabel J.B., Balamurugan A., Renuka Devi P., Periyasamy S. (2024). Chitosan-encapsulated microbial biofertilizer: A breakthrough for enhanced tomato crop productivity. Int. J. Biol. Macromol..

[161] Graciano V.A., Lodi L.A., Vasconcellos V.M., Farinas C.S. (2025). Dynamics of encapsulated *Bacillus subtilis* in soil under salinity and temperature stresses. Biocatal. Agric. Biotechnol..

[162] Miranda A.M., Hernandez-Tenorio F., Villalta F., Vargas G.J., Sáez A.A. (2024). Advances in the development of biofertilizers and biostimulants from microalgae. Biology.

[163] Hernández A., González-Moya M., Márquez A., Acevedo L.E. (2024). Review microalgae drying: A comprehensive exploration from conventional air drying to microwave drying methods. Future Foods.

[164] Gao J.-N., Xu M.-T., Uwiringiyimana E. (2025). Isolation of highly efficient potassium solubilizing bacteria and their effects on nutrient acquisition and growth promotion in tobacco seedlings. BMC Plant Biol..

[165] Dineshkumar R., Kumaravel R., Gopalsamy J., Sikder M.N.A., Sampathkumar P. (2018). Microalgae as Bio-Fertilizers for Rice Growth and Seed Yield Productivity. Waste Biomass Valorization.

[166] Suchithra M.R., Muniswami D.M., Suba Sri M., Usha R., Ahamed Rasheeq A., Antrose Preethi B., Dineshkumar R. (2022). Effectiveness of Green Microalgae as Biostimulants and Biofertilizer through Foliar Spray and Soil Drench Method for Tomato Cultivation. S. Afr. J. Bot..

[167] Ma F., Li Y., Han X., Li K., Zhao M., Guo L., Li S., Wang K., Qin K., Duan J. (2024). Microalgae-Based Biofertilizer Improves Fruit Yield and Controls Greenhouse Gas Emissions in a Hawthorn Orchard. PLoS ONE.

[168] Žunić V., Hajnal-Jafari T., Stamenov D., Djurić S., Tomić J., Pešaković M., Grohar M.C., Stampar F., Veberic R., Hudina M. (2024). Application of Microalgae-Based Biostimulants in Sustainable Strawberry Production. J. Appl. Phycol..

[169] Braun J.C.A., Colla L.M. (2023). Use of Microalgae for the Development of Biofertilizers and Biostimulants. Bioenergy Res..

[170] Parmar P., Kumar R., Neha Y., Srivatsan V. (2023). Microalgae as next Generation Plant Growth Additives: Functions, Applications, Challenges and Circular Bioeconomy Based Solutions. Front. Plant Sci..

[171] Kolli Venkata S., Behera B., Paramasivan B. (2020). Efficacy of Microalgal Extracts as Biostimulants through Seed Treatment and Foliar Spray for Tomato Cultivation. Ind. Crops Prod..

[172] Brazil Ministry of Agriculture, Livestock and Food Supply Normative Instruction No. 61 of 8 July 2020. https://www.gov.br/agricultura/pt-br/assuntos/insumos-agropecuarios/insumos-agricolas/fertilizantes/legislacao/in-61-de-8-7-2020-organicos-e-biofertilizantes-dou-15-7-20.pdf.

[173] Brazil Ministry of Agriculture, Livestock and Food Supply MAPA Ordinance No. 52 of 15 March 2021. https://www.gov.br/agricultura/pt-br/assuntos/sustentabilidade/organicos/legislacao/portugues/PORTARIAMAPAN52.2021.pdf.

[174] Presidência da República of Brazil Law No. 15.070 of 23 December 2024. https://www.planalto.gov.br/ccivil_03/_ato2023-2026/2024/lei/l15070.htm.

[175] Brazil Ministry of Agriculture, Livestock and Food Supply Normative Instruction No. 46 of 6 October 2011. https://www.gov.br/mme/pt-br/arquivos/do-07-10-2011-s1.pdf.

[176] dos Reis G.A., Martínez-Burgos W.J., Pozzan R., Pastrana-Puche Y., Ocán-Torres D., de Queiroz Fonseca Mota P., Rodrigues C., Lima Serra J., Scapini T., Karp S.G. (2024). Comprehensive review of microbial inoculants: Agricultural applications, technology trends in patents, and regulatory frameworks. Sustainability.

[177] Santos F., Melkani S., Oliveira-Paiva C., Bini D., Pavuluri K., Gatiboni L., Mahmud A., Torres M., McLamore E., Bhadha J.H. (2024). Biofertilizer use in the United States: Definition, regulation, and prospects. Appl. Microbiol. Biotechnol..

[178] Gonçalves J., Freitas J., Fernandes I., Silva P. (2023). Microalgae as Biofertilizers: A Sustainable Way to Improve Soil Fertility and Plant Growth. Sustainability.

[179] Osorio-Reyes J.G., Valenzuela-Amaro H.M., Pizaña-Aranda J.J.P., Ramírez-Gamboa D., Meléndez-Sánchez E.R., López-Arellanes M.E., Castañeda-Antonio M.D., Coronado-Apodaca K.G., Gomes Araújo R., Sosa-Hernández J.E. (2023). Microalgae-Based Biotechnology as Alternative Biofertilizers for Soil Enhancement and Carbon Footprint Reduction: Advantages and Implications. Mar. Drugs.

[180] Romero-García J.M., González-López C.V., Brindley C., Fernández-Sevilla J.M., Acién-Fernández F.G. (2022). Simulation and Techno-Economical Evaluation of a Microalgal Biofertilizer Production Process. Biology.

[181] Castro J.S., Ferreira J., Magalhães I.B., Jesus Junior M.M., Marangon B.B., Pereira A.S.A.P., Lorentz J.F., Gama R.C.N., Rodrigues F.A., Calijuri M.L. (2023). Life cycle assessment and techno-economic analysis for biofuel and biofertilizer recovery as by-products from microalgae. Renew. Sustain. Energy Rev..

[182] Kuhn C.E.S., de Sousa J.V.L., Menezes B.C., Gomes A.C.F. (2025). Spatial Analysis of the Feasibility of Using Rock Powder as Fertilizer in Agriculture in Mato Grosso in Brazil. Sustainability.

